# Regorafenib plus nivolumab in unresectable hepatocellular carcinoma: the phase 2 RENOBATE trial

**DOI:** 10.1038/s41591-024-02824-y

**Published:** 2024-02-19

**Authors:** Hyung-Don Kim, Seyoung Jung, Ho Yeong Lim, Baek-Yeol Ryoo, Min-Hee Ryu, Samuel Chuah, Hong Jae Chon, Beodeul Kang, Jung Yong Hong, Han Chu Lee, Deok-Bog Moon, Ki-Hun Kim, Tae Won Kim, David Tai, Valerie Chew, Jeong Seok Lee, Richard S. Finn, June-Young Koh, Changhoon Yoo

**Affiliations:** 1 Department of Oncology, Asan Medical Center, University of Ulsan College of Medicine, Seoul, Republic of Korea; 2 Genome Insight, Inc., San Diego, La Jolla, CA USA; 3 Graduate School of Medical Science and Engineering, Korea Advanced Institute of Science and Technology, Daejeon, Republic of Korea; 4 Division of Hematology-Oncology, Department of Medicine, Samsung Medical Center, Sungkyunkwan University School of Medicine, Seoul, Republic of Korea; 5 Translational Immunology Institute, SingHealth-Duke-NUS Academic Medical Centre, Duke-NUS Medical School, Singapore, Singapore; 6 Department of Medical Oncology, CHA Bundang Medical Center, CHA University School of Medicine, Seongnam, Republic of Korea; 7 Department of Gastroenterology, Asan Medical Center, University of Ulsan College of Medicine, Seoul, Republic of Korea; 8 Department of Surgery, Asan Medical Center, University of Ulsan College of Medicine, Seoul, Republic of Korea; 9 Division of Medical Oncology, National Cancer Centre, Singapore, Singapore; 10 Division of Hematology-Oncology, Geffen School of Medicine at UCLA, Los Angeles, CA USA

**Keywords:** Liver cancer, Tumour immunology

## Abstract

Regorafenib has anti-tumor activity in patients with unresectable hepatocellular carcinoma (uHCC) with potential immunomodulatory effects, suggesting that its combination with immune checkpoint inhibitor may have clinically meaningful benefits in patients with uHCC. The multicenter, single-arm, phase 2 RENOBATE trial tested regorafenib–nivolumab as front-line treatment for uHCC. Forty-two patients received nivolumab 480 mg every 4 weeks and regorafenib 80 mg daily (3-weeks-on/1-week-off schedule). The primary endpoint was the investigator-assessed objective response rate (ORR) per Response Evaluation Criteria in Solid Tumors (RECIST) version 1.1. The secondary endpoints included safety, progression-free survival (PFS) and overall survival (OS). ORR per RECIST version 1.1 was 31.0%, meeting the primary endpoint. The most common adverse events were palmar-plantar erythrodysesthesia syndrome (38.1%), alopecia (26.2%) and skin rash (23.8%). Median PFS was 7.38 months. The 1-year OS rate was 80.5%, and the median OS was not reached. Exploratory single-cell RNA sequencing analyses of peripheral blood mononuclear cells showed that long-term responders exhibited T cell receptor repertoire diversification, enrichment of genes representing immunotherapy responsiveness in *MKI67*^+^ proliferating CD8^+^ T cells and a higher probability of M1-directed monocyte polarization. Our data support further clinical development of the regorafenib–nivolumab combination as front-line treatment for uHCC and provide preliminary insights on immune biomarkers of response. ClinicalTrials.gov identifier: NCT04310709.

## Abstract (web summary)

In the phase 2 RENOBATE trial, the combination of regorafenib and nivolumab as first-line treatment for unresectable hepatocellular carcinoma exhibited a safe profile and met the primary endpoint with a 31% overall response rate.

## Main

In global phase 3 trials, anti-PD-1 antibody monotherapy administered in the first-line^1^ and second-line^2^ settings has failed to improve overall survival (OS) among patients with unresectable hepatocellular carcinoma (uHCC). However, the phase 3 IMBrave 150 trial^3^ and the HIMALAYA study^4^ demonstrated the OS benefits of first-line combination therapy with anti-PD-L1 plus anti-VEGF monoclonal antibody (atezolizumab–bevacizumab) and anti-PD-L1 plus CTLA-4 (durvalumab–tremelimumab: STRIDE) compared to sorafenib, prompting approval of these combination treatments. Moreover, a recent first-line phase 3 trial revealed that a combination of the VEGFR2-targeted tyrosine kinase inhibitor rivoceranib, plus the anti-PD-1 antibody camrelizumab, yielded significantly improved progression-free survival (PFS) and OS compared to sorafenib^5^. However, other first-line phase 3 trials of anti-PD-1/PD-L1 plus VEGF-targeted multi-kinase inhibitor (MKI) combinations have not shown survival benefits compared to MKI monotherapy. In the COSMIC-312 study^6^, cabozantinib–atezolizumab improved PFS but failed to show OS benefit compared to sorafenib, and, in the LEAP-002 trial^7^, lenvatinib–pembrolizumab did not improve PFS and OS compared to lenvatinib. These discrepancies have not yet been explained, but the results suggest that clinical efficacy may partly depend on differences in the detailed molecular and/or immunological mechanisms underlying the effects of the combination partners, especially in the context of immune checkpoint inhibitor (ICI)-based combination treatment.

Regorafenib is an MKI with anti-angiogenic activity, which inhibits various targets and reportedly improves survival outcomes in patients with uHCC, after progression with sorafenib treatment^8,9^. Notably, regorafenib modulates the VEGFR and CSF1R pathways, suggesting that it might be able to reverse the immunosuppressive gradients of myeloid cells and, thereby, potentiate anti-tumor immune responses. In particular, CSF1R pathway inhibition could polarize myeloid cells toward exerting anti-tumor responses^10–12^. Myeloid cells are critically implicated in the immune evasion process in HCC^13–15^ and in the ICI response^16^; thus, there is a strong rationale for combining regorafenib and ICI in patients with uHCC. In the CheckMate 459 study, nivolumab monotherapy in the first-line setting was associated with a trend toward improved survival outcomes compared to sorafenib, but it did not meet the pre-defined statistical significance for improving survival^1^. Anti-tumor activity and potential immunomodulatory effects of regorafenib suggest that its combination with nivolumab may have clinically meaningful benefits in patients with uHCC.

Several biomarkers are reportedly associated with clinical outcomes among patients with uHCC treated with ICI^17–19^. However, none has yet been firmly established as predictive for ICI response. Thus, there remains a need to further investigate the detailed mechanisms underlying response or resistance to ICI-based treatments.

The RENOBATE trial is a multicenter phase 2 study evaluating the efficacy and safety of the regorafenib–nivolumab combination, in the first-line setting, in patients with uHCC. In this report, we present the results in terms of clinical outcomes and a comprehensive biomarker study, including analyses of circulating tumor DNA (ctDNA), single-cell RNA sequencing (scRNA-seq), single-cell T cell receptor (TCR) repertoire and multicolor flow cytometry with serially collected peripheral blood mononuclear cells (PBMCs).

## Results

### Patient characteristics

**Fig. 1:**
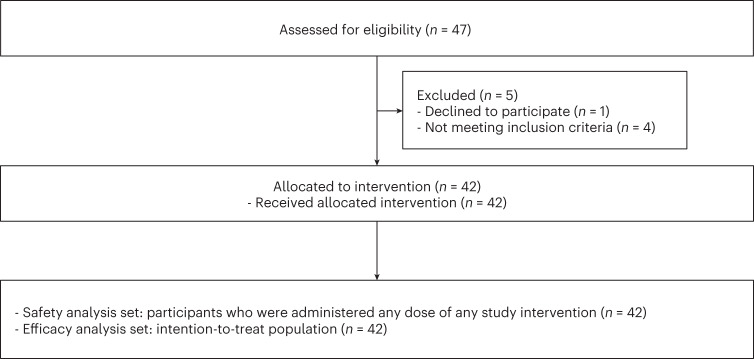
**CONSORT flow diagram**.

Among the 47 patients assessed for eligibility, 42 were enrolled between 24 July 2020 and 16 February 2021 and received study treatment (CONSORT diagram; Fig. 1). The median age was 61 years (range, 40–79 years), and 31 patients (73.8%) were male (Table 1). Most patients had Barcelona Clinic Liver Cancer (BCLC) stage C (*n* = 37, 88.1%), hepatitis B virus (HBV) infection as an etiology of HCC (*n* = 30, 71.4%) and prior transarterial chemoembolization (*n* = 35, 83.3%).

**Table 1:** Baseline patient characteristics

Characteristics	(*n* = 42)
Age, years	61 (40–79)
Male sex	31 (73.8%)
Liver cirrhosis	31 (73.8%)
Tumor stage	
BCLC B	5 (11.9%)
BCLC C	37 (88.1%)
Etiology	
HBV	30 (71.4%)
HCV	5 (11.9%)
Others	7 (16.7%)
Extrahepatic spread	
Lung	20 (47.6%)
Lymph node	11 (26.2%)
Peritoneum	8 (19.1%)
Bone	5 (11.9%)
Other	4 (9.5%)
Previous treatment for HCC	
Surgical resection	21 (50.0%)
Radiation therapy	12 (28.6%)
Transarterial chemoembolization	35 (83.3%)
Radiofrequency ablation	5 (11.9%)
Transarterial embolization	1 (2.4%)
Transarterial radioembolization	1 (2.4%)

Data are presented as *n* (%) or median (range).

### Primary outcome

**Fig. 2:**
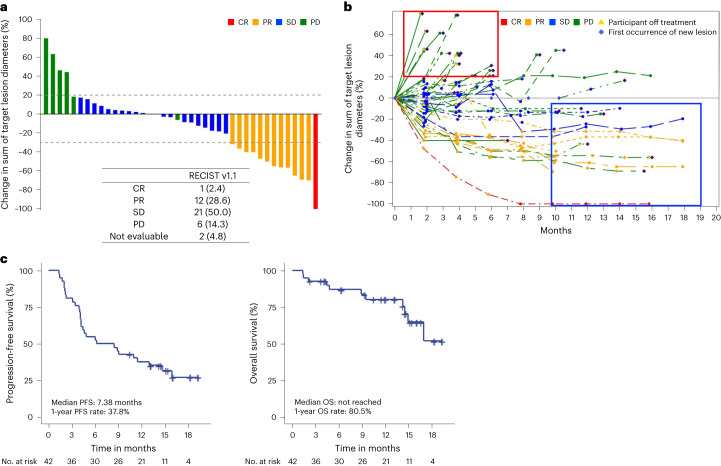
Clinical outcomes of regorafenib–nivolumab. **a**, Waterfall plot showing the change in the sum of target lesion diameters. **b**, Spider plot showing changes in the sum of target lesion diameters throughout treatment. Red box represents early progressors. Blue box represents long-term responders. **c**, PFS and OS of the study population.

The primary endpoint was the investigator-assessed objective response rate (ORR) per Response Evaluation Criteria in Solid Tumors (RECIST) version 1.1 in the intention-to-treat population. The ORR per RECIST version 1.1 was 31.0%, meeting the primary endpoint (ORR of ≥25%). Complete response (CR) was achieved in one patient (2.4%) and partial response (PR) in 12 patients (28.6%) (Fig. 2a,b). Twenty-one (50.0%) and six (14.3%) patients had stable disease (SD) and progressive disease (PD) as best response, respectively, and tumor response was not assessable in two patients (4.8%).

### Secondary outcomes

As a pre-specified secondary endpoint per protocol, the investigator-assessed ORR per modified RECIST was 33.3%, with CR achieved in two patients (4.8%) and PR in 12 patients (28.6%). With a median follow-up duration of 11.1 months (95% confidence interval (CI), 6.11–14.0 months), 29 events occurred for PFS and 11 events for OS. The median PFS was 7.38 months (95% CI, 4.12–13.0 months), and the 1-year PFS rate was 37.8% (Fig. 2c). The 1-year OS rate was 80.5% (95% CI, 63.0–90.3%), and the median OS was not reached. In post hoc analysis, the median duration of response per RECIST version 1.1 (defined as the time from CR or PR to disease progression or death) was 10.3 months (95% CI, 8.2–13.9 months).

### Safety

#### Adverse events

Table 2 and Extended Data Table 1 summarize the profiles of adverse events that occurred in ≥5% of patients and overall study patients, respectively (regardless of their relation to study treatment). The most common adverse events were palmar-plantar erythrodysesthesia syndrome (*n* = 16, 38.1%), alopecia (*n* = 11, 26.2%) and skin rash (*n* = 10, 23.8%). Grade 3 adverse events were noted in 10 patients (23.8%), with the most frequent grade 3 adverse event being increased aspartate aminotransferase (AST) (*n* = 2, 4.8%). No patient suffered a grade 4 adverse event or treatment-related death.

**Table 2:** Adverse events that occurred in ≥5% of patients

Adverse events^a^ by preferred terms^b^	Any grade *n* (%)	Grade 3–4 *n* (%)
Any adverse events	39 (92.9)	10 (23.8)^c^
Palmar-plantar erythrodysesthesia syndrome	16 (38.1)	0 (0.0)
Abdominal pain	13 (31.0)	0 (0.0)
Alopecia	11 (26.2)	0 (0.0)
Skin rash	10 (23.8)	0 (0.0)
Fatigue	10 (23.8)	0 (0.0)
AST increased	10 (23.8)	2 (4.8)
Pyrexia	7 (16.7)	0 (0.0)
Dyspepsia	7 (16.7)	0 (0.0)
Decreased appetite	6 (14.3)	0 (0.0)
Dysphonia	5 (11.9)	0 (0.0)
Hypothyroidism	5 (11.9)	0 (0.0)
Diarrhea	5 (11.9)	0 (0.0)
Cough	5 (11.9)	0 (0.0)
Pruritus	4 (9.5)	0 (0.0)
Stomatitis	4 (9.5)	0 (0.0)
ALT increased	4 (9.5)	0 (0.0)
Hypoalbuminemia	4 (9.5)	1 (2.4)
Abdominal distension	4 (9.5)	0 (0.0)
Body weight decreased	4 (9.5)	0 (0.0)
Back pain	4 (9.5)	0 (0.0)
Proteinuria	4 (9.5)	0 (0.0)
Platelet count decreased	3 (7.1)	0 (0.0)
Urticaria	3 (7.1)	0 (0.0)
Insomnia	3 (7.1)	0 (0.0)

^a^The adverse events reported here were all events and were not limited to those considered to be causally related to study treatment.

^b^Preferred terms were defined according to the Medical Dictionary for Regulatory Activities terminology version 24.0. All participants who were administered any dose of any study intervention were included for safety analysis. Adverse event severity was scored using the NCI-CTCAE version 5.0.

^c^Grade 3 adverse events occurred in 10 patients, and three patients experienced two or more kinds of grade 3 adverse events. Other grade 3 adverse events not listed in the table include ascites (*n* = 1), gastrointestinal hemorrhage (*n* = 2), hyperkalemia (*n* = 1), hyperglycemia (*n* = 1), hypotension (*n* = 1), acute kidney injury (*n* = 2), pneumonia (*n* = 1), cardiovascular accident (*n* = 1) and jaundice (*n* = 1).

#### Study drug discontinuation and dose reduction

No patient discontinued both regorafenib and nivolumab due to adverse events. In four patients (9.5%), adverse events led to discontinuation of one component of the combination: three patients discontinued regorafenib due to fatigue, proteinuria and palmar-plantar erythrodysesthesia syndrome, and one patient discontinued nivolumab due to decreased renal function. The median duration of adherence to at least one study agent was 7.36 months (range, 0.89–19.76 months), and the median duration of adherence to the full combination was 5.61 months (range, 0.89–19.76 months).

A total of eight patients (19.0%) experienced adverse events requiring regorafenib dose reduction. The protocol did not allow dose reduction of nivolumab. The adverse events that most frequently led to regorafenib dose reduction were palmar-plantar erythrodysesthesia syndrome (*n* = 2) followed by proteinuria, general weakness, pancreatitis, skin rash, decreased platelet count and increased serum bilirubin (*n* = 1 each).

### Exploratory outcomes

#### ctDNA analysis

**Extended Data Fig. 1:**
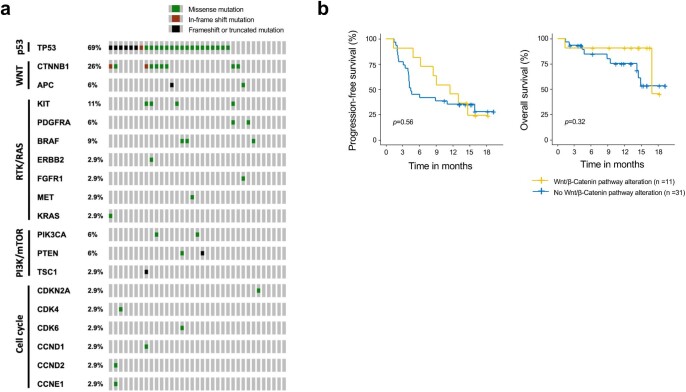
Circulating tumor DNA analysis. **a**, Heatmap of genetic alterations detected using Guardant360 CDx. **b**, Progression-free survival and overall survival according to the presence of genetic alterations in the Wnt/β-Catenin pathway, evaluated by circulating tumor DNA analysis. Two-sided Log-rank test was used for comparison.

ctDNA analysis revealed that the most frequently mutated gene was *TP53* (69%) followed by *CTNNB1* (26%) (Extended Data Fig. 1a). Altered Wnt/β-catenin pathway genes were not associated with poor survival outcomes (Extended Data Fig. 1b).

#### Increased frequency of classical monocytes upon treatment

**Fig. 3:**
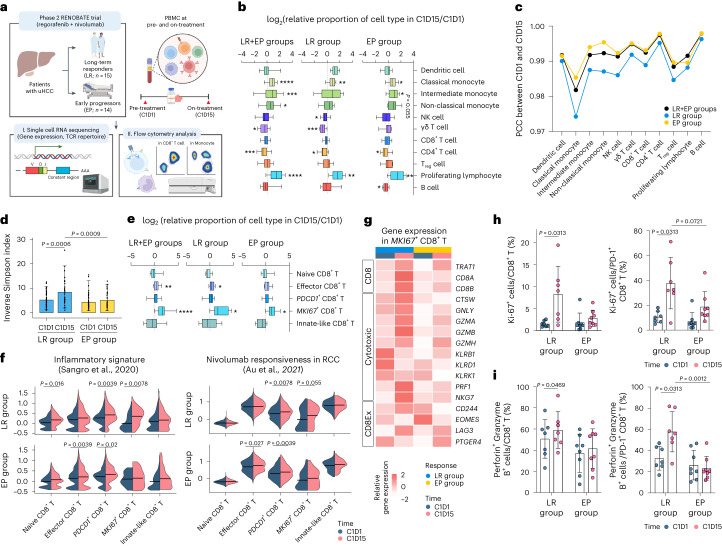
Overall immune landscapes and dynamic changes of CD8^+^ T cell subsets in peripheral blood. **a**, Schematic summary of the design of exploratory analyses. **b**, Box plots showing the fold change in proportions of immune cell types among PBMCs in long-term responders (LR; *n* = 9), early progressors (EP; *n* = 9) and both (*n* = 18). **c**, PCC of each cell type between C1D1 and C1D15. **d**, Bar plots showing the TCR diversity, as represented by the inverse Simpson index for CD8^+^ T cells in LR (*n* = 15) and EP (*n* = 14) between C1D1 and C1D15. **e**, Box plots showing the fold change in the proportions of CD8^+^ T cell subclusters among NK/T cells among LR (*n* = 9), EP (*n* = 9) and both (*n* = 18). **f**, Violin plots showing the module score for gene sets related to inflammatory signature (left) and nivolumab responsiveness in each CD8^+^ T cell cluster (right). **g**, Heat map showing expression levels of CD8 and genes related to cytotoxicity and T cell exhaustion among *MKI67*^+^CD8^+^ T cells. **h**, Proportion of Ki-67^+^ cells among CD8^+^ T cells and PD-1^+^CD8^+^ T cells, before and after treatment, among LR (*n* = 7) and EP (*n* = 8). **i**, Proportion of Granzyme B^+^perforin^+^ cells among CD8^+^ T cells and PD-1^+^CD8^+^ T cells, before and after treatment, among LR (*n* = 7) and EP (*n* = 8). Gating strategy of CD8^+^ T cells is shown in Extended Data Fig. 5c. *P < 0.05, **P < 0.01, ***P < 0.005, ****P < 0.001, according to a Wilcoxon signed-rank test for paired groups and two-tailed Mann–Whitney *U*-test for unpaired groups. Data are presented as mean ± s.d. In each box plot, the box represents the interquartile range, and whiskers represent minima and maxima. RCC, renal cell carcinoma.

We performed scRNA-seq of PBMC samples collected at baseline (cycle 1, day 1 (C1D1)) and on-treatment (cycle 1, day 15 (C1D15)) (Fig. 3a). Patients were divided into two clinical subgroups: those with disease progression at the first evaluation or progressively increased tumor burden (early progressors, *n* = 14) and those with a decreased tumor burden lasting at least 10 months (long-term responders, *n* = 15) (Fig. 3b).

**Extended Data Fig. 2:**
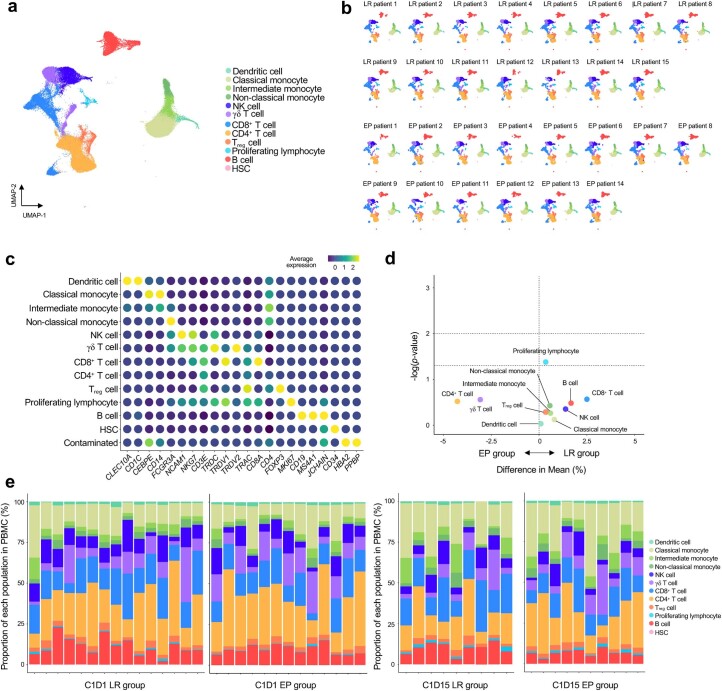
Individual response profiles of peripheral blood mononuclear cells (PBMCs). **a**, UMAP projections of PBMC (n = 86,205) colored according to immune cell type. **b**, UMAP projections of PBMCs splitted by each patient. **c**, Dot plots showing the average normalized expressions of marker genes in each immune cell cluster. **d**, Volcano plot showing proportional changes of each cell type among PBMCs at C1D1, between long-term responders (LR; *n* = 15) and early progressors (EP; *n* = 13). The unpaired T test was performed for unpaired groups. **e**, Bar plots showing the proportion of each cell type among PBMCs in individual samples: LR at C1D1 (*n* = 15); EP at C1D1 (*n* = 13); LR at C1D15 (*n* = 9); and EP at C1D15 (*n* = 10).

**Extended Data Fig. 3:**
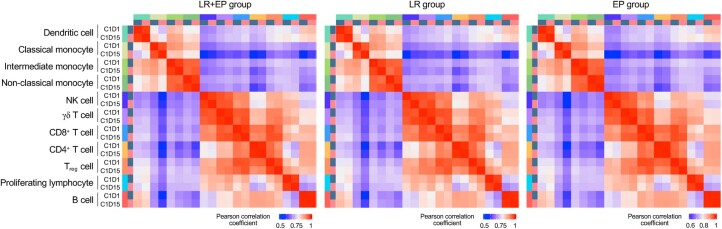
**Heatmap showing the Pearson correlation coefficient of each cell type upon regorafenib-nivolumab treatment**.

Unsupervised clustering identified 13 distinct immune subsets (Extended Data Fig. 2a–c). During regorafenib–nivolumab treatment, the proportions of classical monocytes and proliferating lymphocytes increased in both clinical subgroups, whereas the proportion of intermediate monocytes increased only among early progressors (Fig. 3b and Extended Data Fig. 2d,e). Pearson correlation coefficient (PCC) analysis revealed that classical monocytes exhibited the greatest difference in gene expression profiles between C1D1 and C1D15 (Fig. 3c and Extended Data Fig. 3), more prominently among long-term responders.

#### Dynamic changes of CD8^+^ T cells in long-term responders

**Extended Data Fig. 4:**
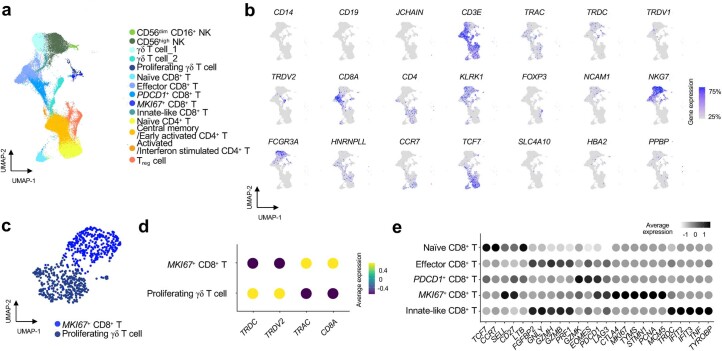
Subcluster analysis of T/NK lymphocyte populations. **a**, UMAP of T/NK cells colored according to immune cell types. **b**, UMAP plots showing normalized expressions of marker genes used for subclustering of T/NK cells. **c**, UMAP plots of proliferating lymphocytes colored according to immune cell types. **d**, Dot plots showing the average normalized expressions of marker genes used for subclustering of proliferating lymphocytes. **e**, Dot plots showing the average normalized expression of marker genes in CD8^+^ T-cell subclusters.

**Extended Data Fig. 5:**
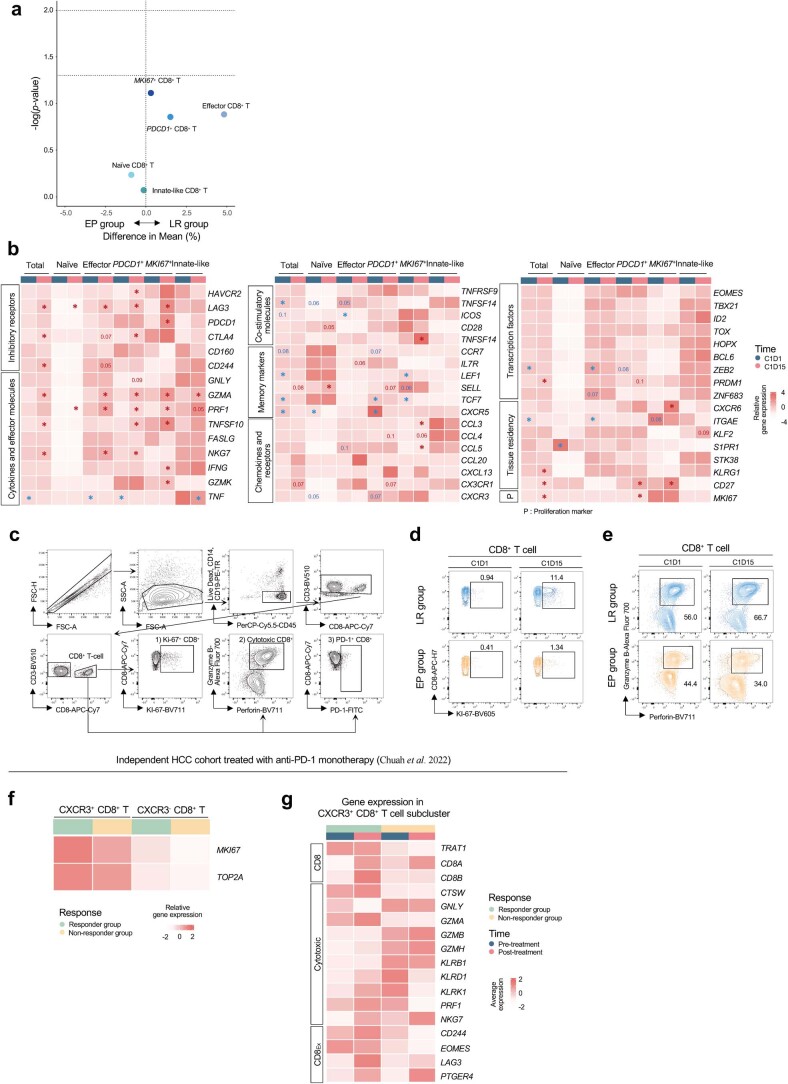
Detailed response profiles of CD8^+^ T cells upon regorafenib-nivolumab treatment. **a**, Volcano plot showing proportional changes in total CD8^+^ T-cell subclusters at C1D1 between long-term responders (LR; *n* = 15) and early progressors (EP; *n* = 13). The unpaired T test was performed for unpaired groups. **b**, Heatmap of CD8^+^ T surrogate marker genes among total CD8^+^ T cells and subclusters. Red and blue astrerisks indicate genes significantly upregulated at C1D1 and C1D15, respectively. **c**, Gating strategy of CD8^+^ T-cells PBMC in LR and EP. **d**, Representative flow cytometry plots for KI-67-expressing cells among CD8^+^ T-cells. **e**, Representative flow cytometry plots for granzyme B- and perforin-expressing CD8^+^ T-cells. **f**, Heatmap of proliferation marker gene among *CXCR3*^+^CD8^+^ T-cell and *CXCR3*^−^CD8^+^ T-cell subclusters from an independent HCC cohort treated with anti-PD-1 monotherapy (Chuah et al. 2022). **g**, Heatmap of CD8^+^ T surrogate marker genes among *CXCR3*^+^CD8^+^ T-cell subcluster from the independent HCC cohort treated with anti-PD-1 monotherapy (Chuah et al. 2022). *P < 0.05, according to a two-sided Wilcoxon signed-rank test for paired groups.

Only among long-term responders, regorafenib–nivolumab treatment increased the diversity of TCR clones of CD8^+^ T cells, as represented by increased inversed Simpson index (Fig. 3d). Unsupervised clustering of natural killer (NK)/T cells (Extended Data Fig. 4a,b), including proliferating lymphocytes (Extended Data Fig. 4c,d), revealed five distinct CD8^+^ T cell subclusters: naive CD8^+^ T cells, effector CD8^+^ T cells overexpressing genes encoding cytotoxic molecules, *PDCD1*^+^CD8^+^ T cells overexpressing *PDCD1*, *MKI67*^+^ proliferating CD8^+^ T cells and innate-like effector CD8^+^ T cells (Extended Data Fig. 4e). Among these CD8^+^ T cell subsets, effector CD8^+^ and *MKI67*^*+*^ proliferating CD8^+^ T cells exhibited significantly increased frequencies in the overall population throughout treatment, more prominently among long-term responders (Fig. 3e and Extended Data Fig. 5a).

*MKI67*^+^ proliferating CD8^+^ T cells exhibited enrichment of an inflammatory signature associated with favorable clinical outcomes after nivolumab monotherapy in uHCC^17^ and a signature representing transcriptomic changes after nivolumab monotherapy among responders^20^. These changes were more prominent among long-term responders (Fig. 3f). On C1D15, cytotoxicity-related genes were upregulated on *MKI67*^+^ proliferating CD8^+^ T cells, only among long-term responders (Fig. 3g and Extended Data Fig. 5b). These findings were validated by flow cytometry, where only long-term responders showed significantly increased proportions of Ki-67^+^ (Fig. 3h and Extended Data Fig. 5c,d) and Granzyme B^+^perforin^+^ cells among CD8^+^ T cells and PD-1^+^CD8^+^ T cells, respectively (Fig. 3i and Extended Data Fig. 5e). Analyzing an independent scRNA-seq dataset from patients with uHCC treated with anti-PD-1 monotherapy^21^ revealed enrichment of cytotoxic features among *CXCR3*^+^CD8^+^ T cells, the key cell subset associated with response to anti-PD-1 therapy^21^. This enrichment was observed in responders after anti-PD-1 monotherapy and not among non-responders (Extended Data Fig. 5f,g).

#### Preferential M1-directed polarization in long-term responders

**Fig. 4:**
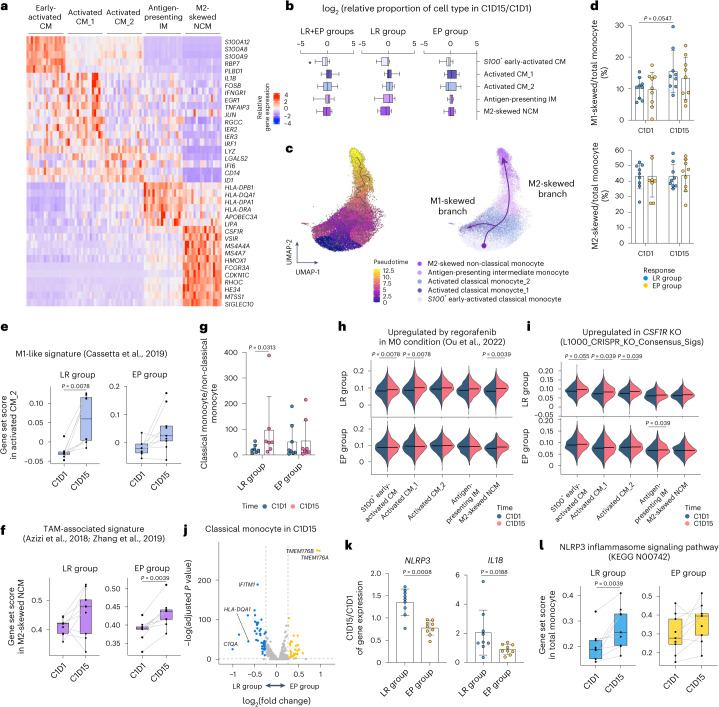
Dynamic monocyte response. **a**, Heat map of cluster-specific DEGs of monocyte clusters. **b**, Fold changes in the proportions of monocyte subclusters among myeloid cells among long-term responders (LR; *n* = 9), early progressors (EP; *n* = 9) and both (*n* = 18). **c**, UMAP and pseudotime trajectory initiated from early-activated classical monocytes toward either M1-skewed or M2-skewed route. **d**, The proportions of M1-skewed or M2-skewed monocytes among total monocytes among LR (*n* = 9) and EP (*n* = 9). **e**, Module scores for an M1-like gene set within the activated classical monocyte_2 subset in LR (*n* = 8) and EP (*n* = 9). **f**, Module scores for a gene set related to TAM among M2-skewed non-classical monocytes in LR (*n* = 9) and EP (*n* = 9). **g**, Ratio of classical to non-classical monocytes among LR (*n* = 7) and EP (*n* = 8). **h**, Module scores for the gene set upregulated by regorafenib in each monocyte cluster from LR (*n* = 9) and EP (*n* = 9). **i**, Module scores for the gene set upregulated in CSF1R_Up_from_L1000_CRISPR_KO_Consensus_Sigs in each monocyte cluster from LR (*n* = 9) and EP (*n* = 9). **j**, DEGs among total monocytes on C1D15 in LR (*n* = 9) versus EP (*n* = 10). Blue and yellow indicate genes significantly upregulated in LR and EP, respectively. **k**, Changes in the expression of *NLRP3* and *IL18* in LR (*n* = 9) and EP (*n* = 9). **l**, Module score for the NLRP3 inflammasome signaling pathway-related gene set in total monocytes of LR (*n* = 9) and EP (*n* = 9). Gating strategy of monocytes is shown in Extended Data Fig. 6b. *P < 0.05, according to a Wilcoxon signed-rank test for paired groups and two-tailed Mann–Whitney *U*-test for unpaired groups. Data are presented as mean ± s.d. In each box plot, the box represents the interquartile range, and whiskers represent minima and maxima. CM, classical monocyte; IM, indeterminate monocyte; KO, knockout; NCM, non-classical monocyte.

**Extended Data Fig. 6:**
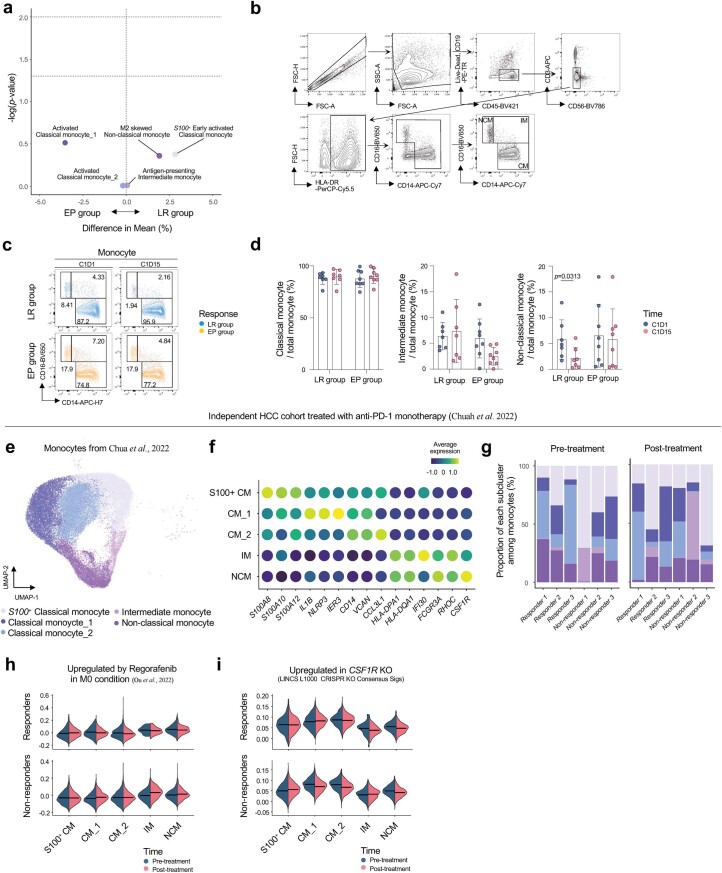
Detailed response profiles of monocytes upon regorafenib-nivolumab treatment. **a**, Volcano plot showing proportional changes in monocyte subclusters at C1D1 among long-term responders (LR; *n* = 15) versus early progressors (EP; *n* = 13). The unpaired T test was performed for unpaired groups. **b**, Gating strategy of monocytes from PBMCs in LR and EP. **c**, Representative flow cytometry plots for classical (CD14^+^CD16^−^), intermediate (CD14^+^CD16^+^) and non-classical (CD14^dim^CD16^+^) monocytes among total monocytes. **d**, Proportions of classical, intermediate and non-classical monocytes among total monocytes, before and after regorafenib-nivolumab treatment, in LR (*n* = 7) and EP (*n* = 8). Analyses of monocyte clusters from an independent HCC cohort treated with anti-PD-1 monotherapy (Chuah et al. 2022) **(e-i)**. **e**, UMAP of monocyte clusters. **f**, Dot plots showing the average normalized expressions of marker genes used for subclustering of monocyte clusters. **g**, Bar plots showing the proportion of each cell type among monocyte clusters in individual samples. **(h-i)**, Violin plots showing module scores for the gene sets upregulated in each monocyte cluster according to treatment outcomes at pre- and post-treatment; gene sets upregulated in by regorafenib in M0 condition **(h)**, upregulated in CSF1R KO **(i)**. *P < 0.05, **P < 0.01, ***P < 0.005, ****P < 0.001, according to a two-sided Wilcoxon signed-rank test for paired groups, and two-sided Mann-Whitney U test for unpaired groups. Data are presented as mean ± standard deviation.

We identified five distinct monocyte clusters: S100^+^ early-activated classical monocytes; activated classical monocyte_1; activated classical monocyte_2; antigen-presenting indeterminate monocytes; and M2-skewed non-classical monocytes overexpressing genes involved in immunosuppression, including *CSF1R*, *SIGLEC10* and *VSIR* (Fig. 4a). The relative abundances of these monocyte populations did not significantly change with treatment (Fig. 4b and Extended Data Fig. 6a).

Differentiation pseudotime analysis revealed diverging trajectories. The classical monocyte_2 cluster was the bifurcation point—with one trajectory toward the activated classical monocyte_1 cluster (immunostimulatory M1-like branch) and the other toward M2-skewed non-classical monocytes (immunosuppressive M2-like branch) (Fig. 4c). Upon regorafenib–nivolumab treatment, the proportion of M1 branch cells among total monocytes increased only in long-term responders, whereas the proportion of M2 branch cells did not significantly change in either clinical subgroup (Fig. 4d). At the bifurcation point, the expression level of the M1-like gene signature^22^ significantly increased, more prominently in long-term responders (Fig. 4e). Expression of the tumor-associated macrophage (TAM) signature^23^ was significantly increased in M2-skewed non-classical monocytes, only among early progressors (Fig. 4f). Flow cytometry analysis revealed a significant increase in the relative abundance of CD14^+^ classical monocytes to CD14^low^CD16^high^ non-classical monocytes, only among long-term responders (Fig. 4g and Extended Data Fig. 6b–d).

Throughout regorafenib–nivolumab treatment, classical monocyte subsets exhibited significantly increased expression levels of a regorafenib-responsive gene set derived from an HCC model^12^ and of a gene signature representing CSF1R knockout, only among long-term responders and not early progressors (Fig. 4h,i). Within the independent HCC cohort treated with anti-PD-1 monotherapy^21^ (Extended Data Fig. 6e,f), both responders and non-responders exhibited no changes either in the proportion of subcluster (Extended Data Fig. 6g) or in the expression levels of gene set module scores related to regorafenib responsiveness or CSF1R knockout after anti-PD-1 therapy (Extended Data Fig. 6h,i).

#### Interaction between CD8^+^ T cells and classical monocytes

**Extended Data Fig. 7:**
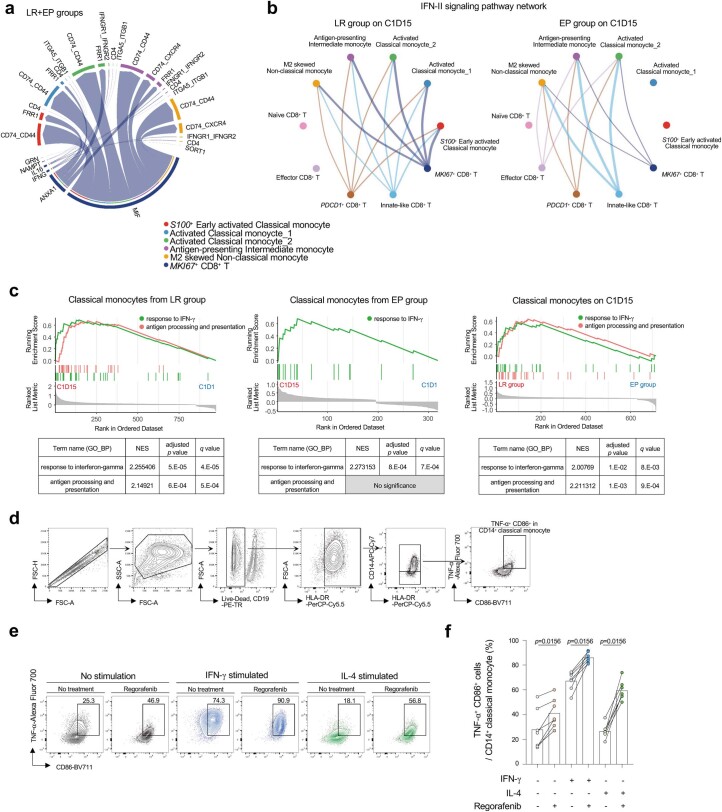
Communication between proliferating CD8^+^ T cells and classical monocytes with enrichment of genes of antigen processing and presentation and IFN responses. **a**, A chord diagram to visualize cell-cell communication between *MKI67*^+^ effector CD8^+^ T-cells and monocyte subclusters among long-term responders (LR; *n* = 15) and early progressors (EP; *n* = 13). **b**, Dot plots showing the associations of latent patterns with cell groups in the IFN-II signaling pathway network, between LR (*n* = 9) and EP (*n* = 10) on C1D15. **c**, GSEA plots of gene modules related to antigen processing and presentation and response to IFN-γ in classical monocytes, with DEGs (adjusted *p* value *≤* 0.05) from each clinical setting: LR between C1D1 and C1D15 (left column), EP between C1D1 and C1D15 (middle column) and classical monocytes at C1D15 between LR and EP (right column). Significance based on enrichment analysis with Benjamini-Hochberg-adjusted *p* values. **d**, Gating strategy of TNF-α^+^ CD86^+^ cells among CD14^+^ classical monocytes of healthy donors. **e**, Representative flow cytometry plots for TNF-α^+^ CD86^+^ cells among CD14^+^ classical monocytes after combination of IFN-γ (100 ng/ml), IL-4 (50 ng/ml), and regorafenib (1 μM) stimulation. **f**, Bar plots showing the proportion of TNF-α^+^ CD86^+^ cells among CD14^+^ classical monocytes of healthy donors (*n* = 7). *P < 0.05, **P < 0.01, ***P < 0.005, ****P < 0.001, according to a two-sided Wilcoxon signed-rank test for paired groups. Data are presented as mean ± standard deviation.

Interactome analysis revealed that proliferating CD8^+^ T cells provided interferon-gamma (IFN-γ)-related signals to the *S100*^*+*^ early-activated classical monocyte and activated classical monocyte_1 subsets among long-term responders (Extended Data Fig. 7a,b). We found no meaningful interaction between these two subsets among early progressors (Extended Data Fig. 7b).

On C1D15, only in long-term responders, we observed significant prominent enrichment of gene signatures related to IFN-γ in classical monocytic subsets and enrichment of a gene signature of antigen processing/presentation in classical monocytes (Extended Data Fig. 7c). An in vitro assay of sorted monocytes revealed that regorafenib treatment increased the frequency of TNF-α^+^CD86^+^ cells (representing M1-directed polarization^24^) among CD14^+^CD16^−^ classical monocytes, in the presence or absence of IFN-γ or interleukin (IL)-4 (Extended Data Fig. 7d–f).

#### Upregulation of *TMEM176A/B* on monocytes in early progressors

**Extended Data Fig. 8:**
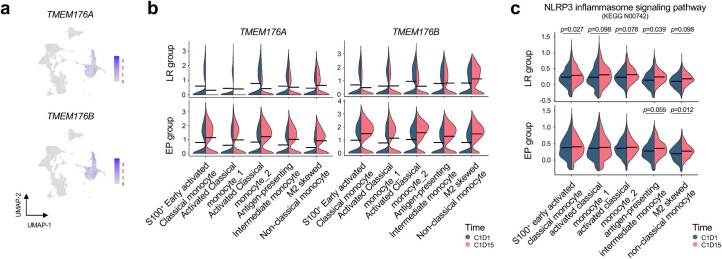
*TMEM176A/B* expression and expression levels of genes related to the NLRP3 inflammasome in monocytes upon regorafenib-nivolumab treatment. **a**, UMAP plots showing normalized expressions of *TMEM176A* and *TMEM176B* in PBMCs. **b**, Violin plots showing the average expression of the *TMEM176A* and *TMEM176B* genes before and after treatment among long-term responders (LR; *n* = 15) and early progressors (EP; *n* = 13). **c**, Violin plots showing the module score for the gene set related to the NLRP3 inflammasome signaling pathway in each monocyte subcluster in LR (*n* = 9) and EP (*n* = 9). *P < 0.05, **P < 0.01, ***P < 0.005, ****P < 0.001, according to a two-sided Wilcoxon signed-rank test for paired groups.

In classical monocytes on C1D15, *TMEM176A* and *TMEM176B* were the genes most significantly upregulated in early progressors versus long-term responders (Fig. 4j and Extended Data Fig. 8a,b). TMEM176B reportedly inhibits the inflammasome response and adversely affects responsiveness to ICIs^25^. Additionally, *NLRP3* and *IL18* were prominently upregulated throughout regorafenib–nivolumab treatment in long-term responders, not in early progressors (Fig. 4k). Accordingly, the expression levels of a gene set representing the NLRP3 inflammasome pathway were significantly increased throughout regorafenib–nivolumab treatment, prominently among long-term responders (Fig. 4l and Extended Data Fig. 8c).

## Discussion

In this phase 2 study, we investigated the clinical outcomes and dynamic immune landscapes associated with regorafenib–nivolumab as first-line therapy in patients with uHCC. The primary endpoint was met, with an ORR of 31.0% and a median PFS of 7.38 months. These efficacy outcomes were similar to those previously reported when using other first-line anti-PD-1/L1-VEGF-targeted MKI combinations^5–7^ or atezolizumab–bevacizumab^3^. Compared to the historical data with anti-PD-1/L1 monotherapy, these improved efficacy outcomes might be attributable to the combination of VEGFR-targeted agents with anti-PD-1/L1, as demonstrated in previous studies^5,7,26^. In the present RENOBATE trial, it is possible that the improved outcomes were partly due to the regorafenib-mediated modulation of myeloid cells (M1-directed polarization). Overall, regorafenib–nivolumab was well tolerated. The safety profiles were in line with those described in a previous phase 1 study of other types of gastrointestinal cancers^27^, and no new safety profile was noted for either regorafenib^8^ or nivolumab^1^. We observed a 24% incidence of grade 3–4 adverse events, which is favorable compared to the rates reported with other VEGF-targeted MKI plus anti-PD-1/L1 regimens (62–81%)^5–7^. This improvement may be attributable to the reduced dose of regorafenib (80 mg per day) in combination with ICI, whereas other regimens have used standard doses of MKIs as monotherapy. Our present results suggest that regorafenib–nivolumab may be a clinically feasible first-line treatment option in patients with uHCC. Combination regimens with ICIs and anti-angiogenic agents are now considered standard first-line therapy in uHCC, and our present data may provide insights into the biological or immunological implications of such combination therapy in patients with uHCC.

In the present study, the systematic collection of samples in a clinical trial setting provided an opportunity to explore the immune landscapes reprogrammed by regorafenib–nivolumab. To our knowledge, this is the first study to explore, at a single-cell level, the dynamic immune landscapes of patients with uHCC treated with first-line ICI-based combination therapy. We were unable to identify baseline predictors, which reflects the present difficulty of identifying predictive immune-related biomarkers^19^. Notably, our results imply the potential clinical relevance of diverging CD8^+^ T cell and classical monocyte responses, which may provide specific evidence to guide the future development of biomarkers and immunotherapeutic strategies for patients with uHCC.

We found that the characteristics of CD8^+^ T cells upon regorafenib–nivolumab treatment were associated with efficacy outcomes, solidifying the importance of T cell activation in the context of ICI-based treatment. The TCR repertoire diversification in long-term responders after regorafenib–nivolumab treatment provides preliminary evidence that favorable outcomes were associated with the clonal expansion of CD8^+^ T cells recognizing a variety of neoantigens to a level that was detectable in peripheral blood^20,28,29^. The association between TCR repertoire diversification and clinical outcomes with ICI-based treatments has mainly been described in ICI-responsive tumor types^20,28,29^, with only minimal comprehensive data available in HCC, particularly in the setting of a prospective trial. Our findings may not be specific to the regorafenib–nivolumab combination; in fact, our data indicate that this concept may be applicable to overall patients with uHCC treated with ICI-based combinations. The proliferative burst of CD8^+^ T cells observed among long-term responders is in line with previous studies highlighting its clinical value upon ICI-based treatment^30,31^.

Notably, the present study revealed diverging monocyte responses according to clinical outcomes. Among patients with uHCC treated with nivolumab monotherapy, we previously found that an increased frequency of classical monocytes after treatment was more prominent in patients with durable clinical benefit than in those without^32^. CD38^+^ macrophages have also been associated with favorable survival outcomes in patients with uHCC treated with anti-PD-1/PD-L1 (ref. ^33^). A recent single-cell analysis demonstrated that an abundance of CD11c^+^ antigen-presenting cells was associated with response to anti-PD-1 therapy among patients with uHCC^21^. In the present study, we showed interactions between proliferating *MKI67*^+^CD8^+^ T cells and classical monocyte subsets and a higher probability of M1-directed polarization in long-term responders. Additionally, upon regorafenib–nivolumab treatment, genes related to IFN-γ responses were enriched on classical monocytic subsets, more prominently among long-term responders than early progressors. Together with the enhanced M1-directed polarization by regorafenib shown in our in vitro assays, these findings further support that this functional monocytic response is associated with regorafenib–nivolumab treatment and highlight the clinical relevance of positive associations between IFN-γ response and myeloid populations. Notably, our results may validate the clinical relevance of the IFN-γ-directed myeloid response in the context of anti-PD-1/L1-MKI combinations, which warrants further investigation of relevant myeloid-related biomarkers in this setting.

In early progressors, we observed enrichment of a signature of immunosuppressive TAMs on M2-skewed non-classical monocytes, despite regorafenib–nivolumab treatment. This correlates with a previous report that immunosuppressive Arg-1-expressing CD163^+^ macrophages were enriched in patients with uHCC not responding to cabozantinib–nivolumab^34^. We examined these diverging monocyte responses in different clinical subgroups and found that *TMEM176A/B* was associated with early disease progression. TMEM176A/B is a surface protein highly expressed on myeloid immune cells, which was recently shown to prevent inflammasome responses, thereby preventing effective ICI-mediated immune responses^25^. Accordingly, regorafenib–nivolumab did not sufficiently increase the expression levels of NLRP3-related genes on monocytic populations in early progressors, possibly due to *TMEM176A/B* upregulation. This association between early disease progression and *TMEM176A/B* upregulation, coupled with an ineffective inflammasome response, suggests the possibility of developing novel immunotherapeutic strategies to augment the inflammasome-related response, to overcome primary resistance to ICI-based treatments in patients with uHCC. Notably, there remains a need for further functional validation of these findings regarding *TMEM176A/B*.

The clinical benefits of ICI-based combinations suggest that the combination partners exert different additive and/or synergistic effects. In a study of the atezolizumab–bevacizumab combination in patients with uHCC, the addition of bevacizumab to atezolizumab led to decreased VEGFR2 expression levels and frequency of regulatory T cells, yielding improved survival outcomes, particularly in patients with high VEGFR2 expression; increased frequency of regulatory T cells; and enrichment of myeloid inflammation signatures^19^. On the other hand, in a study of neoadjuvant cabozantinib–nivolumab for patients with uHCC, the addition of cabozantinib decreased the level of CXCL1 (a chemokine ligand involved in immune resistance) and subsequently promoted T cell activation^34^. Cabozantinib also reportedly induces intratumoral neutrophil infiltration, which further enhances the inflammatory phenotype, when used in combination with nivolumab in preclinical models of HCC^35^. In addition to its anti-angiogenic activity, regorafenib can potentially modulate myeloid cell populations by inhibiting the CSF-1/CSF-1R pathway^12,36^, which drives the immunosuppressive gradient of myeloid cells. Regorafenib-specific effects on classical monocytes were characterized by enrichment of a regorafenib-induced gene signature and of a gene signature representing CSF-1-deficient status. These effects were predominantly noted in long-term responders, highlighting the clinical relevance of the immunomodulatory effect of regorafenib. Our results indicate that regorafenib’s antagonistic effects on CSF1R might result in M1-directed polarization of monocytes, leading to enhanced anti-tumor immune response. The regorafenib–nivolumab combination is actively being investigated in other cancer types (NCT04879368), and regorafenib plus anti-PD-1/L1 combinations are being tested in patients with uHCC (NCT04718909 and NCT04183088).

The absence of a control group (that is, nivolumab monotherapy) may preclude accurate interpretation of the effects of adding regorafenib to nivolumab. Because anti-PD-1/L1 monotherapy and regorafenib monotherapy were not standard first-line treatment options at the time of study design, we did not include these treatments as control groups in our trial. Moreover, the subsequently reported results of the CheckMate 459 trial failed to show the superiority of nivolumab over sorafenib^1^, which also does not support the use of nivolumab monotherapy in a control arm. Our analyses of an external HCC cohort treated with anti-PD-1 monotherapy revealed a trend toward enrichment of cytotoxic features in *CXCR3*^+^CD8^+^ T cells among responders after anti-PD-1 monotherapy, which is in agreement with our findings regarding CD8^+^ T cell responses. However, patients treated with anti-PD-1 monotherapy did not exhibit the enrichment of gene signatures representing an M1-like signature, responsiveness to regorafenib or CSF1R knockout in monocyte subclusters from either responders or non-responders. This supports the concept that the addition of regorafenib promoted the modulation of myeloid cell responses, suggesting the modulation of myeloid cells due to this combination therapy. We cannot accurately compare the magnitudes of CD8^+^ T cell and monocyte responses; therefore, these results should be interpreted cautiously. This is due to the small number of patients analyzed for this comparison as well as the different clinical settings between the two study populations (that is, the line of therapy, definition of responders/non-responders and timepoint of acquiring on-treatment samples). Nevertheless, these results at least suggest that the dynamic responses of monocytes might be specifically present in patients with uHCC treated with the regorafenib–nivolumab combination and not in patients treated with anti-PD-1 monotherapy.

Alterations in the WNT/β-catenin pathway are reportedly associated with excluding anti-tumor immune responses, thereby conferring resistance to ICIs^37,38^. However, our ctDNA analysis revealed that genetic alterations in the Wnt/β-catenin pathway were not associated with poor survival outcomes. Similarly, among patients with uHCC treated with atezolizumab–bevacizumab, the survival outcomes were similar between patients with versus without mutation in *CTNNB1* (encoding β-catenin)^19^. The available data indicate that there is no reason to preclude patients with WNT/β-catenin pathway mutations from receiving ICI-based combinations involving anti-angiogenic agents. However, given the small numbers of patients involved in these analyses, further studies are warranted to delineate the clinical implications of mutations in the context of ICI-based combinations.

The present study had several limitations. Previous phase 3 trials demonstrated the superiority of other ICI-based combination regimens over sorafenib^3–5^, which may raise questions regarding the implications of our current clinical and biomarker analysis for regorafenib–nivolumab in uHCC. However, a regorafenib plus anti-PD-1 (pembrolizumab) regimen is currently under investigation in a global phase 3 trial, in comparison with transarterial chemoembolization or radioembolization, among patients with intermediate-stage HCC (REPLACE, NCT04777851). Although the design of this trial was not directly based on our findings, the present study may support the rationale of that study and provide translational insights that can help us understand the outcomes of that potentially practice-changing randomized trial. Notably, we could not provide data regarding differential mechanisms of action of regorafenib from other VEGF-targeted agents or how they might be implicated in different efficacy outcomes among these agents when combined with ICIs. Another limitation is that biomarker analyses were conducted as an exploratory endpoint. Due to the uncertainty of the clinical outcomes with this novel regimen at the time of study design, the subgroups for scRNA-seq were selected after the primary efficacy analysis. There remains a need for a functional study with a large well-designed patient population to validate the findings of our present analysis, considering the limitations of its exploratory nature and the challenges of scRNA-seq^39,40^. Other limitations of our study include the small sample size and lack of tissue-based correlatives. Finally, it should also be noted that the regorafenib-responsive gene sets may not fully recapitulate the effect of regorafenib in patients with HCC.

In conclusion, regorafenib–nivolumab has clinical activity and is a well-tolerated first-line treatment for patients with uHCC. Our exploratory biomarker analyses provide insights that may help us to understand the clinically relevant immune responses in this therapeutic context and identify potential targets to overcome resistance to ICI-based treatments. Regorafenib plus anti-PD-1/L1 should be further investigated for use in patients with HCC.

## Methods

### Study design and treatment

The open-label, multi-center, single-arm, phase 2 RENOBATE trial was conducted at three referral academic institutions in South Korea: Asan Medical Center, Samsung Medical Center and Bundang CHA Hospital. A total of 42 patients were enrolled between 24 July 2020 and 16 February 2021. Clinical data were collected by investigators and research coordinators in the eligible healthcare facilities. Key inclusion criteria included a diagnosis of HCC per American Association for the Study of Liver Diseases (AASLD) criteria; transarterial chemoembolization–unfeasible or refractory BCLC stage B or stage C; no prior systemic chemotherapy; age ≥19 years; Eastern Cooperative Oncology Group (ECOG) performance status 0 or 1; Child–Pugh class A; and at least one measurable lesion according to RECIST version 1.1. All patients provided written informed consent before enrollment, and the trial was registered at ClinicalTrials.gov (NCT04310709). The protocol was approved by the institutional review board (IRB) of each participating center (IRB no. 2019-0867 from Asan Medical Center; no. 2020-04-106 from Samsung Medical Center; and no. 2020-04-053 from CHA Bundang Medical Center). Regorafenib and nivolumab were supplied by Bayer and Ono Pharmaceuticals, respectively. Trial coordination, data management, site monitoring and statistical analysis for the clinical outcomes of the study were conducted by an external contract research organization (CMIC Korea). Data were collected using an electronic case report form. Site monitoring, data management and statistical analysis plans were approved by the principal investigator (C.Y.). This study was performed in accordance with the International Conference on Harmonization of Good Clinical Practice guidelines and the principles of the Declaration of Helsinki. Sex and/or gender were not considered in the trial design.

All inclusion and exclusion criteria were as follows:

#### Inclusion criteria

1. Age ≥19 years at the time of signing the informed consent form
2. Ability to comply with the study protocol, in the investigator’s judgment
3. HCC that was histologically/cytologically confirmed or clinically diagnosed according to AASLD criteria in cirrhotic patients. Histological confirmation of HCC was required in patients without liver cirrhosis.
4. Locally advanced unresectable or metastatic disease that was not amenable to curative surgical and/or locoregional therapies or that progressed after surgical and/or locoregional therapies
5. No prior systemic therapy for HCC
6. At least one measurable lesion (per RECIST version 1.1), confirmed by imaging within 28 d before initiation of study treatment
7. Patients who received prior local therapy (for example, radiofrequency ablation, percutaneous ethanol or acetic acid injection, cryoablation, high-intensity focused ultrasound, transarterial chemoembolization and transarterial embolization) were eligible, provided that other target lesion(s) had not been previously treated with local therapy or that the target lesion(s) within the field of local therapy had subsequently progressed in accordance with RECIST version 1.1.
8. Pre-treatment tumor tissue sample (if available)
  If tumor tissue was available, approximately 10−30 slides containing unstained, freshly cut, serial sections were required for subsequent translational research.
  If tumor tissue was not available (for example, depleted due to prior diagnostic testing), patients were still eligible.
9. ECOG performance status score 0 or 1
10. Child–Pugh class A
11. Adequate hematologic and end-organ function, defined by the following laboratory test results, obtained within 14 d before initiation of study treatment, unless otherwise specified:
  Absolute neutrophil count ≥ 1.0 × 10^9^ per L (1,000 per μl) without granulocyte colony-stimulating factor support
  Platelet count ≥ 75 × 10^9^ per L (75,000 per μl) without transfusion
  Hemoglobin ≥ 90 g L^−1^ (9 g dl^−1^). Patients could be transfused to meet this criterion.
  AST, ALT and ALP ≤ 3× the upper limit of normal (ULN)
  Serum bilirubin ≤ 2× ULN
  Serum creatinine ≤ 1.5× ULN or creatinine clearance ≥50 ml min^−1^ (calculated using the Cockcroft–Gault formula)
  Serum albumin ≥ 28 g L^−1^ (2.8 g dl^−1^)
  For patients not receiving therapeutic anti-coagulation: international normalized ratio (INR) or activated partial thromboplastin time (aPTT) ≤ 2× ULN
  Urine dipstick for proteinuria < 2+
  Patients found to have ≥2+ proteinuria on dipstick urinalysis at baseline underwent a 24-h urine collection and had to exhibit <1 g of protein in 24 h.
12. Resolution of any acute clinically significant treatment-related toxicity, of grade ≤1, from previous therapy, before study entry, with the exception of alopecia
13. Negative HIV result at screening test or on prior test conducted within 3 years
14. Documented virology status of hepatitis, as confirmed by screening HBV and HCV serology test
  Patients with active HBV must meet the following: HBV DNA < 500 IU ml^−1^ obtained within 14 d before initiation of study treatment, anti-HBV treatment (per local standard of care; for example, entecavir) for a minimum of 14 d before study entry and willingness to continue treatment for the length of the study.
15. Women of childbearing potential (including women with chemical menopause or no menstruation for other medical reasons)^#1^ had to agree to use contraception^#2^ from the time of informed consent until 5 months or more after the last dose of the investigational product. Also, women had to agree not to breastfeed from the time of informed consent until 5 months or more after the last dose of the investigational product.
16. Men had to agree to use contraception^#2^ from the start of study treatment until 7 months or more after the last dose of the investigational product.

#1. Women of childbearing potential were defined as all women after the onset of menstruation who were not postmenopausal and who had not been surgically sterilized (for example, hysterectomy, bilateral tubal ligation or bilateral oophorectomy). Postmenopause was defined as amenorrhea for 12 or more consecutive months without specific reasons. Women using oral contraceptives, intrauterine devices or mechanical contraception, such as contraceptive barriers, were regarded as having childbearing potential.

#2. The participant must consent to use any two of the following methods of contraception: vasectomy or condom for patients who are male or for a female participant’s partner and tubal ligation, contraceptive diaphragm, intrauterine device, spermicide or oral contraceptive for patients who are female or for a male participant’s partner.

#### Exclusion criteria

1. Patients who were diagnosed with fibrolamellar HCC, sarcomatoid HCC or combined type of cholangiocarcinoma and HCC
2. Patients with a history of malignancy other than HCC within 3 years before screening, with the exception of malignancies carrying a negligible risk of metastasis or death (for example, 5-year OS rate > 90%), such as adequately treated carcinoma in situ of the cervix, non-melanoma skin carcinoma, localized prostate cancer, ductal carcinoma in situ and stage I uterine cancer
3. Patients with a history of leptomeningeal seeding
4. Patients with symptomatic, untreated or actively progressing central nervous system (CNS) metastases
  Asymptomatic patients with treated CNS lesions are eligible, provided that all of the following criteria are met:
    1. The patients must have at least one measurable lesion, per RECIST version 1.1, other than CNS metastases.
    2. The patient must not have a history of intracranial hemorrhage or spinal cord hemorrhage.
    3. The metastatic lesions have to be limited in cerebellum or supratentorial region (for example, not to the midbrain, pons, medulla or spinal cord).
    4. There must be no evidence of interim progression between the completion of CNS-directed therapy and initiation of the study treatment.
    5. The patient must not undergo stereotactic radiotherapy within 7 d, whole-brain radiotherapy within 14 d or neurosurgical resection within 28 d before initiation of the study treatment.
    6. The patient must not have ongoing requirement for corticosteroids for CNS disease.
  Anti-convulsant therapy at a stable dose is permitted.
  Asymptomatic patients with CNS metastases newly detected at screening are eligible for the study after receiving radiotherapy or surgery, with no need to repeat the screening brain scan.
5. Patients with current or past history of autoimmune disease or immunodeficient disease (including, but not limited to, myasthenia gravis, myositis, autoimmune hepatitis, systemic lupus erythematosus, rheumatoid arthritis, inflammatory bowel disease, anti-phospholipid antibody syndrome, Wegener granulomatosis, Sjögren syndrome, Guillain–Barré syndrome or multiple sclerosis) with the following exceptions:
  Patients with autoimmune-related hypothyroidism who are on thyroid replacement hormone are eligible.
  Patients with controlled type 1 diabetes mellitus who are on an insulin regimen are eligible.
  Patients with eczema, psoriasis, lichen simplex chronicus or vitiligo with dermatologic manifestations only (for example, patients with psoriatic arthritis are excluded) are eligible for the study provided all of following conditions are met:
    1. Rash must cover less than 10% of body surface area.
    2. Disease has to be well controlled at baseline and requires only low-potency topical corticosteroids.
    3. There must be no occurrence of acute exacerbations of the underlying condition requiring psoralen plus ultraviolet A radiation, methotrexate, retinoids, biologic agents, oral calcineurin inhibitors or high-potency or oral corticosteroids within the previous 12 months.
6. Patients with current or past history of idiopathic pulmonary fibrosis, organizing pneumonia (for example, bronchiolitis obliterans), drug-induced pneumonitis or idiopathic pneumonitis or evidence of active pneumonitis on screening chest computed tomography (CT) scan
  Patients with history of radiation pneumonitis in the radiation field (fibrosis) are eligible if the radiation pneumonitis has been confirmed as stable (beyond acute phase) without any concerns about recurrence.
7. Patients who had experienced a transient ischemic attack, cerebrovascular accident, thrombosis or thromboembolism (pulmonary arterial embolism or deep vein thrombosis) within 6 months before initiation of study treatment
8. Patients with a history of uncontrollable or significant cardiovascular disease meeting any of the following criteria:
  Myocardial infarction within 6 months before initiation of study treatment
  Uncontrollable angina pectoris within 6 months before initiation of study treatment
  New York Heart Association class II or greater congestive heart failure within 6 months before initiation of study treatment
  Uncontrollable hypertension despite appropriate treatment (for example, systolic blood pressure ≥ 150 mmHg or diastolic blood pressure > 90 mmHg based on an average of three or more BP readings on two or more sessions)
  Arrhythmia requiring treatment
9. Patients with congenital long QT syndrome or corrected QT interval > 450 ms (calculated using the Fridericia method) at screening
10. Patients with systemic infections (including active tuberculosis) requiring treatment
11. Patients with history of hypertensive crisis or hypertensive encephalopathy
12. Patients with significant vascular disease (for example, aortic aneurysm requiring surgical repair or recent peripheral arterial thrombosis) within 6 months before initiation of study treatment
13. Patients who underwent major surgical procedure, other than for diagnosis, within 4 weeks before initiation of study treatment or who were expected to need a major surgical procedure during the study
14. Patients who had received radiotherapy within 28 d before initiation or radiotherapy to bone metastases within 14 d before initiation
15. Patients with prior history of allogeneic stem cell or solid organ transplantation
16. Patients with current or past history of severe allergic anaphylactic reactions to chimeric or humanized antibodies or fusion proteins
17. Patients with untreated or incompletely treated varices with active bleeding or high risk for bleeding
18. Patients with moderate or severe ascites
19. Patients with history of hepatic encephalopathy
20. Patients with evidence of bleeding diathesis or significant coagulopathy (in the absence of therapeutic anti-coagulation)
21. Patients who had recently (within 10 d of the first dose of study treatment) used aspirin (>300 mg per day) or treatment with dipyramidole, ticlopidine, clopidogrel and cilostazol
22. Patients who had recently used full-dose oral or parenteral anti-coagulants or thrombolytic agents for a therapeutic (as opposed to prophylactic) purpose
  Prophylactic anti-coagulation for the patency of venous access devices is allowed, provided the activity of the agent results in an INR < 1.5× ULN and aPTT within normal limits within 14 d before initiation of study treatment.
  Prophylactic use of low-molecular-weight heparin (that is, enoxaparin 40 mg per day) is allowed.
23. Patients who were treated with strong CYP3A4 inducers within 14 d before initiation of study treatment, including rifampin (and its analogues) or St. John’s wort
24. Patients who had previously received CD137 agonists or immune checkpoint blockade therapies, including anti-CTLA-4, anti-PD-1 and anti-PD-L1 therapeutic antibodies
25. Patients who were treated with systemic immunostimulatory agents (including, but not limited to, interferon and IL-2) within 4 weeks or 5 half-lives of the drug (whichever is longer) before initiation of study treatment
26. Patients who were treated with systemic immunosuppressive medication (including, but not limited to, corticosteroids, cyclophosphamide, azathioprine, methotrexate, thalidomide and anti-TNF-α agents) within 2 weeks before initiation of study treatment or anticipation of need for systemic immunosuppressive medication during study treatment, with the following exceptions:
  Patients who received temporary, low-dose systemic immunosuppressant medication or a one-time pulse dose of systemic immunosuppressant medication (for example, 48 h of corticosteroids for a contrast allergy) are eligible for the study.
  Patients who received mineralocorticoids (for example, fludrocortisone) or corticosteroids for chronic obstructive pulmonary disease (COPD) or asthma or low-dose corticosteroids for orthostatic hypotension or adrenal insufficiency are eligible for the study.
27. Patients who had abdominal or tracheoesophageal fistula, gastrointestinal perforation or intra-abdominal abscess within 6 months before initiation of study treatment
28. Patients who had intestinal obstruction and/or clinical signs or symptoms of gastrointestinal obstruction, including sub-occlusive disease related to the underlying disease or requirement for routine parenteral hydration, parenteral nutrition or tube feeding within 6 months before initiation of study treatment
  Patients with signs/symptoms of sub-occlusive syndrome/intestinal obstruction at time of initial diagnosis may be enrolled if they had received definitive (surgical) treatment for symptom resolution.
29. Women who were pregnant or breastfeeding or possibly pregnant
30. Other patients judged by the investigator or sub-investigator to be inappropriate as participants in this study

### Study treatment

Eligible patients received nivolumab 480 mg on day 1 and regorafenib 80 mg once daily on days 1–21, every 4 weeks. This dosing schedule was based on the results of a prior phase 1b trial of regorafenib–nivolumab in patients with gastric and colorectal cancer^27^. Response was assessed every 8 weeks, according to RECIST version 1.1. Additional imaging was performed whenever PD was clinically indicated. Safety profiles were evaluated on days 1 and 15 of cycle 1 and on day 1 of each subsequent cycle.

### Statistical analysis

The primary endpoint was the ORR, according to RECIST version 1.1. Secondary endpoints included safety profiles according to the National Cancer Institute Common Terminology Criteria for Adverse Events (NCI-CTCAE) version 5.0., ORR per modified RECIST, PFS and OS. The exploratory endpoint was correlative biomarker analysis for efficacy outcomes using ctDNA sequencing, scRNA-seq and fluorescence-activated cell sorting (FACS) analysis.

Based on the assumption that regorafenib–nivolumab might improve the ORR to 25% (P1) compared to 7% with sorafenib (P0), this study had to include 35 patients, according to Fleming’s single-stage phase 2 design with a two-sided alpha of 0.05 and power of 90%. Anticipating a 15% rate of loss to follow-up, a total 42 patients were needed for this study.

Efficacy analysis was based on the intention-to-treat population, which included all assigned patients. The safety analysis set included all patients who received at least one dose of study treatment. The patient subgroups for analysis of exploratory endpoints were not pre-defined, due to the unexpected nature of efficacy outcomes with a novel therapeutic regimen, and were defined after the primary analysis for efficacy outcomes. Statistical analyses for clinical outcomes were performed by the designated biostatisticians in the contract research organization.

PFS was defined as the period from the start of study treatment to progression according to RECIST version 1.1 or death from any cause, whichever occurred first. OS was defined as the period from the initiation of treatment to death from any cause. The Kaplan–Meier method and the log-rank test were used to estimate and compare the survival distribution, respectively. For patients who did not experience PD or death, the date of censoring for PFS was the earliest of the following: (1) patients who did not experience an event (and were not otherwise censored) at the time of data cutoff or loss to follow-up were censored on the date of their last follow-up; (2) if there was no tumor assessment after starting study treatment, the patients were censored on the date of last clinical assessment; and (3) patients who received subsequent anti-cancer therapy before experiencing an event were censored at the date of their last clinical assessment before initiating subsequent therapy. For OS, if death was not confirmed at the time of data cutoff, patients were censored on the last date of confirmed survival.

### Exploratory analyses

As exploratory endpoints of this study, the protocol pre-specified comprehensive biomarker analyses using ctDNA, scRNA-seq and multicolor flow cytometry with plasma or PBMCs. All human samples for these exploratory biomarker analysis were prospectively collected per protocol. The criteria for patient selection were determined after the primary efficacy analysis. Early progressors were defined as patients who exhibited disease progression at the first evaluation or who showed a progressive increase in tumor burden (that is, PD at the first evaluation or SD with increased tumor size at the first evaluation and PD at the second evaluations) (*n* = 14). Long-term responders were defined as patients who showed a continuous decrease in tumor burden (that is, PR or SD with decreased tumor size), which lasted at least 10 months (*n* = 15).

### Sample processing

Peripheral blood samples were collected at baseline (C1D1) and on-treatment (C1D15, C2D1 and C3D1). PBMCs were isolated from whole blood using standard Histopaque (GE Healthcare) density gradient centrifugation.

### ctDNA analysis

Using baseline samples of all 42 patients, ctDNA was analyzed using the Guardant360 CDx platform (Guardant Health). Mutation status was assessed following laboratory standard operating procedures, Good Clinical Laboratory Practice guidelines and the manufacturer’s protocols. We used a previously validated plasma-based comprehensive cancer genotyping assay, applying orthogonal tissue-based and plasma-based methodologies^41^.

### Pre-processing and initial clustering of scRNA-seq data

A Chromium single-cell library was constructed using Chromium Next GEM Single Cell 5′ Reagent Kits version 2 (Dual Index, 10x Genomics). Sequencing was performed using a NovaSeq 6000 (Illumina) with 2 × 150-bp, 20,000 paired-end reads per captured cell. The sequenced data were demultiplexed using the mkfastq function (Cell Ranger, 10x Genomics, version 3.0.1) to generate FASTQ files. Next, the demultiplexed FASTQ files of gene expression were aligned to the reference human genome (GRCH38; 10x Cell Ranger reference GRCh38 version 3.0.0). The feature–barcode matrices of RNA expression were analyzed using the Seurat R package (version 4.1.1)^42^. For basic quality control, we de-convoluted the sample identity and filtered inter-individual multiplets using the demuxlet package^43^. Then, we filtered low-quality cells expressing mitochondrial genes in more than 5% of their total gene expression or more than 4,500 genes. We also excluded doublets that initially clustered with doublets annotated through the demuxlet package. Next, we constructed a batch effects-corrected transformed ‘integrated’ data matrix using the FindIntegrationAnchors function. Principal component analysis (PCA; RunPCA function) was carried out for dimensional reduction of the transformed ‘integrated’ data matrix. Then, the cells underwent unsupervised clustering, according to the shared nearest neighbor (SNN) graph (FindNeighbors function, using the top 50 principal components (PCs); FindClusters function, resolution = 0.2) and were visualized by uniform manifold approximation and projection (UMAP) using the top 50 PCs. For subclustering analysis, the count matrix of each subcluster was normalized and scaled and underwent unsupervised clustering using PCs, as described above (monocyte subcluster: PCs = 15, resolution = 0.4; NKT subcluster: PCs = 25, resolution = 0.4; proliferating lymphocyte subcluster: PCs = 50, resolution = 0.1).

### Characterization of each subcluster of scRNA-seq data

To identify marker genes, differentially expressed genes (DEGs) in each cluster relative to the other clusters were selected based on the Wilcoxon rank-sum test, using the FindAllMarkers function (parameter: log fold change compared to the other clusters > 0.25, >0.6 min.pct2 (minimum fraction of test genes detected in cells of other clusters) and Bonferroni-adjusted *P* < 0.05). To describe the characteristics of each subcluster, we performed gene set enrichment analysis by calculating the gene set module score (AddModule-Score in the Seurat package), combined score (enrichR)^44^ and enrichment score (GSEA version 4.2.3, Broad Institute), using publicly available gene sets, including Gene Ontology: Biological Process databases^45^ and KEGG^46^.

To qualitatively compare the effects of treatment on each cell type, we calculated the PCC of each cell type according to treatment. To this end, we calculated the average gene expressions in each cell type from C1D1 and C1D15 (AverageExpression in the Seurat package). Next, PCC was carried out (‘cor’ from the ‘stats’ package) and visualized in a heat map (Heatmap from the ComplexHeatmap package). To evaluate quantitative changes, we first calculated the proportion of each cell type in all patients and in the subgroups of long-term responders and early progressors. The fold enrichment in proportion was calculated by dividing the fraction of each cell type on C1D15 by that on C1D1, followed by log_2_ transformation. Statistical analysis between responders was conducted using the Wilcoxon signed-rank test for paired groups.

### Pseudotime and interactome analysis

To investigate dynamic changes of the monocyte immune subset, we exported cells from this subset for monocle’s standard analysis process (monocle3, version 1.3.1)^47^. CellDataSet objects were built based on normalized count (transformed ‘RNA’ data matrix) using the as.cell_data_set function. Subsequently, the learn_graph function (minimal_branch_len = 7, geodesic_distance_ratio = 0.5) and orderCells function (default option) were used to generate trajectories of the monocyte subset from our scRNA-seq data. We then performed downstream analysis of specific branches from trajectories, using the choose_graph_segments function.

To analyze intercellular communication among each cell type from the scRNA-seq data, we performed interactome analysis using the CellChat package (version 1.5.0)^48^. A CellChat object was built based on the normalized count, using the createCellChat function. Subsequently, overexpressed genes and the interaction between each cell type were calculated using the identifyOverExpressedGenes function (default option), the identifyOverExpressedInteractions function (default option), the computeCommunProb function (default option), the computeCommunProbPathway function (default option) and the aggregateNet function (default option), based on the CellChatDB database of literature-supported ligand–receptor interactions in humans.

### Flow cytometry

Cryopreserved PBMCs were thawed and stained with fluorochrome-conjugated antibodies for 15 min at room temperature. A Live/Dead Cell Stain Kit (Invitrogen) was used to exclude dead cells. For intracellular staining, the cells were fixed and permeabilized using a Foxp3/Transcription Factor Staining Buffer Kit (Invitrogen) for 15 min. Next, antibodies to label cytoplasmic proteins were added for another 15-min incubation. Flow cytometry was performed using an LSR II instrument (BD Biosciences), and the data were analyzed with FlowJo software version 10.4 (Tree Star).

The following directly conjugated, unconjugated or secondary antibodies were used to identify cell markers of CD8^+^ T cells in human PBMCs: rabbit anti-human TCF1/TCF7 at 1:100 dilution (C63D9, Cell Signaling Technology, 2203S), donkey anti-rabbit IgG at 1:100 dilution (Poly4064, BioLegend, 406410), mouse anti-human CD3-V500 at 1:100 dilution (UCHT1, BD Biosciences, 561416), mouse anti-human Ki-67-BV605 at 1:100 dilution (Ki-67, BioLegend, 350522), mouse anti-human Perforin-BV711 at 1:100 dilution (dG9, BioLegend, 308130), pembrolizumab (PD-1, Selleck Chemicals, A2005), mouse anti-human IgG4 Fc-FITC at 1:100 dilution (HP6025, Southern Biotech, 9200-02), mouse anti-human CD45-PerCP-Cy5.5 at 1:100 dilution (HI30, BD Biosciences, 564105), mouse anti-human CD14-PE-TR at 1:200 dilution (61D3, eBioscience, 61-0149-42), mouse anti-human CD19-PE-TR at 1:200 dilution (HIB19, eBioscience, 61-0199-42), mouse anti-human Granzyme B Alexa Fluor 700 at 1:100 dilution (GB11, BD Biosciences, 560213) and mouse anti-human CD8-APC-H7 at 1:100 dilution (SK1, BD Biosciences, 560179).

The following directly conjugated antibodies were used to identify cell markers of monocytes in human PBMCs and in vitro cultured human CD14^+^ monocytes: mouse anti-human CD45-V450 at 1:100 dilution (HI30, BD Biosciences, 560367), mouse anti-human CD11B-BV510 at 1:100 dilution (ICRF44, BD Biosciences, 563088), mouse anti-human CD16-BV650 at 1:100 dilution (BD Biosciences, 563692), mouse anti-human CD56-BV786 at 1:100 dilution (BD Biosciences, 564058), mouse anti-human HLA-DR-PerCP-Cy5.5 at 1:100 dilution (G46-6, BD Biosciences, 560652), mouse anti-human CD19-PE-TR at 1:200 dilution (HIB19, eBioscience, 61-0199-42), mouse anti-human CD3-APC at 1:100 dilution (UCHT1, BD Biosciences, 555335), mouse anti-human CD14-APC-H7 at 1:100 dilution (MφP9, BD Biosciences, 560180), mouse anti-human CD86-BV711 (2331, BD Biosciences, 563158) and mouse anti-human TNF Alexa Fluor 700 at 1:100 dilution (MAb11, BD Biosciences, 557996).

### In vitro assay for monocyte polarization

From the PBMCs of healthy donors (*n* = 7), monocytes were isolated via magnetic-activated cell sorting (MACS; Mitenyi Biotec) using CD14 MicroBeads (human; Miltenyi Biotec). To induce monocyte polarization, 1 × 10^6^ purified monocytes were incubated in 48-well plates for 24 h. M1 polarization was achieved by treating them with 100 ng ml^−1^ IFN-γ (PeproTech), whereas M2 polarization was induced with 50 ng ml^−1^ IL-4 (PeproTech) with or without 1 μM regorafenib (Selleck Chemicals). For intracellular cytokine staining, the cells were further treated with 100 ng ml^−1^ lipopolysaccharide (Sigma-Aldrich) and 0.2 μl of GolgiPlug Protein Transport Inhibitor (containing Brefeldin A) (BD Biosciences) for 4 h after polarization.

### scRNA-seq analysis of patients treated with anti-PD-1 monotherapy

We additionally analyzed the previously published scRNA-seq dataset from patients with HCC treated with anti-PD-1 monotherapy^21^. We used 12 scRNA-seq result sets from paired pre-treatment and post-treatment PBMC samples from responders (*n* = 3) and non-responders (*n* = 3), as previously defined (that is, responders showed PR or SD for ≥6 months, and non-responders showed PD within 6 months).

We subclustered monocyte clusters based on previously defined annotations and conducted subcluster analysis of monocyte clusters, as described above. We calculated a batch effects-corrected transformed ‘integrated’ data matrix, using the FindIntegrationAnchors function, considering the origin of the gem. PCA (RunPCA function) was performed to reduce the dimensionality of the transformed ‘integrated’ data matrix. Subsequently, unsupervised clustering of cells was conducted based on the SNN graph (FindNeighbors function, using the top 20 PCs; FindClusters function, resolution = 0.3), and the results were visualized by UMAP. Additionally, we analyzed the subset of CXCR3^+^CD8^+^ T effector memory (T_EM_) cells, which have been suggested to reflect responsiveness to anti-PD-1 monotherapy and to be recruited to the tumor microenvironment in patients with HCC^21^. We then analyzed the average expressions of genes associated with CD8^+^ T cell activation, following the same approach used in our scRNA-seq analyses for regorafenib–nivolumab treatment, as described above.

### Statistical analysis software

Statistical analyses were performed using SAS version 9.4, Prism version 9.4.1 and R version 3.4.1 software.

### Reporting summary

Further information on research design is available in the Nature Portfolio Reporting Summary linked to this article.

## Online content

Any methods, additional references, Nature Portfolio reporting summaries, source data, extended data, supplementary information, acknowledgements, peer review information; details of author contributions and competing interests; and statements of data and code availability are available at 10.1038/s41591-024-02824-y.

### Supplementary information

Supplementary Information Clinical trial protocol and statistical analysis plan.

Reporting Summary

## Figures and Tables

**Extended Data Table 1:**
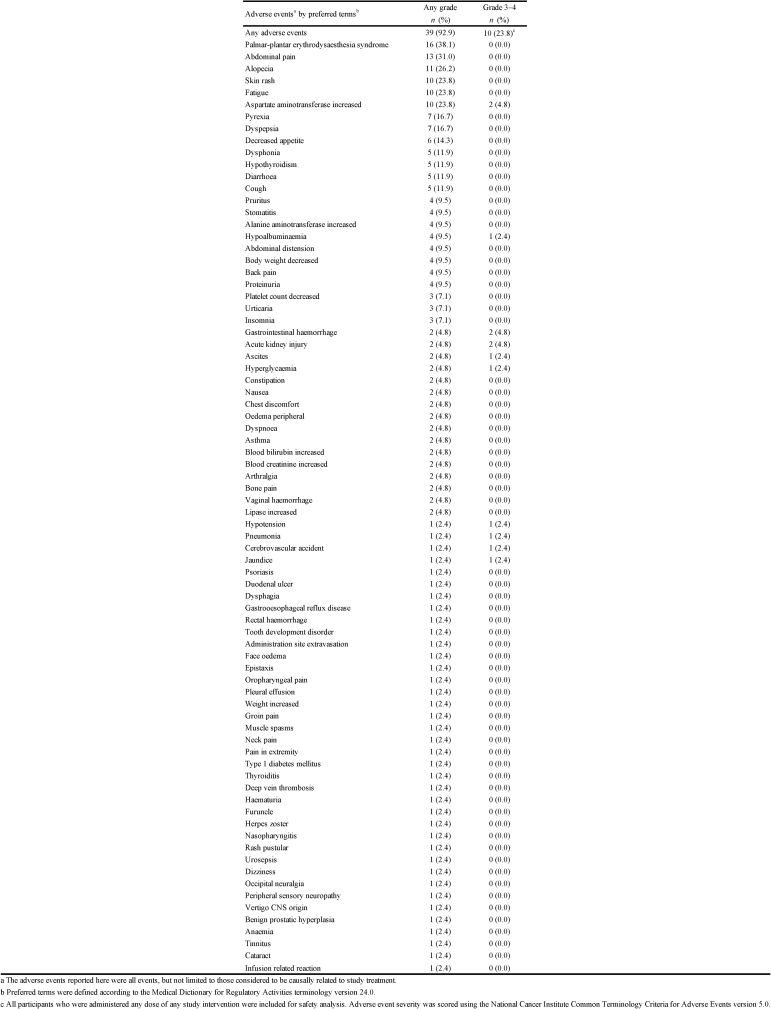
Adverse events that occurred in overall study patients

## Data Availability

Patient-related data not included in this paper were generated as part of the clinical trial and may be subject to patient confidentiality. All requests for raw and analyzed data and materials should be directed to C.Y. (yooc@amc.seoul.kr) and will be responded to within 4 weeks. The requests will be promptly reviewed by the Asan Medical Center to determine whether the request is subject to any intellectual property or confidentiality obligations. Any data and materials that can be shared will be released via a material transfer agreement. All raw data for single-cell sequencing were deposited in the Gene Expression Omnibus under accession number GSE243572. The trial protocol can be found in the Supplementary Information.

## Code availability

All analyses were conducted using publicly available software, as detailed in the Methods section. The raw scripts used to generate the scRNA-seq analysis figures presented in this paper are accessible via the Zenodo repository (10.5281/zenodo.8131764).
