# Supplementary material for: Regorafenib plus nivolumab in unresectable hepatocellular carcinoma: the phase 2 RENOBATE trial
Source: Nat Med. 2024 Feb 19;30(3):699–707. doi: 10.1038/s41591-024-02824-y (PMC10957471; doi:10.1038/s41591-024-02824-y)
Supplement: Supplementary file 1 — Clinical trial protocol and statistical analysis plan. [file 41591_2024_2824_MOESM1_ESM.pdf]

nature medicine

Article https://doi.org/10.1038/s41591-024-02824-y

# Regorafenib plus nivolumab in unresectable hepatocellular carcinoma: the phase 2 RENOBATE trial

In the format provided by the authors and unedited

**Clinical Trial Protocol**

# Phase II study of regorafenib-nivolumab combination therapy for chemotherapy-naïve patients with unresectable or metastatic hepatocellular carcinoma

# RENOBATE Trial


**Principal Investigator**

Changhoon Yoo, MD, PhD

Assistant Professor, Department of Oncology, Asan Medical Center, Seoul, Korea

Tel: +82-2-3010-1727

E-mail: yooc@amc.seoul.kr


**Study Drug**: Nivolumab, regorafenib

**Protocol Number**:

**Version**:

**Date**:


**Confidentiality Statement**

Any and all information presented in this document shall be treated as confidential and shall remain the exclusive property of the parties mentioned above. The use of such confidential information must be restricted to the recipient for the agreed purpose and must not be disclosed, published, or otherwise communicated to any unauthorized persons, for any reason, in any form whatsoever without the prior written consent of the parties above

## PROTOCOL SYNOPSIS

**Study Title**

Phase II study of regorafenib-nivolumab combination therapy for chemotherapy-naïve patients with unresectable or metastatic hepatocellular carcinoma

**Protocol Number**

**Clinical Phase**

Phase 2

**Study Duration**

Patient enrollment for 11 months

Follow-up of last enrolled patients for 1 years

**Investigational Product(s)**

Nivolumab, Regorafenib

**Study Method**

Prospective study; interventional/clinical study; biomarker study

**Investigator/institution and contact person**

Changhoon Yoo, MD, Assistant Professor, Department of Oncology, Asan Medical Center, Seoul, Korea, Tel: +81-10-9900-6798, E-mail: yooc@amc.seoul.kr

**Objectives**

Primary Endpoints

- Objective response rates (proportion of complete response [CR] and PR) graded by Response Evaluation Criteria in Solid Tumors version 1.1 (RECIST 1.1)

Secondary Endpoints

- Safety profile of regorafenib-nivolumab combination graded by National Cancer Institute Common Terminology Criteria for Adverse Events version 5.0 (NCI-CTCAE v5.0)
- Progression-free survival (PFS) from the initiation of regorafenib-nivolumab combination
- Overall survival (OS) from the initiation of regorafenib-nivolumab combination

Exploratory Endpoints

- Correlative biomarker analysis using RNA sequencing, FACS analysis and circulating tumor DNA analysis

**Study Design:** Non-randomized, non-blinded, non-comparative, multi-center, open-label study

**Number of Centers**

3 centers in South Korea

**Number of Patients**

42 patients

**Study Population**

Chemotherapy naïve BCLC B or C stage unresectable hepatocellular carcinoma

**Inclusion Criteria**

1. Age ≥ 19 years at time of signing Informed Consent Form
2. Ability to comply with the study protocol, in the investigator's judgment
3. HCC that was histologically/cytologically confirmed or clinically diagnosed by AASLD criteria in cirrhotic patients. Patients without liver cirrhosis require histological confirmation of HCC
4. Locally advanced unresectable or metastatic disease that is not amenable to curative surgical and/or locoregional therapies, or that progressed after surgical and/or locoregional therapies
5. No prior systemic therapy for HCC
6. At least one measurable (per RECIST 1.1) lesion as confirmed by imaging within 28 days prior to initiation of study treatment.
7. Patients who received prior local therapy (e.g., radiofrequency ablation, percutaneous ethanol or acetic acid injection, cryoablation, high-intensity focused ultrasound, transarterial chemoembolization, transarterial embolization, etc.) are eligible provided that other target lesion(s) have not been previously treated with local therapy or the target lesion(s) within the field of local therapy have subsequently progressed in accordance with RECIST 1.1.
8. Pre-treatment tumor tissue sample (if available)
    - If tumor tissue is available, approximately 10–30 slides containing unstained, freshly cut, serial sections will be required subsequently for translational research.
    - If tumor tissue is not available (e.g., depleted because of prior diagnostic testing), patients are still eligible.
9. ECOG Performance Status score 0 or 1
10. Child-Pugh class A
11. Adequate hematologic and end-organ function, defined by the following laboratory test results, obtained within 14 days prior to initiation of study treatment, unless otherwise specified:
    - ANC ≥ 1.0 × $10^9$/L (1000/μL) without granulocyte colony-stimulating factor support
    - Platelet count ≥ 75 × $10^9$/L (75,000/μL) without transfusion
    - Hemoglobin ≥ 90 g/L (9 g/dL): Patients may be transfused to meet this criterion.
    - AST, ALT, and alkaline phosphatase (ALP) ≤ 3 × upper limit of normal (ULN)
    - Serum bilirubin ≤ 2 × ULN
    - Serum creatinine ≤ 1.5 × ULN or creatinine clearance ≥ 50 mL/min (calculated using the Cockcroft-Gault formula)
    - Serum albumin ≥ 28 g/L (2.8 g/dL)
    - For patients not receiving therapeutic anticoagulation: INR or aPTT ≤ 2× ULN
    - Urine dipstick for proteinuria < 2+
    - Patients discovered to have ≥ 2+ proteinuria on dipstick urinalysis at baseline should

undergo a 24-hour urine collection and must demonstrate < 1 g of protein in 24 hours.

12. Resolution of any acute, clinically significant treatment-related toxicity from prior therapy to Grade ≤ 1 prior to study entry, with the exception of alopecia
13. Negative HIV result at screening test or prior tested conducted within 3 years
14. Documented virology status of hepatitis, as confirmed by screening HBV and HCV serology test
    - Patients with active hepatitis B virus (HBV) must meet the followings: HBV DNA < 500 IU/mL obtained within 14 days prior to initiation of study treatment, anti-HBV treatment (per local standard of care; e.g., entecavir) for a minimum of 14 days prior to study entry and willingness to continue treatment for the length of the study
15. Women of childbearing potential (including women with chemical menopause or no menstruation for other medical reasons)[#1] must agree to use contraception[#2] from the time of informed consent until 5 months or more after the last dose of the investigational product. Also, women must agree not to breastfeed from the time of informed consent until 5 months or more after the last dose of the investigational product.
16. Men must agree to use contraception[#2] from the start of study treatment until 7 months or more after the last dose of the investigational product.

    #1. Women of childbearing potential are defined as all women after the onset of menstruation who are not postmenopausal and have not been surgically sterilized (e.g., hysterectomy, bilateral tubal ligation, bilateral oophorectomy). Postmenopause is defined as amenorrhea for ≥12 consecutive months without specific reasons. Women using oral contraceptives, intrauterine devices, or mechanical contraception such as contraceptive barriers are regarded as having childbearing potential.

    #2. The subject must consent to use any two of the following methods of contraception: vasectomy or condom for patients who are male or female subject's partner and tubal ligation, contraceptive diaphragm, intrauterine device, spermicide, or oral contraceptive for patients who are female or male subject's partner.

**Exclusion Criteria**

1. Patients who are diagnosed with fibrolamellar HCC, sarcomatoid HCC, or combined type of cholangiocarcinoma and HCC
2. Patients with a history of malignancy other than HCC within 3 years prior to screening, with the exception of malignancies with a negligible risk of metastasis or death (e.g., 5-year OS rate > 90%), such as adequately treated carcinoma in situ of the cervix, non-melanoma skin carcinoma, localized prostate cancer, ductal carcinoma in situ, and Stage I uterine cancer
3. Patients with a history of leptomeningeal seeding
4. Patients with symptomatic, untreated, or actively progressing central nervous system (CNS) metastases.
    - Asymptomatic patients with treated CNS lesions are eligible, provided that all of the following criteria are met:
        (1) The patients must have at least one measurable lesion, per RECIST 1.1, other than CNS metastases
        (2) The patient must not have a history of intracranial hemorrhage or spinal cord

hemorrhage

(3) The metastatic lesions have to be limited in cerebellum or supratentorial region (e.g., not to the midbrain, pons, medulla, or spinal cord)

(4) There must be no evidence of interim progression between the completion of CNS-directed therapy and initiation of the study treatment

(5) The patient must not undergo stereotactic radiotherapy within 7 days, whole-brain radiotherapy within 14 days, or neurosurgical resection within 28 days prior to initiation of the study treatment

(6) The patient must not have ongoing requirement for corticosteroids for CNS disease

- Anticonvulsant therapy at a stable dose is permitted.
- Asymptomatic patients with CNS metastases newly detected at screening are eligible for the study after receiving radiotherapy or surgery, with no need to repeat the screening brain scan.

5. Patients with current of past history of autoimmune disease or immunodeficient disease (including, but not limited to, myasthenia gravis, myositis, autoimmune hepatitis, systemic lupus erythematosus, rheumatoid arthritis, inflammatory bowel disease, antiphospholipid antibody syndrome, Wegener granulomatosis, Sjögren syndrome, Guillain-Barré syndrome, or multiple sclerosis) with the following exceptions:
   - Patients with autoimmune-related hypothyroidism who are on thyroid-replacement hormone are eligible.
   - Patients with controlled Type 1 diabetes mellitus who are on an insulin regimen are eligible.
   - Patients with eczema, psoriasis, lichen simplex chronicus, or vitiligo with dermatologic manifestations only (e.g., patients with psoriatic arthritis are excluded) are eligible for the study provided all of following conditions are met:

     (1) Rash must cover < 10% of body surface area

     (2) Disease has to be well controlled at baseline and requires only low-potency topical corticosteroids

     (3) There must be no occurrence of acute exacerbations of the underlying condition requiring psoralen plus ultraviolet A radiation, methotrexate, retinoids, biologic agents, oral calcineurin inhibitors, or high-potency or oral corticosteroids within the previous 12 months

6. Patients with current or past history of idiopathic pulmonary fibrosis, organizing pneumonia (e.g., bronchiolitis obliterans), drug-induced pneumonitis, or idiopathic pneumonitis, or evidence of active pneumonitis on screening chest computed tomography (CT) scan.
   - Patients with history of radiation pneumonitis in the radiation field (fibrosis) are eligible if the radiation pneumonitis has been confirmed as stable (beyond acute phase) without any concerns about recurrence.
7. Patients who have experienced a transient ischemic attack, cerebrovascular accident, thrombosis, or thromboembolism (pulmonary arterial embolism or deep vein thrombosis) within 6 months before initiation of study treatment
8. Patients with a history of uncontrollable or significant cardiovascular disease meeting any of the following criteria:
   - Myocardial infarction within 6 months before initiation of study treatment

- Uncontrollable angina pectoris within 6 months before initiation of study treatment
- New York Heart Association Class II or greater congestive heart failure within 6 months before initiation of study treatment
- Uncontrollable hypertension despite appropriate treatment (e.g., systolic blood pressure ≥150 mmHg or diastolic blood pressure > 90 mmHg based on an average of ≥ 3 BP readings on ≥ 2 sessions)
- Arrhythmia requiring treatment

9. Patients with congenital long QT syndrome or corrected QT interval >450 ms (calculated with use of the Fridericia method) at screening
10. Patients with systemic infections (including active tuberculosis) requiring treatment
11. Patients with history of hypertensive crisis or hypertensive encephalopathy
12. Patients with significant vascular disease (e.g., aortic aneurysm requiring surgical repair or recent peripheral arterial thrombosis) within 6 months prior to initiation of study treatment
13. Patients who underwent major surgical procedure, other than for diagnosis, within 4 weeks prior to initiation of study treatment or who are expected to need a major surgical procedure during the study
14. Patients who have received radiotherapy within 28 days before initiation, or radiotherapy to bone metastases within 14 days before initiation
15. Patients with prior history of allogeneic stem cell or solid organ transplantation
16. Patients with current or past history of severe allergic anaphylactic reactions to chimeric or humanized antibodies or fusion proteins
17. Patients with untreated or incompletely treated varices with active bleeding or high risk for bleeding
18. Patients with moderate or severe ascites
19. Patients with history of hepatic encephalopathy
20. Patients with evidence of bleeding diathesis or significant coagulopathy (in the absence of therapeutic anticoagulation)
21. Patients who had recent (within 10 days of first dose of study treatment) use of aspirin (> 300 mg/day) or treatment with dipyramidole, ticlopidine, clopidogrel, and cilostazol
22. Patients who had recent use of full-dose oral or parenteral anticoagulants or thrombolytic agents for therapeutic (as opposed to prophylactic) purpose
    - Prophylactic anticoagulation for the patency of venous access devices is allowed provided the activity of the agent results in an INR < 1.5 × ULN and aPTT within normal limits within 14 days prior to initiation of study treatment.
    - Prophylactic use of low molecular-weight heparin (i.e., enoxaparin 40 mg/day) is allowed.
23. Patients who treated with strong CYP3A4 inducers within 14 days prior to initiation of study treatment, including rifampin (and its analogues) or St. John's wort
24. Patients who have previously received CD137 agonists or immune checkpoint blockade therapies, including anti–CTLA-4, anti–PD-1, and anti–PD-L1 therapeutic antibodies
25. Patients who were treated with systemic immunostimulatory agents (including, but not limited to, interferon and interleukin 2 [IL-2]) within 4 weeks or 5 half-lives of the drug (whichever is

longer) prior to initiation of study treatment

26. Patients who were treated with systemic immunosuppressive medication (including, but not limited to, corticosteroids, cyclophosphamide, azathioprine, methotrexate, thalidomide, and anti–TNF-α agents) within 2 weeks prior to initiation of study treatment, or anticipation of need for systemic immunosuppressive medication during study treatment, with the following exceptions:
    - Patients who received temporary, low-dose systemic immunosuppressant medication or a one-time pulse dose of systemic immunosuppressant medication (e.g., 48 hours of corticosteroids for a contrast allergy) are eligible for the study.
    - Patients who received mineralocorticoids (e.g., fludrocortisone), or corticosteroids for chronic obstructive pulmonary disease (COPD) or asthma, or low-dose corticosteroids for orthostatic hypotension or adrenal insufficiency are eligible for the study.
27. Patients who had abdominal or tracheoesophageal fistula, gastrointestinal (GI) perforation, or intra-abdominal abscess within 6 months prior to initiation of study treatment
28. Patients who had intestinal obstruction and/or clinical signs or symptoms of GI obstruction including sub-occlusive disease related to the underlying disease or requirement for routine parenteral hydration, parenteral nutrition, or tube feeding within 6 months prior to initiation of study treatment
    - Patients with signs/symptoms of sub-/occlusive syndrome/intestinal obstruction at time of initial diagnosis may be enrolled if they had received definitive (surgical) treatment for symptom resolution.
29. Women who are pregnant or breastfeeding, or possibly pregnant
30. Other patients judged by the investigator or sub-investigator to be inappropriate as subjects of this study

**Treatment Flow**

**Single-arm phase 2 trial**
**Multicenter**

Target patient number:
Total 42 patients

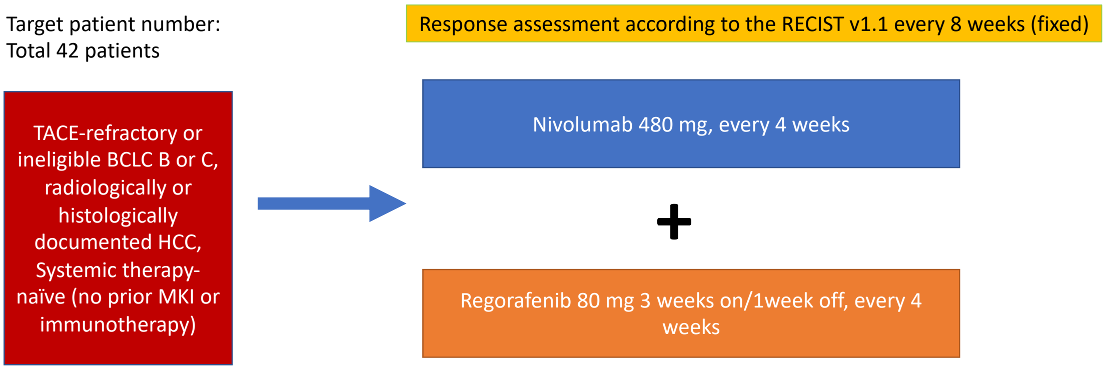


**Primary endpoint: ORR by RECIST v1.1**
**Secondary endpoint:**
**Safety profile, PFS, OS, ORR by mRECIST**

**Sample size estimation hypothesis:**
ORR of sorafenib (P0): 7%
ORR of Rego+Nivo (P1): 25%, two-sided alpha 0.05/beta 0.1
Fleming's single stage phase 2 design, drop-out rate 15%

- Fixed dose of nivolumab 480mg every 4 weeks
- Regorafenib: 80 mg QD 3 weeks on/1 week off, every 4 weeks.
- 4 weeks of study treatment is regarded as 1 cycle
- Response assessment will be performed every 8 weeks with RECIST 1.1.

Regorafenib-nivolumab combination therapy will be continued until the PD. Continuing regorafenib-nivolumab combination treatment beyond 1st PD will be allowed, if investigators assess that there is a potential clinical benefit.

**Dose of Investigational Products and Mode of Administration**

Nivolumab

- 480 mg IV on Day 1, every 4 weeks

Regorafenib

- 80 mg per oral once daily for 21 consecutive days starting on Day 1, every 4 weeks.
- Doses of regorafenib are allowed to be reduced to 40 mg according to the prespecified dose modification scheme.

**Study Assessments and Criteria for Evaluation**

Safety Assessments

Toxicity profiles including laboratory values and symptoms will be evaluated every cycle and graded according to NCI-CTCAE v5.0. Any SAEs will be reported to the Ethics Committee(s) and/or competent authorities, who will be responsible for submission within 24 hours. Patients will be reviewed up to 30 days after the last administration of study drug to document any late adverse effects.

Efficacy Assessments

Tumor assessment using CT and/or MRI will be performed every 8 weeks. Additional imaging can be done whenever progressive disease is clinically indicated. Response will be graded by RECIST 1.1.

**Exploratory Analysis**

Biomarker analysis using PBMC

- 20 ml of blood will be collected until 3 cycles (1 cycle=4 weeks): baseline, C1D15 (2 weeks), C2D1 (4 weeks), C3D1 (8 weeks)
- FACS and RNA sequencing analysis using blood samples before and after the study treatment
- Circulating tumor DNA analysis using baseline sample
- Correlative analyses with ORR, PFS, and OS will be done.

**Statistical Methods and Data Analysis:**

All dosed patients will be included for safety and efficacy analysis.

Patient characteristics and toxicity will be evaluated by descriptive methods. Chi-square and Fisher’s exact test will be used for comparing categorical variables. PFS and OS will be estimated

| |
|---|
| by Kaplan-Meier method and compared by log-rank test. |
| **Sample Size Determination:**<br>In the previous phase 3 trials, sorafenib, current standard 1st line therapy, showed the objective response rates (ORR) of 7% (P0) graded by RECIST v1.1. Regorafenib-nivolumab combination regimen might enhance the ORR to 25% (P1). With alpha of two sided 0.05 and power of 90%, 35 patients are needed based on Fleming's single-stage Phase 2 design calculation. Considering 15% of follow-up loss rates, a total 42 patients are needed for this study. |

# SCHEDULE OF STUDY ASSESSMENTS

| Trial Period | Screening | C1 D1[a] | C1 D15 | C2 D1 | C2 D15 | C3 D1 | C4 D1 ~ | Progression | For details see Section |
|---|---|---|---|---|---|---|---|---|---|
| Week | -4 to -1 | 0 | Q4W ±3 days unless dosing needs to be held for toxicity reasons | | | | | | |
| Day | -28 to -1 | 1 | Q28 days ±3 days unless dosing needs to be held for toxicity reasons | | | | | | |
| **Informed Consent** | | | | | | | | | |
| Informed consent: study procedures | X | | | | | | | | |
| Consent: tissue sample and biomarker analysis | X | | | | | | | | |
| **Study procedures** | | | | | | | | | |
| Medical history, demographics | X | | | | | | | | |
| Eligibility criteria | X | | | | | | | | |
| Physical exam | X | X | X | X | X | X | X | X | |
| Vital signs[a] | X | X | X | X | X | X | X | X | |
| ECG[b] | X | As clinically indicated | | | | | | | |
| Concomitant medications | <--------------------------------------------------------------------------------> | | | | | | | | |
| **Laboratory Assessments** | | | | | | | | | |
| Hematology[c] | X | X | X | X | X | X | X | X | |
| Clinical chemistry[c,d] | X | X | X | X | X | X | X | X | |
| Coagulation[c] | X | Clinically indicated | | | | | | | |

| Trial Period | Screening | C1 D1[a] | C1 D15 | C2 D1 | C2 D15 | C3 D1 | C4 D1 ~ | Progression | For details see Section |
|---|---|---|---|---|---|---|---|---|---|
| **Week** | **-4 to -1** | **0** | **Q4W ±3 days unless dosing needs to be held for toxicity reasons** | | | | | | |
| **Day** | **-28 to -1** | **1** | **Q28 days ±3 days unless dosing needs to be held for toxicity reasons** | | | | | | |
| TSH[g], free T4[e] | X | | Every 2 cycles (e.g., C3D1, C5D1…) | | | | | | |
| AFP, PIVKA-II[f] | X | Q8w ± 1w, then Q8w ± 1w, and whenever there is a finding suggestive of clinical deterioration or progressive disease. | | | | | | | |
| Hepatitis B and C[g], and HIV[h] | X | | | | | | | | |
| Urinalysis | X | | | | | | | | |
| Pregnancy test[i] | X | | | | | | | | |
| **Monitoring** | | | | | | | | | |
| ECOG performance status | X | X | X | X | X | X | X | X | |
| AE/SAE assessment[j] | <-------------------------------------------------------------------------------> | | | | | | | | |
| **IP administration** | | | | | | | | | |
| Nivolumab & Regorafenib | | X | | X | | X | X | | |
| **Other assessments and assays** | | | | | | | | | |
| Tissue sample[k] | X | | | | | | | | |
| Blood sample[l] | | X | X | X | | X | | | |
| **Efficacy evaluations** | | | | | | | | | |
| Tumor evaluation (RECIST 1.1)[m] | X | Q8w ± 1w, and whenever there is a finding suggestive of clinical deterioration or progressive disease. | | | | | | | |

[a] Body weight is recorded at each visit along with vital signs.

[b] Any clinically significant abnormalities detected require triplicate ECG results.

[c] If screening clinical chemistry, haematology, coagulation assessments are performed within 3 days prior to Day 1 (first infusion day), they do not need to be repeated at Day 1.

[d] Serum or plasma clinical chemistry (including LFT monitoring) and hematology may be performed more frequently if clinically indicated.

[e] If TSH, free T4 is measured within 14 days prior to Day 1 (first infusion day), it does not need to be repeated at day 1.

[f] Tumor markers (AFP and PIVKA-II) are followed up only in patients whose baseline tumor marker was higher than normal range.

[g] If a patient has hepatitis B infection, HBV DNA test should be performed.

[h] If a patient was negative for HIV test within 3 years, it does not need to be repeated.

[i] For women of childbearing potential only. A urine or serum pregnancy test is acceptable. Women of childbearing potential are required to have a pregnancy test within 7 days prior to the first dose of study drug and then every 4 weeks. Pregnancy test may occur on Day 1, but results must be available and reviewed by the treating physician or Investigator prior to commencing an infusion

[j] For AEs/SAEs reported during screening, additional information such as medical history and concomitant medications may be needed

[k] Up to 30 tissue slides from archival tissues from previous surgery or biopsy

[l] 20 ml of blood

[m] RECIST assessments will be performed on images from CT (preferred) or MRI, each preferably with IV contrast of the chest, abdomen (including liver and adrenal glands) and pelvis. Pelvic imaging is recommended only when primary or metastatic disease in the pelvic region is likely. Additional anatomy should be imaged based on signs and symptoms of individual patients at baseline and follow-up. Baseline assessments should be performed no more than 28 days before the start of the treatment and, ideally, should be performed as close as possible to and prior to the start of IP.

Note: All assessments on treatment days are to be performed prior to infusion, unless otherwise indicated.

# TABLE OF CONTENT

PROTOCOL SYNOPSIS ..... 2
SCHEDULE OF STUDY ASSESSMENTS ..... 10
TABLE OF CONTENT ..... 13
1.0 BACKGROUND ..... 16
1.1 Disease Background ..... 16
1.2 Nivolumab in HCC ..... 16
1.3 Regorafenib in HCC ..... 16
1.4 Research hypothesis and rationale ..... 16
2.0 OBJECTIVES ..... 18
2.1 Primary Objectives ..... 18
2.2 Secondary Objectives ..... 18
2.3 Exploratory Objectives ..... 18
3.0 STUDY DESIGN ..... 19
3.1 Study Scheme ..... 19
3.2 Endpoints ..... 19
3.2.1 Primary Endpoints ..... 19
3.2.2 Secondary Endpoints ..... 19
3.2.3 Exploratory Endpoints ..... 19
4.0 PATIENTS SELECTION ..... 20
4.1 Inclusion Criteria ..... 20
4.2 Exclusion Criteria ..... 21
4.3 Withdrawal Criteria ..... 24
4.3.1 Withdrawal of Consent ..... 25
5.0 INVESTIGATIONAL PRODUCTS ..... 26
5.1 Nivolumab ..... 26
5.1.1 Formulation/packaging/labelling/storage ..... 26
5.1.2 Dosage and treatment schedule ..... 26
5.1.3 Preparation and Administration ..... 26
5.2 Regorafenib ..... 27
5.2.1 Formulation/packaging/labelling/storage ..... 27
5.2.2 Dosage and treatment schedule ..... 27
5.2.3 Preparation and Administration ..... 27
5.3 Accountability procedures for the investigational products ..... 28
6.0 TREATMENT PLAN ..... 29
6.1 Dosing and Administration ..... 29
6.1.1 Treatment ..... 29

6.2 Dose modification ........ 29
6.2.1 Nivolumab ........ 29
6.2.2 Regorafenib ........ 31
6.3 Toxicity Management ........ 32
6.3.1 Nivolumab ........ 32
6.3.2 Regorafenib ........ 37
6.4 Duration of Treatment ........ 38
6.5 Discontinuation of Treatment ........ 38
6.6 Concomitant Treatment ........ 39
6.6.1 Prohibited treatment ........ 39
6.6.2 Permitted treatment ........ 40
Monitoring compliance ........ 41
7.0 STUDY PROCEDURE ........ 42
7.1 Schedule of study procedures ........ 42
7.1.1 Screening phase ........ 42
7.1.2 Treatment phase ........ 42
7.1.3 End of treatment ........ 43
7.2 Description of study procedures ........ 43
7.2.1 Medical history and physical examination, electrocardiogram, weight, and vital signs ........ 43
7.2.2 Clinical laboratory tests ........ 44
7.3 Biological Sampling Procedures ........ 46
7.3.1 Sample collection ........ 46
7.3.2 Exploratory analysis ........ 46
7.3.3 Withdrawal of informed consent for donated biological samples ........ 46
8.0 ASSESSMENT OF EFFICACY ........ 47
9.0 ASSESSMENT OF SAFETY ........ 48
9.1 Definition of safety parameters. ........ 48
9.1.1 Adverse Event ........ 48
9.1.2 Serious Adverse Event ........ 48
9.2 Grading ........ 48
9.3 Relationship to Drug ........ 49
9.4 Recording Safety Parameters ........ 49
9.4.1 Recording period and follow-up for safety parameters ........ 49
9.4.2 Adverse events based on signs and symptoms ........ 50
9.4.3 Adverse events based on examinations and tests ........ 50
9.4.4 Preexisting Medical Conditions (Baseline Conditions) ........ 51
9.4.5 New cancers ........ 51
9.4.6 Death ........ 51

9.5 Other events requiring reporting ........ 52
9.5.1 Pregnancy ........ 52
10.0 STATISTICS ........ 52
10.1 Description of analysis sets ........ 52
10.1.1 Safety analysis set ........ 52
10.1.2 Efficacy analysis set ........ 52
10.2 Methods of statistical analyses ........ 52
10.2.1 Safety analysis ........ 52
10.2.2 Efficacy analysis ........ 53
10.2.3 Exploratory analyses ........ 53
10.3 Determination of sample size ........ 54
11.0 STUDY MANAGEMENT ........ 55
11.1 Training of study site personnel ........ 55
11.2 Monitoring of the study ........ 55
11.3 Timelines ........ 55
12. ETHICAL AND REGULATORY REQUIREMENTS ........ 56
12.1 Ethical conduct of the study ........ 56
12.2 Informed consent ........ 56
12.3 Changes to the protocol and informed consent form ........ 56
12.4 Audits and inspections ........ 56
12.5 Confidentiality ........ 56
12.5.1 Confidentiality of Data ........ 56
12.5.1 Confidentiality of Subject Records ........ 57
13.0 DATA MANAGEMENT ........ 57
APPENDIX ........ 58
Appendix 1. ECOG Performance Status Scores ........ 58
Appendix 2. Common Terminology Criteria for Adverse Events V5.0 (CTCAE) ........ 58
Appendix 3. Response evaluation criteria in solid tumors (RECIST) version 1.1 ........ 58
Appendix 4. Schedule of Study Procedures: Follow-Up for Patients Who Have Discontinued Study Treatment Due to Progression of Disease or unacceptable toxicities ........ 58

# 1.0 BACKGROUND

## 1.1 Disease Background

Hepatocellular carcinoma (HCC) is the most common primary malignancy of liver, with more than 700,000 new cases being diagnosed each year throughout the world. Many loco-regional therapeutic modalities including radiofrequency ablation (RFA), or percutaneous ethanol injection (PEI), and trans-arterial chemoembolization (TACE) play important roles in treating patients with early stage HCC, as well as liver transplantation or surgical resection, which are potentially curative treatment options for Barcelona Clinic Liver Cancer (BCLC) stage 0 or A HCC. However, some patients have advanced disease at the time of diagnosis, and tumor recurrence or progression is common even after the local directed therapies. Although VEGFR-targeted multikinase inhibitors (MKIs) including sorafenib, regorafenib and lenvatinib have proven their efficacy in unresectable HCC and been established as the standard of care, prognosis of these patients remains poor, with a median survival around 1 year. Recently, development of immune checkpoint inhibitors is expected to change the treatment paradigm of unresectable HCC, and currently under large randomized trials.

## 1.2 Nivolumab in HCC

Nivolumab (BMS-936558) is a fully human monoclonal immunoglobulin (Ig) G4 antibody that binds to the PD-1 cell surface membrane receptor, a negative regulatory molecule expressed by activated T and B lymphocytes. Inhibition of the interaction between PD-1 and its ligands promote immune responses and antigen-specific T cell responses to both foreign and self-antigens. Results from a Phase I/II study (CA209003/MDX1106-03) indicate that nivolumab is active in multiple tumor types.

In the Checkmate-040 study, nivolumab has shown the promising efficacy as 1st line therapy or 2nd line therapy after failure of sorafenib. In sorafenib-naïve patients, response rates were 20% and median OS was 28.6 months with nivolumab. However, in Checkmate-459 phase III trial, nivolumab monotherapy failed to significantly improve overall survival of patients with advanced HCC compared with sorafenib.

## 1.3 Regorafenib in HCC

Regorafenib is a a small molecule inhibitor of multiple membrane-bound and intracellular kinases involved in normal cellular functions and in pathologic processes such as oncogenesis, tumor angiogenesis, and maintenance of the tumor microenvironment. In in vitro biochemical or cellular assays, regorafenib or its major human active metabolites M-2 and M-5 inhibited the activity of RET, VEGFR1, VEGFR2, VEGFR3, KIT, PDGFR-alpha, PDGFR-beta, FGFR1, FGFR2, TIE2, DDR2, Trk2A, Eph2A, RAF-1, BRAF, BRAFV600E, SAPK2, PTK5, and Abl at concentrations of regorafenib that have been achieved clinically. Regorafenib improved OS of HCC patients who progressed on sorafenib (median 10.6 months for regorafenib versus 7.8 months for placebo) with the response rate of 7% in RESORCE trial. Regorafenib was the first treatment that proved efficacy in sorafenib-failed HCC patients and approved as 2$^{nd}$ line treatment for HCC in multiple countries including United States and Korea.

## 1.4 Research hypothesis and rationale

Combination of regorafenib and nivolumab may be synergistic in terms of efficacy considering that both agents are active and have proven their efficacy in the trials for patients with unresectable HCC.

Furthermore, regorafenib has shown potential immune modulation impact as follows, and therefore, the combination therapy of regorafenib with nivolumab may show enhanced efficacy in unresectable HCC patients. In prior phase 1 dose escalation trial for the combination between regorafenib and nivolumab, 80 mg of regorafenib and standard dose of nivolumab was well tolerated in advanced gastric and colorectal cancer patients and showed promising efficacy.

- Colony-stimulating factor 1 receptor (CSF1R) inhibition by regorafenib

Tumor-associated macrophages (TAM) are known to be one of the critical drivers of immune escape in the tumor microenvironment. There has been a report that blockade of colony-stimulating factor 1 receptor (CSF1R) results in improved anti-tumor T cell responses, suggesting CSF1R blockade could be effective at alleviating local tumor-induced immune suppression and bolstering the response to immunotherapy. Preclinical data showed that blockade of CSF1/CSF1R signaling improved the efficacy of anti-PDL1 and anti-CTLA4 immunotherapy in pancreas cancer cell lines.
Regorafenib blocks multiple protein kinases including CSF1R, potential therapeutic target to reprogram the immunosuppressive microenvironment and enhance the efficacy of immunotherapy.

- Immune modulating effects by regorafenib

Our in-house data from Asan Medical Center using multiplexed immunohistochemistry revealed that multikinase inhibitors including sunitinib or regorafenib enhanced the infiltration of TILs and PD-1 expression. This indicates that regorafenib may modulate immune microenvironment improving the efficacy of immune checkpoint inhibitors, such as anti-PD-1 inhibitor.

At present, various combinations of MKIs and immune checkpoint inhibitors are ongoing as 1$^{st}$-line therapy for patients with unresectable HCC based on the promising efficacy results (response rates ranged between 30% and 40%) in early phase 1 trials for atezolizumab plus bevacizumab and lenvatinib plus pembrolizumab. Combination regorafenib-nivolumab for patients may result in greater efficacy.

# 2.0 OBJECTIVES

## 2.1 Primary Objectives

To evaluate the objective response rates (defined by RECIST v1.1) of regorafenib-nivolumab combination in unresectable HCC patients

## 2.2 Secondary Objectives

To evaluate PFS, OS, safety profile and modified RECIST with regorafenib-nivolumab combination in patients with unresectable HCC

## 2.3 Exploratory Objectives

To identify predictive biomarkers of regorafenib-nivolumab combination for clinical outcomes

To identify impact of regorafenib-nivolumab on immune microenvironment

# 3.0 STUDY DESIGN

## 3.1 Study Scheme

This study is a phase II, open-label, non-blinding, non-comparative, multicenter trial for patients with advanced hepatocellular carcinoma.

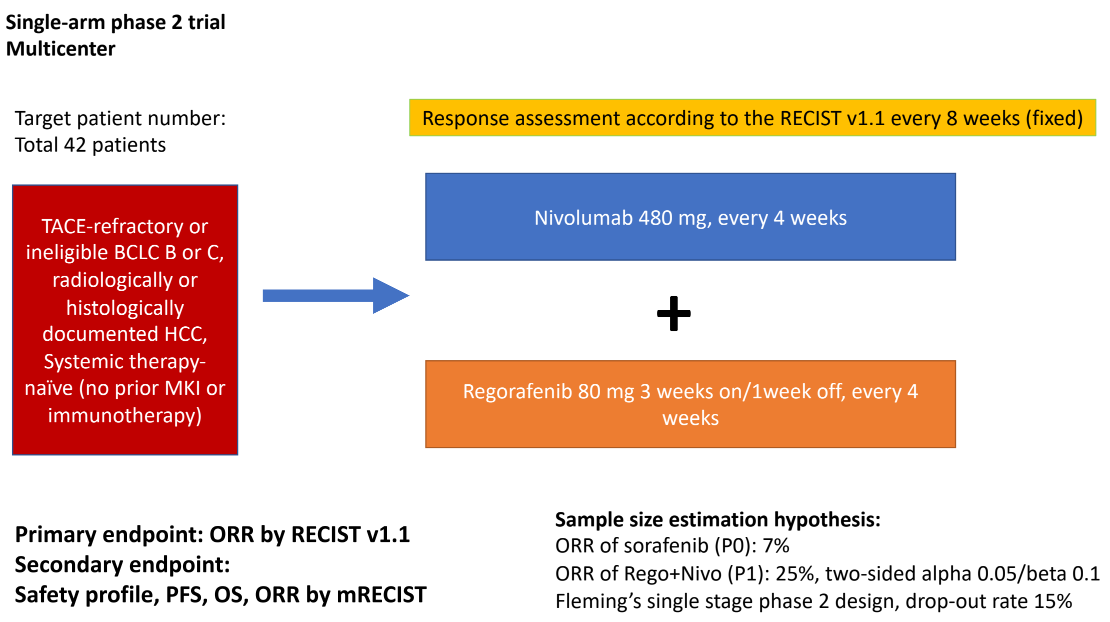


All eligible patients for this study will receive nivolumab 480mg on day 1 and regorafenib 80mg QD on day 1-21 every 4 weeks. Response assessment will be performed every 8 weeks with RECIST 1.1.

## 3.2 Endpoints

### 3.2.1 Primary Endpoints

- Objective response rates (proportion of complete response [CR] and PR) graded by RECIST 1.1

### 3.2.2 Secondary Endpoints

- Safety profile of regorafenib-nivolumab combination graded by NCI-CTCAE v5.0
- PFS from the initiation of regorafenib-nivolumab combination
- OS from the initiation of regorafenib-nivolumab combination
- Objective response rates graded by mRECIST

### 3.2.3 Exploratory Endpoints

- Correlative biomarker analysis using RNA sequencing, FACS analysis and circulating tumor DNA analysis

# 4.0 PATIENTS SELECTION

## 4.1 Inclusion Criteria

Having provided written consent before participation in the study, patients must fulfill all of the following criteria:

1. Age ≥ 19 years at time of signing Informed Consent Form
2. Ability to comply with the study protocol, in the investigator's judgment
3. HCC that was histologically/cytologically confirmed or clinically diagnosed by AASLD criteria in cirrhotic patients. Patients without liver cirrhosis require histological confirmation of HCC
4. Locally advanced unresectable or metastatic disease that is not amenable to curative surgical and/or locoregional therapies, or that progressed after surgical and/or locoregional therapies
5. No prior systemic therapy for HCC
6. At least one measurable (per RECIST 1.1) lesion as confirmed by imaging within 28 days prior to initiation of study treatment
7. Patients who received prior local therapy (e.g., radiofrequency ablation, percutaneous ethanol or acetic acid injection, cryoablation, high-intensity focused ultrasound, transarterial chemoembolization, transarterial embolization, etc.) are eligible provided that other target lesion(s) have not been previously treated with local therapy or the target lesion(s) within the field of local therapy have subsequently progressed in accordance with RECIST 1.1.
8. Pre-treatment tumor tissue sample (if available)
    - If tumor tissue is available, approximately 10–30 slides containing unstained, freshly cut, serial sections will be required subsequently for translational research.
    - If tumor tissue is not available (e.g., depleted because of prior diagnostic testing), patients are still eligible.
9. ECOG Performance Status score 0 or 1
10. Child-Pugh class A
11. Adequate hematologic and end-organ function, defined by the following laboratory test results, obtained within 14 days prior to initiation of study treatment, unless otherwise specified:
    - ANC ≥ 1.0 × $10^9$/L (1000/μL) without granulocyte colony-stimulating factor support
    - Platelet count ≥ 75 × $10^9$/L (75,000/μL) without transfusion
    - Hemoglobin ≥ 90 g/L (9 g/dL): Patients may be transfused to meet this criterion.
    - AST, ALT, and alkaline phosphatase (ALP) ≤ 3 × upper limit of normal (ULN)
    - Serum bilirubin ≤ 2× ULN
    - Serum creatinine ≤ 1.5 × ULN or creatinine clearance ≥ 50 mL/min (calculated using the Cockcroft-Gault formula)
    - Serum albumin ≥ 28 g/L (2.8 g/dL)
    - For patients not receiving therapeutic anticoagulation: INR or aPTT ≤ 2× ULN
    - Urine dipstick for proteinuria < 2+
    - Patients discovered to have ≥ 2+ proteinuria on dipstick urinalysis at baseline should

undergo a 24-hour urine collection and must demonstrate < 1 g of protein in 24 hours.

12. Resolution of any acute, clinically significant treatment-related toxicity from prior therapy to Grade ≤ 1 prior to study entry, with the exception of alopecia
13. Negative HIV result at screening test or prior tested conducted within 3 years
14. Documented virology status of hepatitis, as confirmed by screening HBV and HCV serology test
    - Patients with active hepatitis B virus (HBV) must meet the followings: HBV DNA < 500 IU/mL obtained within 14 days prior to initiation of study treatment, anti-HBV treatment (per local standard of care; e.g., entecavir) for a minimum of 14 days prior to study entry and willingness to continue treatment for the length of the study
15. Women of childbearing potential (including women with chemical menopause or no menstruation for other medical reasons)[#1] must agree to use contraception[#2] from the time of informed consent until 5 months or more after the last dose of the investigational product. Also, women must agree not to breastfeed from the time of informed consent until 5 months or more after the last dose of the investigational product.
16. Men must agree to use contraception[#2] from the start of study treatment until 7 months or more after the last done of the investigational product.

    #1 Women of childbearing potential are defined as all women after the onset of menstruation who are not postmenopausal and have not been surgically sterilized (e.g., hysterectomy, bilateral tubal ligation, bilateral oophorectomy). Postmenopause is defined as amenorrhea for ≥12 consecutive months without specific reasons. Women using oral contraceptives, intrauterine devices, or mechanical contraception such as contraceptive barriers are regarded as having childbearing potential.

    #2 The subject must consent to use any two of the following methods of contraception: vasectomy or condom for patients who are male or female subject's partner and tubal ligation, contraceptive diaphragm, intrauterine device, spermicide, or oral contraceptive for patients who are female or male subject's partner.

## 4.2 Exclusion Criteria

Patients who meet any of the following criteria at the time of screening will be excluded. If a subject is found to meet any of the following criteria before the first dose of the investigational product, the subject will not be started on the study treatment and will be withdrawn from the study.

1. Patients who are diagnosed with fibrolamellar HCC, sarcomatoid HCC, or combined type of cholangiocarcinoma and HCC
2. Patients with a history of malignancy other than HCC within 3 years prior to screening, with the exception of malignancies with a negligible risk of metastasis or death (e.g., 5-year OS rate > 90%), such as adequately treated carcinoma in situ of the cervix, non-melanoma skin carcinoma, localized prostate cancer, ductal carcinoma in situ, and Stage I uterine cancer
3. Patients with a history of leptomeningeal seeding
4. Patients with symptomatic, untreated, or actively progressing central nervous system (CNS) metastases.
    - Asymptomatic patients with treated CNS lesions are eligible, provided that all of the

following criteria are met:

(4) The patients must have at least one measurable lesion, per RECIST 1.1, other than CNS metastases

(5) The patient must not have a history of intracranial hemorrhage or spinal cord hemorrhage

(6) The metastatic lesions have to be limited in cerebellum or supratentorial region (e.g., not to the midbrain, pons, medulla, or spinal cord)

(7) There must be no evidence of interim progression between the completion of CNS-directed therapy and initiation of the study treatment

(8) The patient must not undergo stereotactic radiotherapy within 7 days, whole-brain radiotherapy within 14 days, or neurosurgical resection within 28 days prior to initiation of the study treatment

(9) The patient must not have ongoing requirement for corticosteroids for CNS disease

- Anticonvulsant therapy at a stable dose is permitted.
- Asymptomatic patients with CNS metastases newly detected at screening are eligible for the study after receiving radiotherapy or surgery, with no need to repeat the screening brain scan.

5. Patients with current of past history of autoimmune disease or immunodeficient disease (including, but not limited to, myasthenia gravis, myositis, autoimmune hepatitis, systemic lupus erythematosus, rheumatoid arthritis, inflammatory bowel disease, antiphospholipid antibody syndrome, Wegener granulomatosis, Sjögren syndrome, Guillain-Barré syndrome, or multiple sclerosis) with the following exceptions:
   - Patients with autoimmune-related hypothyroidism who are on thyroid-replacement hormone are eligible.
   - Patients with controlled Type 1 diabetes mellitus who are on an insulin regimen are eligible.
   - Patients with eczema, psoriasis, lichen simplex chronicus, or vitiligo with dermatologic manifestations only (e.g., patients with psoriatic arthritis are excluded) are eligible for the study provided all of following conditions are met:

   (10) Rash must cover < 10% of body surface area

   (11) Disease has to be well controlled at baseline and requires only low-potency topical corticosteroids

   (12) There must be no occurrence of acute exacerbations of the underlying condition requiring psoralen plus ultraviolet A radiation, methotrexate, retinoids, biologic agents, oral calcineurin inhibitors, or high-potency or oral corticosteroids within the previous 12 months

*6.* Patients with current or past history of idiopathic pulmonary fibrosis, organizing pneumonia (e.g., bronchiolitis obliterans), drug-induced pneumonitis, or idiopathic pneumonitis, or evidence of active pneumonitis on screening chest computed tomography (CT) scan.
   - Patients with history of radiation pneumonitis in the radiation field (fibrosis) are eligible if the radiation pneumonitis has been confirmed as stable (beyond acute phase) without any concerns about recurrence.

7. Patients who have experienced a transient ischemic attack, cerebrovascular accident,

thrombosis, or thromboembolism (pulmonary arterial embolism or deep vein thrombosis) within 6 months before initiation of study treatment

8. Patients with a history of uncontrollable or significant cardiovascular disease meeting any of the following criteria:
   - Myocardial infarction within 6 months before initiation of study treatment
   - Uncontrollable angina pectoris within 6 months before initiation of study treatment
   - New York Heart Association Class II or greater congestive heart failure within 6 months before initiation of study treatment
   - Uncontrollable hypertension despite appropriate treatment (e.g., systolic blood pressure ≥150 mmHg or diastolic blood pressure > 90 mmHg based on an average of ≥ 3 BP readings on ≥ 2 sessions)
   - Arrhythmia requiring treatment
9. Patients with congenital long QT syndrome or corrected QT interval > 450 ms (calculated with use of the Fridericia method) at screening
10. Patients with systemic infections (including active tuberculosis) requiring treatment
11. Patients with history of hypertensive crisis or hypertensive encephalopathy
12. Patients with significant vascular disease (e.g., aortic aneurysm requiring surgical repair or recent peripheral arterial thrombosis) within 6 months prior to initiation of study treatment
13. Patients who underwent major surgical procedure, other than for diagnosis, within 4 weeks prior to initiation of study treatment or who are expected to need a major surgical procedure during the study
14. Patients who have received radiotherapy within 28 days before initiation, or radiotherapy to bone metastases within 14 days before initiation
15. Patients with prior history of allogeneic stem cell or solid organ transplantation
16. Patients with current or past history of severe allergic anaphylactic reactions to chimeric or humanized antibodies or fusion proteins
17. Patients with untreated or incompletely treated varices with active bleeding or high risk for bleeding
18. Patients with moderate or severe ascites
19. Patients with history of hepatic encephalopathy
20. Patients with evidence of bleeding diathesis or significant coagulopathy (in the absence of therapeutic anticoagulation)
21. Patients who had recent (within 10 days of first dose of study treatment) use of aspirin (> 300 mg/day) or treatment with dipyramidole, ticlopidine, clopidogrel, and cilostazol
22. Patients who had recent use of full-dose oral or parenteral anticoagulants or thrombolytic agents for therapeutic (as opposed to prophylactic) purpose
   - Prophylactic anticoagulation for the patency of venous access devices is allowed provided the activity of the agent results in an INR < 1.5 × ULN and aPTT within normal limits within 14 days prior to initiation of study treatment.
   - Prophylactic use of low molecular-weight heparin (i.e., enoxaparin 40 mg/day) is allowed.
23. Patients who treated with strong CYP3A4 inducers within 14 days prior to initiation of study

treatment, including rifampin (and its analogues) or St. John's wort

24. Patients who have previously received CD137 agonists or immune checkpoint blockade therapies, including anti–CTLA-4, anti–PD-1, and anti–PD-L1 therapeutic antibodies
25. Patients who were treated with systemic immunostimulatory agents (including, but not limited to, interferon and interleukin 2 [IL-2]) within 4 weeks or 5 half-lives of the drug (whichever is longer) prior to initiation of study treatment
26. Patients who were treated with systemic immunosuppressive medication (including, but not limited to, corticosteroids, cyclophosphamide, azathioprine, methotrexate, thalidomide, and anti–TNF-α agents) within 2 weeks prior to initiation of study treatment, or anticipation of need for systemic immunosuppressive medication during study treatment, with the following exceptions:
    - Patients who received temporary, low-dose systemic immunosuppressant medication or a one-time pulse dose of systemic immunosuppressant medication (e.g., 48 hours of corticosteroids for a contrast allergy) are eligible for the study.
    - Patients who received mineralocorticoids (e.g., fludrocortisone), or corticosteroids for chronic obstructive pulmonary disease (COPD) or asthma, or low-dose corticosteroids for orthostatic hypotension or adrenal insufficiency are eligible for the study.
27. Patients who had abdominal or tracheoesophageal fistula, gastrointestinal (GI) perforation, or intra-abdominal abscess within 6 months prior to initiation of study treatment
28. Patients who had intestinal obstruction and/or clinical signs or symptoms of GI obstruction including sub-occlusive disease related to the underlying disease or requirement for routine parenteral hydration, parenteral nutrition, or tube feeding within 6 months prior to initiation of study treatment
    - Patients with signs/symptoms of sub-/occlusive syndrome/intestinal obstruction at time of initial diagnosis may be enrolled if they had received definitive (surgical) treatment for symptom resolution.
29. Women who are pregnant or breastfeeding, or possibly pregnant
30. Other patients judged by the investigator or sub-investigator to be inappropriate as subjects of this study
31. Patients with a history of hypersensitivity to the components of Nivolumab
32. Both hepatitis B and C as evidenced by detectable HBV surface antigen (HBs Ag) or HBV DNA and HCV RNA.
33. Hepatitis D infection in participants with hepatitis B.

## 4.3 Withdrawal Criteria

An individual patient will not receive any further investigational product if any of the following occur in the patient in question:

1. Withdrawal of consent or lost to follow-up
2. Patient is determined to have met one or more of the exclusion criteria for study participation at study entry and continuing investigational therapy might constitute a safety risk
3. Adverse event that meets criteria for discontinuation as defined in Section 6.2.1.4 or 6.2.2.3, or in the opinion of the investigator or the sponsor, contraindicates further dosing

4. Progressive disease and investigator determination that the patient is no longer benefiting from the study treatment
5. Non-compliance with the study protocol that, in the opinion of the investigator or sponsor, warrants withdrawal from treatment with IP (e.g., refusal to adhere to scheduled visits)
6. Pregnancy or intent to become pregnant
7. Initiation of alternative anticancer therapy including another investigational agent

Patients who are permanently discontinued from receiving investigational product will be followed for survival and safety assessment per Section 9.4.1, unless consent is withdrawn. Patients who decline to return to the site for evaluations will be offered follow-up by phone every 3 months as an alternative.

### 4.3.1 Withdrawal of Consent

Patients are free to withdraw from the study at any time (IP and assessments) without prejudice to further treatment.

A patient who withdraws consent will be asked about the reasons for withdrawal and the presence of any AE. Investigator will follow up and mange AEs outside of the clinical study.

If a patient withdraws consent, they will be specifically asked if they are withdrawing consent to:

1. All further participation in the study including any further follow up (e.g., survival contact telephone calls)
2. Withdrawal of consent to the use of their study generated data
3. Withdrawal to the use of any samples

# 5.0 INVESTIGATIONAL PRODUCTS

## 5.1 Nivolumab

### 5.1.1 Formulation/packaging/labelling/storage

*Formulation and packaging*

Nivolumab (OPDIVO®) will be provided by ONO Pharma as 100 mg/10 mL (10 mg/mL) clear to opalescent, colorless to pale-yellow solution in a single-dose vial.

*Labelling*

Clinical supplies will be affixed with a clinical label in accordance with regulatory requirements. This trial is open-label; therefore, the subject, the trial site personnel, designee are not blinded to treatment. Drug identity (name, strength) is included in the label text.

*Storage*

Clinical supplies must be stored in a secure, limited-access location under the storage conditions specified on the label. Receipt and dispensing of trial medication must be recorded by an authorized person at the trial site. Clinical supplies may not be used for any purpose other than that stated in the protocol.

### 5.1.2 Dosage and treatment schedule

Nivolumab 480 mg IV on Day 1, every 4 weeks

### 5.1.3 Preparation and Administration

Visually inspect drug product solution for particulate matter and discoloration prior to administration. OPDIVO is a clear to opalescent, colorless to pale-yellow solution. Discard the vial if the solution is cloudy, discolored, or contains extraneous particulate matter other than a few translucent-to-white, proteinaceous particles. Do not shake the vial.

*Preparation*

1. Withdraw the required volume of nivolumab and transfer into an intravenous container.
2. Dilute nivolumab with either 0.9% Sodium Chloride Injection or 5% Dextrose Injection to prepare an infusion with a final concentration ranging from 1 mg/mL to 10 mg/mL. The total volume of infusion must not exceed 160 mL.
3. Mix diluted solution by gentle inversion. Do not shake.
4. Discard partially used vials or empty vials of nivolumab

*Storage of Infusion*

The product does not contain a preservative.

After preparation, store the nivolumab infusion either:

1. at room temperature for no more than 8 hours from the time of preparation. This includes room temperature storage of the infusion in the IV container and time for administration of the

infusion or

2. under refrigeration at 2°C to 8°C (36°F to 46°F) for no more than 24 hours from the time of infusion preparation.
3. Do not freeze.

*Administration*

1. Administer the infusion over 30 minutes through an intravenous line containing a sterile, non-pyrogenic, low protein binding in-line filter (pore size of 0.2 micrometer to 1.2 micrometer).
2. Do not co-administer other drugs through the same intravenous line.
3. Flush the intravenous line at end of infusion.

## 5.2 Regorafenib

### 5.2.1 Formulation/packaging/labelling/storage

*Formulation and packaging*

Regorafenib (Stivarga®) will be provided by Bayer. Regorafenib is supplied as a 40 mg, light pink, oval shaped, film-coated tablet, debossed with 'BAYER' on one side and '40' on the other side. Regorafenib tablets are supplied in packages containing three bottles, with each bottle containing 28 tablets, for a total of 84 tablets per package.

*Labelling*

Clinical supplies will be affixed with a clinical label in accordance with regulatory requirements. This trial is open-label; therefore, the subject, the trial site personnel, designee are not blinded to treatment. Drug identity (name, strength) is included in the label text.

*Storage*

Store the drug bottle between 15 and 30°C.

Store tablets in the original bottle and do not remove the desiccant.

Keep the bottle tightly closed after first opening.

Discard any unused tablets 28 days after opening the bottle.

Dispose of unused tablets in accordance with local requirements.

### 5.2.2 Dosage and treatment schedule

Regorafenib 80 mg per oral once daily for 21 consecutive days starting on Day 1, every 4 weeks. Doses of regorafenib for combination with nivolumab are allowed to be reduced to 40 mg according to the prespecified dose modification scheme (Section 6.2.2).

### 5.2.3 Preparation and Administration

1. Take regorafenib at the same time each day.
2. Swallow tablet whole with a low-fat breakfast that contains less than 30% fat. Examples of a low-fat breakfast include 2 slices of white toast with 1 tablespoon of low-fat margarine and 1

tablespoon of jelly, and 8 ounces of skim milk (319 calories and 8.2 g fat); or 1 cup of cereal, 8 ounces of skim milk, 1 slice of toast with jam, apple juice, and 1 cup of coffee or tea (520 calories and 2 g fat).

3. Take any missed dose on the same day, as soon as they remember, and that they must not take two doses on the same day to make up for a dose missed on the previous day.
4. Store medicine in the original container. Do not place medication in daily or weekly pill boxes. Any remaining tablets should be discarded 28 days after opening the bottle. Tightly close bottle after each opening and keep the desiccant in the bottle.

## 5.3 Accountability procedures for the investigational products

The investigator shall take responsibility for and shall take all steps to maintain appropriate records and ensure appropriate supply, storage, handling, distribution and usage of investigational product in accordance with the protocol and any applicable laws and regulations.

# 6.0 TREATMENT PLAN

## 6.1 Dosing and Administration

### 6.1.1 Treatment

The starting dose of regorafenib for combination with nivolumab is 80mg (two 40-mg tablets) orally once daily for three weeks followed by one week off to comprise a 4-week schedule.

Nivolumab 480 mg will be given via IV infusion every 4 weeks (Day 1 of every 4-week cycle).

Regorafenib-nivolumab combination will be continued until progression. Continuing treatment beyond 1$^{st}$ PD will be allowed, if investigators assess that there is a potential clinical benefit.

## 6.2 Dose modification

### 6.2.1 Nivolumab

#### 6.2.1.1. Dose reduction

No dose reduction for nivolumab is allowed.

#### 6.2.1.2 Dose delay criteria

Nivolumab should be delayed for the following:

1. Grade 3 skin rash or suspected Stevens-Johnson syndrome or toxic epidermal necrolysis
2. Any Grade ≥ 2 non-skin drug-related adverse event, with the exceptions of 2 drug-related fatigue or laboratory abnormalities
3. Any Grade ≥ 3 drug-related laboratory abnormality with the following exceptions of Grade 3 amylase or lipase abnormalities that are not associated with symptoms or clinical manifestations of pancreatitis
4. Dose delay for changes in AST or ALT as follows:
   - If a subject has a baseline AST or ALT that is within normal limits, delay dosing for drug-related Grade ≥ 2 toxicity (2 grade shift)
   - If a subject has baseline AST or ALT within the Grade 1 toxicity range, delay dosing for drug-related Grade ≥ 3 toxicity (2 grade shift)
   - If a subject has baseline AST or ALT within the Grade 2 toxicity range, delay dosing for a two-fold drug-related increase in AST or ALT or for AST or ALT values 8x ULN (whichever is lower).
5. Any adverse event, laboratory abnormality, or intercurrent illness which, in the judgment of the investigator, warrants delaying the dose of study medication.
6. Encephalitis: New-onset moderate or severe neurologic signs or symptoms

Subjects who require delay of nivolumab should be re-evaluated weekly or more frequently if clinically indicated. It is recommended to monitor elevations in AST or ALT approximately every 3 days till levels

peak or begin to decline. Nivolumab dosing can be resumed when re-treatment criteria are met (Section 6.2.1.3). Tumor assessments for all subjects should continue as per protocol even if dosing is delayed.

#### 6.2.1.3 Criteria to resume dosing for nivolumab

Subjects may resume treatment with study drug when the drug-related AE(s) resolve to Grade ≤ 1 or baseline value, with the following exceptions:

1. Subjects may resume treatment in the presence of Grade 2 fatigue.
2. Subjects who have not experienced a Grade 3 drug-related skin AE may resume treatment in the presence of Grade 2 skin toxicity.
3. Subjects with baseline Grade 1 AST, ALT, or total bilirubin who require dose delays for reasons other than a drug-related hepatic event may resume treatment in the presence of Grade 2 AST, ALT, or total bilirubin.
4. Subjects who require dose delays for drug-related elevations in AST, ALT, or total bilirubin may resume treatment when these values have returned to their baseline CTCAE Grade or normal, provided the criteria for permanent discontinuation are not met.
5. Drug-related pulmonary toxicity, diarrhea, or colitis must have resolved to baseline before treatment is resumed
6. Drug-related endocrinopathies adequately controlled with only physiologic hormone replacement may resume treatment

If the criteria to resume treatment are met, the subject should restart treatment at the next scheduled timepoint per protocol. If treatment is delayed > 6 weeks from the last dose, the subject must be permanently discontinued from study therapy, except as specified in section 6.2.1.4

#### 6.2.1.4 Treatment discontinuation criteria

1. Any Grade 2 drug-related uveitis or eye pain or blurred vision that does not respond to topical therapy and does not improve to Grade 1 severity within the re-treatment period OR requires systemic treatment.
2. Any Grade 3 non-skin, drug-related adverse event lasting > 7 days, with the following exceptions:
   - Grade 3 drug-related uveitis, pneumonitis (including interstitial lung disease), bronchospasm, hypersensitivity reaction, or infusion reaction of any duration requires discontinuation.
   - Grade 3 drug-related endocrinopathies adequately controlled with only physiologic hormone replacement do not require discontinuation.
   - Grade 3 drug-related laboratory abnormalities do not require treatment discontinuation except Grade 3 drug-related thrombocytopenia > 7 days or associated with bleeding
3. Hepatotoxicity as evidenced by the following:
   - AST or ALT > 10 x ULN for > 2 weeks,
   - AST or ALT > 15 x ULN irrespective of duration,
   - Total bilirubin > 8 x ULN irrespective of duration for subjects with elevated bilirubin at

study entry or > 5 x ULN for those with normal Total bilirubin at entry,

- Concurrent AST or ALT > 3 x ULN and Total bilirubin > 5 x ULN for subjects entering treatment with a normal bilirubin and up to 8 x ULN for subjects with elevated bilirubin

4. Any Grade 4 drug-related adverse event or laboratory abnormality, except for the following events which do not require discontinuation:
   - Grade 4 neutropenia < 7 days
   - Grade 4 lymphopenia or leukopenia
   - Isolated Grade 4 amylase or lipase abnormalities that are not associated with symptoms or clinical manifestations of pancreatitis.
   - Isolated Grade 4 electrolyte imbalances or abnormalities that are not associated with clinical sequelae and are corrected with supplementation and appropriate management within 72 hours of their onset.
5. Any dosing delay lasting > 6 weeks with the following exceptions:
   - Dosing delays to allow for prolonged steroid tapers to manage drug related adverse events are allowed.
6. Any adverse event, laboratory abnormality, or intercurrent illness which, in the judgment of the Investigator, presents a substantial clinical risk to the subject with continued nivolumab dosing.
7. Investigator assessed disease progression according to RECIST 1.1
8. Immune mediated encephalitis
9. Recurrence of the same Grade 3 adverse event
10. Grade 2 or Grade 3 adverse event for more than 12 weeks
11. Grade 3 Myocarditis: Permanently discontinue

Tumor assessments for all subjects should continue as per protocol even if study drug dosing is discontinued

### 6.2.2 Regorafenib

#### 6.2.2.1. Dose reduction

Reduce the dose of regorafenib to 40 mg for followings

- For the first occurrence of Grade 2 HFSR of any duration
- After recovery of any Grade 3 or 4 adverse reaction to Grade 2
- For Grade 3 aspartate AST or ALT elevation; only resume if the potential benefit outweighs the risk of hepatotoxicity

#### 6.2.2.2 Dose delay criteria

Interrupt regorafenib for the following:

- Grade 2 hand-foot skin reaction (HFSR) [palmar-plantar erythrodysesthesia (PPE)] that is recurrent or does not improve within 7 days despite dose reduction; interrupt therapy for a minimum of 7 days for Grade 3 HFSR
- Symptomatic Grade 2 hypertension

- Any Grade 3 or 4 adverse reaction

#### 6.2.2.3 Treatment discontinuation criteria

Discontinue regorafenib permanently for the following:

- Failure to tolerate 40 mg dose
  Any occurrence of AST or ALT more than 20 times the ULN
- Any occurrence of AST or ALT more than 3 times ULN with concurrent bilirubin more than 2 times ULN
- Re-occurrence of AST or ALT more than 5 times ULN despite dose reduction to 40 mg
- For any Grade 4 adverse reaction; only resume if the potential benefit outweighs the risks

## 6.3 Toxicity Management

### 6.3.1 Nivolumab

*Immune-Mediated Pneumonitis*

Nivolumab can cause immune-mediated pneumonitis, defined as requiring use of corticosteroids and no clear alternate etiology. Fatal cases have been reported.

- Monitor patients for signs with radiographic imaging and for symptoms of pneumonitis.
- Administer corticosteroids at a dose of 1 to 2 mg/kg/day prednisone equivalents for moderate (Grade 2) or more severe (Grade 3-4) pneumonitis. Add prophylactic antibiotics for opportunistic infections in the case of prolonged steroid administration.
- When symptoms improve to Grade 1 or less, steroid taper should be started and continued over no less than 4 weeks.
- Permanently discontinue nivolumab for severe (Grade 3) or life-threatening (Grade 4) pneumonitis and withhold nivolumab until resolution for moderate (Grade 2) pneumonitis (see Section 6.2.1).

*Immune-Mediated Colitis*

Nivolumab can cause immune-mediated colitis, defined as requiring use of corticosteroids with no clear alternate etiology.

- Monitor patients for signs and symptoms of colitis (such as diarrhea, abdominal pain, blood or mucus in stool, with or without fever) and of bowel perforation (such as peritoneal signs and ileus).
- All patients who experience diarrhea/colitis should be advised to drink liberal quantities of clear fluids. If sufficient PO fluid intake is not feasible, fluid and electrolytes should be substituted via IV infusion.
- For Grade 2 or higher diarrhea, consider GI consultation and endoscopy to confirm or rule out colitis.
- For Grade 2 diarrhea/colitis that persists greater than 3 days, administer PO corticosteroids at a dose of 0.5 to 1 mg/kg/day prednisone equivalents; if worsening or no improvement occurs despite initiation of corticosteroids, increase dose to 1 to 2 mg/kg/day prednisone equivalents.

- For Grade 3 or 4 diarrhea/colitis that persists >1 week, treat with IV steroids at a dose of 1 to 2 mg/kg/day prednisone equivalents followed by high dose PO steroids.
- When symptoms improve to <Grade 1, steroid taper should be started and continued over no less than 4 weeks.
- Withhold nivolumab for moderate or severe (Grade 2 or 3) colitis. Permanently discontinue nivolumab for life-threatening (Grade 4) or for recurrent colitis upon re-initiation of nivolumab (see Section 6.2.1).

*Immune-Mediated Hepatitis*

Nivolumab can cause immune-mediated hepatitis, defined as requiring use of corticosteroids and no clear alternate etiology.

- Monitor patients for abnormal liver tests prior to and periodically during treatment.
- Administer corticosteroids at a dose of 0.5 to 1 mg/kg/day prednisone equivalents for moderate (Grade 2) transaminase elevations.
- Administer corticosteroids at a dose of 1 to 2 mg/kg/day prednisone equivalents followed by corticosteroid taper for severe (Grade 3) or life-threatening (Grade 4) transaminase elevations, with or without concomitant elevation in total bilirubin.
- When symptoms improve to Grade 1 or less, a steroid taper should be started and continued over no less than 4 weeks.
- Permanently discontinue, withhold, or continue nivolumab based on severity of immune-mediated hepatitis and baseline AST and ALT levels as described in Section 6.2.1.

*Immune-Mediated Endocrinopathies*

*Hypophysitis*

Nivolumab can cause immune-mediated hypophysitis.

- Monitor patients for signs and symptoms of hypophysitis.
- Administer hormone replacement as clinically indicated
- Corticosteroids at a dose of 1 mg/kg/day prednisone equivalents followed by corticosteroid taper for moderate (Grade 2) or greater hypophysitis.
- Withhold nivolumab for moderate (Grade 2) or severe (Grade 3). Permanently discontinue nivolumab for life-threatening (Grade 4) hypophysitis (see Section 6.2.1).

*Adrenal Insufficiency*

Nivolumab can cause immune-mediated adrenal insufficiency.

- Monitor patients for signs and symptoms of adrenal insufficiency.
- Administer corticosteroids at a dose of 1 to 2 mg/kg/day prednisone equivalents followed by a corticosteroid taper for severe (Grade 3) or life-threatening (Grade 4) adrenal insufficiency.
- Withhold nivolumab for moderate (Grade 2) and permanently discontinue nivolumab for severe (Grade 3) or life-threatening (Grade 4) adrenal insufficiency (see Section 6.2.1).

*Hypothyroidism and Hyperthyroidism*

Nivolumab can cause autoimmune thyroid disorders.

- Monitor thyroid function prior to and periodically during nivolumab treatment.
- Administer hormone-replacement therapy for hypothyroidism.
- In Grade 2 hyperthyroidism, non-selective beta-blockers (e.g., propranolol) are suggested as initial therapy.
- For Grade 3 hyperthyroidism, treat with an initial dose of IV corticosteroid followed by PO corticosteroids.
- Permanently discontinue nivolumab for Grade 4 hypo/hyperthyroidism.
- When symptoms improve to Grade 1, steroid taper should be started and continued over no less than 4 weeks.
- There are no recommended dose adjustments of nivolumab for hypothyroidism or hyperthyroidism.

*Type 1 Diabetes Mellitus*

Nivolumab can cause Type 1 diabetes mellitus.

- Monitor for hyperglycemia.
- Insulin replacement therapy is recommended for T1 DM and for Grade 3-4 hyperglycemia associated with metabolic acidosis or ketonuria.
- Evaluate patients with serum glucose and a metabolic panel, urine ketones glycosylated hemoglobin, and C-peptide.
- Withhold nivolumab in cases of severe (Grade 3) hyperglycemia until metabolic control is achieved.
- Permanently discontinue Nivolumab for life-threatening (Grade 4) hyperglycemia (see Section 6.2.1).

*Immune-Mediated Nephritis and Renal Dysfunction*

Nivolumab can cause immune-mediated nephritis, defined as renal dysfunction or ≥ Grade 2 increased creatinine, requirement for corticosteroids, and no clear alternate etiology.

- Monitor patients for elevated serum creatinine prior to and periodically during treatment.
- Administer corticosteroids at a dose of 0.5 to 1 mg/kg/day prednisone equivalents for moderate (Grade 2) or severe (Grade 3) increased serum creatinine, if worsening or no improvement occurs, increase dose of corticosteroids to 1 to 2 mg/kg/day prednisone equivalents.
- Administer corticosteroids at a dose of 1 to 2 mg/kg/day prednisone equivalents followed by corticosteroid taper for life-threatening (Grade 4) increased serum creatinine.
- When symptoms improve to Grade 1 or less, steroid taper should be started and continued over no less than 4 weeks.
- Withhold nivolumab for moderate (Grade 2).

- Permanently discontinue nivolumab for life-threatening (Grade 3-4) increased serum creatinine (see Section 6.2.1).

*Immune-Mediated Skin Adverse Reactions*

Nivolumab can cause immune-mediated rash, including Stevens-Johnson syndrome (SJS) and toxic epidermal necrolysis (TEN), some cases with fatal outcome.

- For symptoms or signs of SJS or TEN, withhold nivolumab and refer the patient for specialized care for assessment and treatment.
- If SJS or TEN is confirmed, permanently discontinue nivolumab (see Section 6.2.1).
- For immune-mediated rash, administer corticosteroids at a dose of 1 to 2 mg/kg/day prednisone equivalents followed by a corticosteroid taper for severe (Grade 3) or life-threatening (Grade 4) rash.
- Withhold nivolumab for severe (Grade 3) rash and permanently discontinue nivolumab for life- threatening (Grade 4) rash.

*Immune-Mediated Encephalitis*

Nivolumab can cause immune-mediated encephalitis with no clear alternate etiology.

- Evaluation of patients with neurologic symptoms may include, but not be limited to, consultation with a neurologist, brain MRI, and lumbar puncture.
- Withhold nivolumab in patients with new-onset moderate to severe neurologic signs or symptoms and evaluate to rule out infectious or other causes of moderate to severe neurologic deterioration.
- If other etiologies are ruled out, administer corticosteroids at a dose of 1 to 2 mg/kg/day prednisone equivalents for patients with immune-mediated encephalitis, followed by corticosteroid taper.
- Permanently discontinue nivolumab for immune-mediated encephalitis (see Section 6.2.1).

*Other Immune-Mediated Adverse Reactions*

Nivolumab can cause other clinically significant and potentially fatal immune-mediated adverse reactions. Immune-mediated adverse reactions may occur after discontinuation of nivolumab therapy. For any suspected immune-mediated adverse reactions, exclude other causes.

Based on the severity of the adverse reaction, permanently discontinue or withhold nivolumab, administer high-dose corticosteroids, and if appropriate, initiate hormone-replacement therapy. Upon improvement to Grade 1 or less, initiate corticosteroid taper and continue to taper over at least 1 month. Consider restarting nivolumab after completion of corticosteroid taper based on the severity of the event (see Section 6.2.1).

Across clinical trials of nivolumab administered as a single agent or in combination with ipilimumab, the following clinically significant immune-mediated adverse reactions, some with fatal outcome, occurred in less than 1.0% of patients receiving nivolumab: myocarditis, rhabdomyolysis, myositis, uveitis, iritis, pancreatitis, facial and abducens nerve paresis, demyelination, polymyalgia rheumatica, autoimmune neuropathy, Guillain-Barré syndrome, hypopituitarism, systemic inflammatory response syndrome, gastritis, duodenitis, sarcoidosis, histiocytic necrotizing lymphadenitis (Kikuchi

lymphadenitis), motor dysfunction, vasculitis, aplastic anemia, pericarditis, and myasthenic syndrome. If uveitis occurs in combination with other immune-mediated adverse reactions, consider a Vogt-Koyanagi-Harada-like syndrome, which has been observed in patients receiving nivolumab or nivolumab in combination with ipilimumab and may require treatment with systemic steroids to reduce the risk of permanent vision loss.

*Infusion Reactions*

Since nivolumab contains only human immunoglobulin protein sequences, it is unlikely to be immunogenic and induce infusion or hypersensitivity reactions. However, if such a reaction were to occur, it might manifest with fever, chills, rigors, headache, rash, pruritus, arthalgias, hypotension, hypertension, bronchospasm, or other allergic-like reactions. All Grade 3 or 4 infusion reactions should be reported within 24 hours to the study medical monitor and reported as an SAE if it meets the criteria. Infusion reactions should be graded according to NCI-CTCAE v5.0 guidelines.

Treatment recommendations are provided below and may be modified based on local treatment standards and guidelines, as appropriate:

1. For Grade 1 symptoms: (Mild reaction; infusion interruption not indicated; intervention not indicated).
   - Remain at bedside and monitor subject until recovery from symptoms. The following prophylactic premedication is recommended for future infusions: diphenhydramine 50 mg . (or equivalent) and/or acetaminophen/paracetamol 325. to 1000 mg at least 30 minutes before additional nivolumab administrations.

2. For Grade 2 symptoms: (Moderate reaction requires therapy or infusion interruption but responds promptly to symptomatic treatment [eg, antihistamines, non-steroidal anti-inflammatory drugs, narcotics, corticosteroids, bronchodilators, IV fluids]; prophylactic medications indicated for ≤24 hours).
   - Stop the nivolumab infusion, begin an IV infusion of normal saline, and treat the subject with diphenhydramine 50 mg IV (or equivalent) and/or acetaminophen/paracetamol 325 to 1000 mg; remain at bedside and monitor subject until resolution of symptoms. Corticosteroid and/or bronchodilator therapy may also be administered as appropriate.
   - If the infusion is interrupted, then restart the infusion at 50% of the original infusion rate when symptoms resolve; if no further complications ensue after 30 minutes, the rate may be increased to 100% of the original infusion rate. Monitor subject closely. If symptoms recur, then no further nivolumab will be administered at that visit.
   - For future infusions, the following prophylactic premedication is recommended: diphenhydramine 50 mg (or equivalent) and/or acetaminophen/paracetamol 325 to 1000 mg should be administered at least 30 minutes before nivolumab infusions. If necessary, corticosteroids (up to 25 mg of IV hydrocorticone or equivalent) may be used.

3. For Grade 3 or 4 symptoms: (Severe reaction, Grade 3: prolonged [ie, not rapidly responsive to symptomatic medication and/or brief interruption of infusion]; recurrence of symptoms following initial improvement; hospitalization indicated for other clinical sequelae [eg, renal impairment, pulmonary infiltrates]. Grade 4: Life-threatening; pressor or ventilatory support

indicated).

- Immediately discontinue infusion of nivolumab. Begin an IV infusion of normal saline and treat the subject as follows: Recommend bronchodilators, epinephrine 0.2 to 1 mg of a 1:1000 solution for subcutaneous administration or 0.1 to 0.25 mg of a 1:10,000 solution injected slowly for IV administration, and/or diphenhydramine 50 mg IV with methylpredni solone 100 mg IV (or equivalent), as needed. Subject should be monitored until the Investigator is comfortable that the symptoms will not recur.
- Nivolumab will be permanently discontinued. Investigators should follow their institutional guidelines for the treatment of anaphylaxis. Remain at bedside and monitor subject until recovery of the symptoms.

4. In case of late-occurring hypersensitivity symptoms (eg, appearance of a localized or generalized pruritus within 1 week after treatment), symptomatic treatment may be given (eg, oral antihistamine or corticosteroids).

### 6.3.2 Regorafenib

*Hepatotoxicity*

Severe drug induced liver injury with fatal outcome occurred in 0.3% of 1100 Stivarga-treated patients across all clinical trials.

Obtain liver function tests (ALT, AST and bilirubin) before initiation of regorafenib and monitor at least every two weeks during the first 2 months of treatment. Thereafter, monitor monthly or more frequently as clinically indicated. Monitor liver function tests weekly in patients experiencing elevated liver function tests until improvement to less than 3 times the ULN or baseline.

Temporarily hold and then reduce or permanently discontinue regorafenib depending on the severity and persistence of hepatotoxicity as manifested by elevated liver function tests or hepatocellular necrosis (see Section 6.2.2.)

*Hemorrhage*

Regorafenib caused an increased incidence of hemorrhage. The overall incidence (Grades 1-5) was 21% in regorafenib-treated patients compared to 8% in placebo-treated patients. Fatal hemorrhage occurred in 4 of 500 (0.8%) of regorafenib- treated patients and involved the respiratory, gastrointestinal, or genitourinary tracts.

Permanently discontinue regorafenib in patients with severe or life-threatening hemorrhage.

*Dermatological Toxicity*

Regorafenib caused an increased incidence of hand-foot skin reaction (HFSR) also known as palmar-plantar erythrodysesthesia (PPE) and rash frequently requiring dose modification. The overall incidence of HFSR (45% versus 7%) and the incidence of Grade 3 HFSR (17% versus 0) were increased in regorafenib-treated patients. The overall incidence of rash (26% versus 4%) and the incidence of Grade 3 rash (6% versus <1%) were higher in regorafenib- treated patients). The onset of dermatologic toxicity occurred in the first cycle of treatment in most patients.

Temporarily hold and then reduce or permanently discontinue regorafenib depending on the severity and persistence of dermatologic toxicity (see Section 6.2.2). Institute supportive measures for

symptomatic relief.

*Hypertension*

Regorafenib caused an increased incidence of hypertension (30% of regorafenib-treated patients vs. 8% of placebo-treated patients). Hypertensive crisis occurred in 0.18% of 1100 regorafenib-treated patients across all clinical trials. The onset of hypertension occurred during the first cycle of treatment in most patients.

Do not initiate regorafenib until blood pressure is adequately controlled.

Monitor blood pressure weekly for the first 6 weeks of treatment and then every cycle, or more frequently, as clinically indicated. Temporarily or permanently withhold regorafenib for severe or uncontrolled hypertension (see Section 6.2.2).

*Cardiac Ischemia and Infarction*

Regorafenib increased the incidence of myocardial ischemia and infarction (1.2% for regorafenib-treated patients vs. 0.4% of placebo-treated patients).

Withhold regorafenib in patients who develop new or acute onset cardiac ischemia or infarction.

*Reversible Posterior Leukoencephalopathy Syndrome (RPLS)*

RPLS (also known as posterior reversible encephalopathy syndrome) occurred in one of 1100 regorafenib-treated patients across all clinical trials.

Confirm the diagnosis of RPLS with MRI and discontinue regorafenib in patients who develop RPLS.

*Gastrointestinal Perforation or Fistula*

Gastrointestinal perforation or fistula occurred in 0.6% of 1100 patients treated with regorafenib across clinical trials.

Permanently discontinue Regorafenib in patients who develop gastrointestinal perforation or fistula.

*Wound Healing Complications*

No formal studies of the effect of regorafenib on wound healing have been conducted.

Since vascular endothelial growth factor receptor (VEGFR) inhibitors such as regorafenib can impair wound healing, treatment with regorafenib should be stopped at least 2 weeks prior to scheduled surgery. The decision to resume regorafenib after surgery should be based on clinical judgment of adequate wound healing. Regorafenib should be discontinued in patients with wound dehiscence.

## 6.4 Duration of Treatment

Study treatment will be administered until the tumor progression, or unacceptable toxicities. For patients who showed disease control > 2 years with regorafenib-nivolumab combination therapy, discontinuation of nivolumab may be considered at the discretion of investigators.

## 6.5 Discontinuation of Treatment

Upon treatment discontinuation, all end of treatment evaluations and tests will be conducted. All

participants who discontinue due to an AE must be followed until the event resolves or stabilizes. Appropriate medical care should be provided until signs and symptoms have abated, stabilized, or until abnormal laboratory findings have returned to acceptable or pre-study limits. The final status of the AE will be reported in the participant's medical records and the appropriate CRF.

Reasons for treatment discontinuation should be classified as follows:

- Death
- Progressive disease
- Toxicity; treatment related or unrelated
- Investigator judgment
  - The Investigator may discontinue a participant if, in his/her judgment, it is in the best interest of the participant to do so.
- Noncompliance
- Participant voluntary withdrawal
  - A participant may withdraw from the study at any time, for any reason. If a participant discontinues treatment, an attempt should be made to obtain information regarding the reason for withdrawal.

## 6.6 Concomitant Treatment

### 6.6.1 Prohibited treatment

| **Prohibited medication/class of drug:** | **Usage:** |
|---|---|
| Any anticancer therapy including chemotherapy, radiotherapy, immunotherapy, biologic therapy or hormone therapy other than those under investigation in this study | Should not be given concomitantly whilst the patient is on study treatment<br>- Concurrent use of hormones for non-cancer-related conditions [e.g., insulin for diabetes and hormone replacement therapy] is acceptable.<br>- Local treatment of isolated lesions, excluding target lesions, for palliative intent is acceptable (e.g., by local surgery or radiotherapy) |

| **Prohibited medication/class of drug:** | **Usage:** |
|---|---|
| Immunosuppressive medications including, but not limited to, systemic corticosteroids at immunosuppressive doses, methotrexate, azathioprine, and tumor necrosis factor-α blockers | Should not be given concomitantly or used for premedication prior to the nivolumab infusions. The following are allowed exceptions:<br>- Use of immunosuppressive medications for the management of IP-related AEs,<br>- Use in patients with contrast allergies.<br>- In addition, use of inhaled, topical, and intranasal corticosteroids is permitted.<br><br>A temporary period of steroids will be allowed if clinically indicated and considered to be essential for the management of non-immunotherapy related events experienced by the patient (e.g., chronic obstructive pulmonary disease, radiation, nausea, etc.). |
| Any botanical preparation | - Any botanical preparation (eg herbal supplements or traditional Chinese medicines) intended to treat the disease under study or provide supportive care. Use of marijuana and its derivatives for treatment of symptoms related to cancer or cancer treatment are permitted if obtained by medical prescription or if its use (even without a medical prescription) has been legalized locally. |
| Strong CYP3A4 inducers (e.g. rifampin, phenytoin, carbamazepine, phenobarbital, and St. John's Wort) and inhibitors (e.g. clarithromycin, grapefruit juice, itraconazole, ketoconazole, posaconazole, telithromycin, and voriconazole) | Should be avoided concomitantly whilst the patient is on study treatment unless deemed to be absolutely necessary |
| Live attenuated vaccines | Should not be given through 30 days prior to the first dose, during the study treatment and until 100 days after the last dose of IP. |

### 6.6.2 Permitted treatment

| **Supportive medication/class of drug:** | **Usage:** |
|---|---|
| Concomitant medications or treatments (e.g., acetaminophen or diphenhydramine) deemed necessary to provide adequate prophylactic or supportive care, except for those medications identified as "prohibited," as listed above | To be administered as prescribed by the Investigator |

| | |
|---|---|
| Best supportive care (including antibiotics, nutritional support, correction of metabolic disorders, optimal symptom control, and pain management) | Should be used, when necessary, for all patients |
| Palliative radiotherapy to non-target lesions | Permitted for symptomatic control, provided that the radiotherapy does not affect target lesion, and the reason for the radiotherapy does not reflect progressive disease |
| Inactivated viruses, such as those in the influenza vaccine | Permitted |

## Monitoring compliance

Compliance with regorafenib will be monitored by tablet counts after every 4-week cycles. Patients will be instructed to bring their medication bottles to each visit, and a new bottle filled with regorafenib for next cycle will be provided. Remaining regorafenib tablets will be counted and recorded by investigators.

# 7.0 STUDY PROCEDURE

## 7.1 Schedule of study procedures

Tumor efficacy (RECIST) assessment dates are not affected by dose delays and remain as originally scheduled, as they are based on the start date (i.e., C1D1) of therapy.

All other scheduled assessments must be performed relative to the start of the dosing cycle such that all laboratory procedures, etc. required for dosing should be performed within 3 days prior to dosing.

Dosing may be delayed per Toxicity Management Guidelines, due to either an immune or a non-immune-related AE. If dosing must be delayed for reasons other than treatment-related toxicity, dosing will occur as soon as feasible.

### 7.1.1 Screening phase

Screening procedures will be performed up to 28 days before Day 1, unless otherwise specified. All patients must first read, understand, and sign the IRB-approved ICF before any study-specific screening procedures are performed. After signing the ICF, completing all screening procedures, and being deemed eligible for entry, patients will be enrolled in the study. Procedures that are performed prior to the signing of the ICF and are considered standard of care may be used as screening assessments if they fall within the 28-day screening window.

The following procedures will be performed during the Screening Visit:

- Informed Consent
- Review of eligibility criteria
- Medical history and demographics
- Complete physical exam
- ECOG Performance Status
- Vital signs, weight and height
- 12-lead ECG
- Review of prior/concomitant medications
- Imaging including, but not limited to abdomen-pelvis CT, chest CT, brain MRI, bone scan
- Clinical laboratory tests for:
    - Hematology
    - Clinical chemistry
    - TSH, free T4
    - Coagulation
    - Serum pregnancy test (for women of childbearing potential only)
    - Hepatitis and HIV serologies
    - Urinalysis
    - AFP, PIVKA-II

### 7.1.2 Treatment phase

Procedures to be conducted during the treatment phase of the study are presented in the Schedule of

Assessments. Screening procedures performed within 7 days of Cycle 1 Day 1 (C1D1) do not need to be repeated on C1D1.

### 7.1.3 End of treatment

End of treatment is defined as the last visit where the decision is made to discontinue treatment. All required procedures may be completed within ±7 days of the end of treatment visit. Repeat disease assessment is not required if performed within 28 days prior to the end of treatment visit.

Assessments for patients who have discontinued treatment due to PD or unacceptable toxicities are presented in Section 9. In combination therapy, treatment of the other drug will continue if unacceptable toxicity caused by one drug.

All patients will be followed for survival until the end of the study regardless of further treatments, or until the sponsor ends the study.

## 7.2 Description of study procedures

### 7.2.1 Medical history and physical examination, electrocardiogram, weight, and vital signs

Findings from medical history (obtained at screening) and physical examination shall be given a baseline grade according to the procedure for AEs. Increases in severity of pre-existing conditions during the study will be considered AEs, with resolution occurring when the grade returns to the pre-study grade or below.

Physical examinations will be performed on study days noted in the Schedule of Assessments. A complete physical examination will be performed and will include an assessment of the following (as clinically indicated): general appearance, respiratory, cardiovascular, abdomen, skin, head and neck (including ears, eyes, nose and throat), lymph nodes, thyroid, musculoskeletal (including spine and extremities), genital/rectal, and neurological systems and at screening only, height.

Resting 12-lead ECGs will be recorded at screening and as clinically indicated throughout the study. At Screening, a single ECG will be obtained on which QTcF must be < 500 ms. In case of clinically significant ECG abnormalities, including a QTcF value >500 ms, 2 additional 12-lead ECGs should be obtained over a brief period (eg, 30 minutes) to confirm the finding. Situations in which ECG results should be reported as AEs are described in Section 9.

Vital signs (blood pressure [BP], pulse, temperature, and respiration rate) will be evaluated according to the assessment schedules. Body weight is also recorded at each visit along with vital signs.

<u>First infusion</u>

On the first infusion day, patients will be monitored, and vital signs collected/recorded in eCRF prior to, during and after infusion of nivolumab as below at the following times (based on a 60-minute infusion):

- Prior to the beginning of the infusion (measured once from approximately 30 minutes before up to 0 minutes [i.e., the beginning of the infusion])

- Approximately 30 minutes during the infusion (halfway through infusion)
- At the end of the infusion (approximately 60 minutes ±5 minutes)

If the infusion takes longer than 60 minutes, then BP and pulse measurements should follow the principles as described above or be taken more frequently if clinically indicated. A 1-hour observation period is recommended after the first infusion of nivolumab.

Subsequent infusions

BP, pulse and other vital signs should be measured, collected/recorded in eCRF prior to the start of the infusion. Patients should be carefully monitored, and BP and other vital signs should be measured during and post infusion as per institution standard and as clinically indicated.

### 7.2.2 Clinical laboratory tests

The following clinical laboratory tests will be performed (see the Schedule of Assessments for the timepoints of each test):

| Hematology Laboratory Tests | |
|---|---|
| Basophils [a] | Mean corpuscular volume |
| Eosinophils [a] | Monocytes [a] |
| Hematocrit | Neutrophils [a] |
| Hemoglobin | Platelet count |
| Lymphocytes [a] | Red blood cell count |
| Mean corpuscular hemoglobin | Total white cell count |
| Mean corpuscular hemoglobin concentration | |

[a] Can be recorded as absolute counts or as percentages.

| Coagulation Parameters |
|---|
| Activate partial thromboplastin |
| Prothrombin time (international normalized ratio) |

Coagulation parameters are to be assessed at baseline, and as clinically indicated.

| Clinical Chemistry (Serum or Plasma) Laboratory Tests | |
|---|---|
| Albumin | Glucose |
| Alkaline phosphatase | Lactate dehydrogenase |
| Alanine aminotransferase | Lipase [b] |
| Amylase [b] | Magnesium [c] |
| Aspartate aminotransferase | Potassium |

| Clinical Chemistry (Serum or Plasma) Laboratory Tests | |
|---|---|
| Bicarbonate [c] | Sodium |
| Calcium | Total bilirubin [a] |
| Chloride [c] | Total protein |
| Creatinine | TSH |
| Free T4 | Urea or blood urea nitrogen, depending on local practice |
| Gamma glutamyltransferase [c] | Uric acid |

[a] Tests for ALT, AST, alkaline phosphatase, and total bilirubin must be conducted and assessed concurrently. If total bilirubin is ≥2 × upper limit of normal then fractionate into direct and indirect bilirubin.

[b] It is preferable that both amylase and lipase parameters are assessed. For sites where only 1 of these parameters is routinely measured then either lipase or amylase is acceptable.

[c] Bicarbonate (where available), chloride, gamma glutamyltransferase, and magnesium testing are to be performed at baseline, on Day 0 (unless all screening laboratory clinical chemistry assessments are performed within 3 days prior to Day 0), and if clinically indicated.

[d] If TSH and free T4 is measured within 14 days prior to Day 1 (first infusion day), it does not need to be repeated.

| Urinalysis Tests [a] | |
|---|---|
| Bilirubin | pH |
| Blood | Protein |
| Glucose | Specific gravity |
| Ketones | Colour and appearance |

[a] Microscopy should be used as appropriate to investigate white blood cells and use the high-power field for red blood cells

| Tumor Marker |
|---|
| Alpha-fetoprotein (AFP) |
| Protein induced by vitamin K absence-II (PIVKA-II) |

All patients should have further chemistry profiles performed at 30 days (±3 days), 2 months (±1 week) and 3 months (±1 week) after permanent discontinuation of IP.

Any clinically significant abnormal laboratory values should be repeated as clinically indicated and recorded on the eCRF. Situations in which laboratory safety results should be reported as AEs are described in Section 9.

All patients with Grade 3 or 4 laboratory values at the time of completion or discontinuation from IP must have further tests performed until the laboratory values have returned to Grade 1 or 2, unless these values are not likely to improve because of the underlying disease.

## 7.3 Biological Sampling Procedures

### 7.3.1 Sample collection

Up to 30 tissue slides from archival tissues from previous surgery or biopsy will be collected.

20 ml of blood will be collected until the 3rd cycle (1 cycle = 4 weeks) as blow:

- Baseline (within - 7 days from the first dose)
- Cycle 1 Day 15 (2 weeks from the first dose)
- Cycle 2 Day 1 (4 weeks from the first dose)
- Cycle 3 Day 1 (8 weeks from the first dose)

### 7.3.2 Exploratory analysis

1. Biomarker analysis using PBMC
   - 20 ml of blood will be collected until 3 cycles (1 cycle=4 weeks): baseline, C1D15 (2 weeks), C2D1 (4 weeks), C3D1 (8 weeks)
   - FACS and RNA sequencing analysis will reveal the change of immune cell subpopulation or gene signatures before and after the study treatment
   - Circulating tumor DNA analysis using baseline sample

2. Biomarker analysis using tissue
   - Using archival tumor tissues, multiplexed immunohistochemistry will be performed for immune cell subset analysis.
   - If patient number with archival tumor tissues are not sufficient for analysis, this will not be performed.

### 7.3.3 Withdrawal of informed consent for donated biological samples

If a patient withdraws consent to the use of donated samples, the samples will be disposed of, and the action documented. As collection of the biological samples is an integral part of the study, then the patient is withdrawn from further study participation.
The Principal Investigator:

- Ensures that biological samples from that patient, if stored at the study site, are immediately identified, disposed of /destroyed, and the action documented
- Ensures the laboratory(ies) holding the samples is/are informed about the withdrawn consent immediately and that samples are disposed/destroyed, the action documented, and the signed document returned to the study site
- Ensures that the patient is informed about the sample disposal.

## 8.0 ASSESSMENT OF EFFICACY

Tumor evaluation using CT and/or MRI will be performed every 8 weeks. Imaging studies will be done whenever there is a sign or symptom suggesting tumor progression. The response to study treatment will be assessed as complete response (CR), partial response (PR), stable disease (SD), or progressive disease (PD) based on RECIST 1.1 at every time points.

Best responses defined as the best response across all time points. Confirmation of complete or partial response is not required. When SD is believed to be best response, it must meet the minimum time of 4 weeks from baseline.

After discontinuation/study completion, survival status of patients will be updated every 3 months.

It is allowed to continue regorafenib-nivolumab combination treatment beyond 1$^{st}$ PD when investigators assess that there is a potential clinical benefit. In that case, if the next scan shows further progression, then the date of the prior scan with PD should be declared as the date of progression.

# 9.0 ASSESSMENT OF SAFETY

Safety evaluation will be performed at every visit and graded by NCI-CTCAE v5.0.

## 9.1 Definition of safety parameters.

### 9.1.1 Adverse Event

Any untoward medical occurrence in a patient or clinical investigation subject administered a pharmaceutical product and which does not necessarily have to have a causal relationship with this treatment. An adverse event (AE) can therefore be any unfavorable and unintended sign (including an abnormal laboratory finding, for example), symptom, or disease temporally associated with the use of a medicinal product, whether or not considered related to the medicinal product.

### 9.1.2 Serious Adverse Event

A serious adverse event is an AE occurring during any study phase (i.e., screening, run-in, treatment, wash-out, follow-up), at any dose of the study drugs that fulfils one or more of the following criteria:

1. results in death
2. is immediately life-threatening
3. requires in-patient hospitalization or prolongation of existing hospitalization
4. results in persistent or significant disability or incapacity
5. is a congenital abnormality or birth defect in offspring of the patient
6. is an important medical event that may jeopardize the patient or may require medical intervention to prevent one of the outcomes listed above.
   - Medical or scientific judgment should be exercised in deciding whether expedited reporting is appropriate in this situation. Examples of medically important events are intensive treatment in an emergency room or at home for allergic bronchospasm, blood dyscrasias, or convulsions that do not result in hospitalizations; or development of drug dependency or drug abuse.

## 9.2 Grading

The descriptions and grading scales found in the NCI-CTCAE v5.0 will be utilized for AE reporting. AEs not covered by specific terminology listed should be reported with common medical terminology and documented according to the grading scales provided in the NCI-CTCAE v5.0 as below:

| | |
|---|---|
| Grade 1 (mild) | An event that is usually transient and may require only minimal t reatment or therapeutic intervention. The event does not generally interfere with usual activities of daily living. |
| Grade 2 (moderate) | An event that is usually alleviated with additional specific therape utic intervention. The event interferes with usual activities of dail y living, causing discomfort but poses no significant or permanent risk of harm to the patient. |

Grade 3 (severe) — An event that requires intensive therapeutic intervention. The even t interrupts usual activities of daily living, or significantly affects the clinical status of the patient.

Grade 4 (life-threatening) — An event, and/or its immediate sequelae, that is associated with a n imminent risk of death or with physical or mental disabilities th at affect or limit the ability of the patient to perform activities of daily living (eating, ambulation, toileting, etc).

Grade 5 (fatal) — Death (loss of life) as a result of an event.

It is important to distinguish between serious criteria and severity of an AE. Severity is a measure of intensity whereas seriousness is defined by the criteria in Section 10.1.2. A Grade 3 AE need not necessarily be considered an SAE. For example, a Grade 3 headache that persists for several hours may not meet the regulatory definition of an SAE and would be considered a nonserious event, whereas a Grade 2 seizure resulting in a hospital admission would be considered an SAE.

## 9.3 Relationship to Drug

The relationship of event to study drug will be documented by the Investigator as follows:

1. Related: there is a relationship between suspect drug and adverse event
2. Not related: there is no relationship between suspect drug and adverse event

## 9.4 Recording Safety Parameters

### 9.4.1 Recording period and follow-up for safety parameters

Serious adverse events (SAEs) will be recorded from the time of the signature of informed consent, throughout the treatment period and including the follow-up period (30 days after the last dose of investigational product). If an event that occurs after the safety follow-up period, then it should be reported as an SAE as applicable.

During the course of the study all SAEs should be proactively followed up for each patient. SAEs should be reported to the Ethics Committee(s) and/or competent authorities and ONO pharma Korea/Bayer Korea within 24 hours of investigator becoming aware of the SAE. Every effort should be made to obtain a resolution for all events, even if the events continue after discontinuation/study completion.

For reporting SAEs to ONO pharma Korea/Bayer Korea, fulfil following description;

All SAEs occurring from the date the participant received study drug and until 30 days after the last intervention or a new treatment is started, whichever comes first, will be reported to ONO pharma Korea. The report is to be completed with all available information, including a brief narrative describing the SAE and any other relevant information by using attachment 1.

Reporting timeline and contact point is like below.

- Reporting timeline: Within 24 hours of becoming aware of SAE
- Reporting contact point: pv-opkr@ono.co.jp

The investigator is responsible for following up all SAEs until resolution, or until the event is assessed as stable or irreversible. There is no obligation for the investigator to actively report information on new SAEs occurring in former study patients after the 30-day safety follow-up period. However, if an investigator learns of any SAEs, including death, at any time after the patient has been permanently withdrawn from study, and he/she considers there is a reasonable possibility that the event is relat ed to study treatment, the investigator should notify the study sponsor.

For reporting SUSARs to MFDS, Sponsor (investigator) should bear the responsibility to report Suspected Unexpected Serious Adverse Reaction (SUSAR) to MFDS as per Korea Good Clinical Practice. Sponsor should report all SUSAR to MFDS and if needed, also to IRB.

### 9.4.2 Adverse events based on signs and symptoms

All AEs spontaneously reported by the patient or reported in response to the open question from the study personnel: "Have you had any health problems since the previous visit/you were last asked?" or revealed by observation will be collected and recorded in the eCRF.

If known, a diagnosis should be recorded on the CRF rather than individual signs and symptoms (e.g., record only liver failure or hepatitis rather than jaundice, asterixis, and elevated transaminases). However, if a constellation of signs and/or symptoms cannot be clinically characterized as a single diagnosis or syndrome at the time of reporting, each individual event should be recorded as an AE or SAE on the CRF. If a diagnosis is subsequently established, it should be reported as follow-up information.

In general, AEs occurring secondary to other events (e.g., cascade events or clinical sequelae) should be identified by their primary cause. For example, if severe diarrhea is known to have resulted in dehydration, it is sufficient to record only diarrhea as an AE or SAE on the CRF.

However, clinically significant AEs occurring secondary to an initiating event that are separated in time should be recorded as independent events on the CRF. (e.g., if a severe gastrointestinal hemorrhage leads to renal failure, both events should be recorded separately on the CRF.)

### 9.4.3 Adverse events based on examinations and tests

Any new or aggravated clinically relevant abnormal medical finding at a physical examination as compared with the baseline assessment will be reported as an AE.

The results from protocol-mandated laboratory tests and vital signs measurements will be summarized in the CSR. Therefore, only clinically significant laboratory abnormalities that require active management will be recorded as SAEs on the CRF (e.g., abnormalities that require study drug dose modification, discontinuation of study treatment, more frequent follow-up assessments, further diagnostic investigation, etc.).

- If the clinically significant laboratory abnormality is a sign of a disease or syndrome (e.g., alkaline phosphatase and bilirubin 5 x the upper limit of normal associated with cholecystitis), only the diagnosis (e.g., cholecystitis) needs to be recorded on the Adverse

Event CRF.

- If the clinically significant laboratory abnormality is not a sign of a disease or syndrome, the abnormality itself should be recorded as an SAE on the CRF. If the laboratory abnormality can be characterized by a precise clinical term, the clinical term should be recorded as the AE or SAE. For example, an elevated serum potassium level of 7 mEq/L should be recorded as “hyperkalemia.” Observations of the same clinically significant laboratory abnormality from visit to visit should not be repeatedly recorded as AEs or SAEs on the CRF, unless their severity, seriousness, or etiology changes.

Deterioration of a laboratory value that is unequivocally due to disease progression should not be reported as an AE/SAE.

### 9.4.4 Preexisting Medical Conditions (Baseline Conditions)

A preexisting medical condition should be recorded as an SAE only if the frequency, severity, or character of the condition worsens during the study. When recording such events on an Serious Adverse Event CRF, it is important to convey the concept that the preexisting condition has changed by including applicable descriptors (e.g., “more frequent headaches”).

### 9.4.5 New cancers

The development of a new cancer should be regarded as an SAE. New primary cancers are those that are not the primary reason for the administration of the IP and have been identified after the patient’s inclusion in this study.

### 9.4.6 Death

All deaths that occur during the study treatment period, or within the protocol-defined follow-up period after the administration of the last dose of study drug, must be reported as follows:

- Death clearly resulting from disease progression should be reported to the Study Monitor/Physician at the next monitoring visit and should be documented in the eCRF in the Statement of Death page. It should not be reported as an SAE.
- Where death is not due (or not clearly due) to progression of the disease under study, the AE causing the death must be reported to the Study Monitor/Physician as an SAE within 24 hours. It should also be documented in the Statement of Death page in the eCRF. The report should contain a comment regarding the co involvement of PD, if appropriate, and should assign main and contributory causes of death.
- Deaths with an unknown cause should always be reported as an SAE. It should also be documented in the Statement of Death page in the eCRF. A post mortem may be helpful in the assessment of the cause of death, and if performed, a copy of the post-mortem results should be forwarded to ONO pharma Korea/Bayer Korea Patient Safety or its representative within the usual timeframes.

Deaths occurring after the protocol defined safety follow up period after the administration of the last dose of study drug should be documented in the Statement of Death page. If the death occurred as a result of an event that started after the defined safety follow up period and the event is considered to be due to a late onset toxicity to study drug, then it should also be reported as an SAE. For SAE reporting

timeline and reporting contact information, please refer to 9.4.1.

## 9.5 Other events requiring reporting

### 9.5.1 Pregnancy

If pregnancy occur, it must be reported to OPKR.

The Principal Investigator shall report pregnancy event within twenty-four (24) hours of becoming aware of the pregnancy by using the Pregnancy reporting form (Attachment 2).

As for the pregnancy, the Principal Investigator shall follow up the pregnancy until delivery or the end of pregnancy.

Protocol-required procedures for study discontinuation and follow-up must be performed on the subject unless contraindicated by pregnancy (e.g., x-ray studies).

Other appropriate pregnancy follow-up procedures should be considered if indicated.

Follow-up information regarding the course of the pregnancy, including perinatal and neonatal outcome and, where applicable, offspring information must be recorded as much as possible.

- Reporting timeline: Within 24 hours of becoming aware of pregnancy
- Reporting contact point: pv-opkr@ono.co.jp

# 10.0 STATISTICS

## 10.1 Description of analysis sets

The analysis populations are specified below. The final decision to exclude participants from any analysis population will be made during a blinded data review meeting prior to the database lock date.

### 10.1.1 Safety analysis set

All participants, who were administered any dose of any study intervention will be candidates for safety analysis. Analyses will consider these participants as treated.

### 10.1.2 Efficacy analysis set

Efficacy analysis will be performed on the intent-to-treat (ITT) population. All participants will be candidates for efficacy analysis.

## 10.2 Methods of statistical analyses

### 10.2.1 Safety analysis

| **Endpoint** | **Statistical Analysis Methods** |
|---|---|
| **Secondary** | |
| Toxicity profile by the | Toxicity profiles will be evaluated every visit with physical examination, |

| | |
|---|---|
| NCI-CTCAE v5.0 | vital signs, ECOG performance status, complete blood count, serum electrolyte, serum chemistry, urinalysis, and chest x-ray.<br><br>The severity of adverse events will be graded according to NCI-CTCAE v5.0. Patients will be reviewed up to 30 days after the last administration of study drug to document any late adverse effects.<br><br>Discrete data will be compared using the Pearson`s chi-square test or Fisher's exact test. |

### 10.2.2 Efficacy analysis

| **Endpoint** | **Statistical Analysis Methods** |
|---|---|
| **Primary** | |
| Objective response rate | Response will be documented by physical examination prior to each treatment cycle and CT scan every 8 weeks subsequently.<br>Response will be assessed uni-dimensionally according to the RECIST 1.1.<br>Response rate for each treatment arm will be compared using the Pearson`s chi-square test or Fisher's exact test. |
| **Secondary** | |
| PFS | PFS is defined as a period from the start of study treatment to the progression according to the RECIST 1.1 or any cause of death, whichever occur first.<br>The Kaplan-Meier method and the log-rank test were used to estimate and to compare the survival distribution, respectively. |
| OS | OS is defined as a period from the initiation of treatment to any cause of death.<br>The Kaplan-Meier method and the log-rank test were used to estimate and to compare the survival distribution, respectively. |

### 10.2.3 Exploratory analyses

| **Subject** | **Parallel translational research plan** |
|---|---|
| Circulating tumor DNA analysis | Using baseline blood sample, commercially available platform will be used |
| RNA sequencing | Using PBMC samples before and after study treatment |
| FACS analysis of T-cell subpopulation | Using PBMC samples before and after study treatment |

## 10.3 Determination of sample size

In the previous phase III trials, sorafenib, current standard $1^{st}$ line therapy, showed the objective response rates (ORR) of 7% (P0) graded by RECIST v1.1. With the regorafenib-Nivolumab combination, we assumed that the partial response rates might be enhanced to 25% (P1). With alpha of two sided 0.05 and power of 90%, 35 patients are needed based on Fleming's single-stage Phase 2 design calculation. Considering 15% of follow-up loss rates, a total 42 patients are needed for this study.

# 11.0 STUDY MANAGEMENT

## 11.1 Training of study site personnel

The PI will ensure that appropriate training relevant to the study is given to all of these staff, and that any new information relevant to the performance of this study is forwarded to the staff involved.

The PI will maintain a record of all individuals involved in the study (medical, nursing and other staff).

## 11.2 Monitoring of the study

The PI will monitor the study

- To confirm that the investigational team is adhering to the protocol, that data are being accurately and timely recorded in the CRFs, that biological samples are handled in accordance with the Laboratory Manual and that study drug accountability checks are being performed.
- To perform source data verification (a comparison of the data in the CRFs with the patient's medical records at the hospital or practice, and other records relevant to the study) including verification of informed consent of participating patients. This will require direct access to all original records for each patient (e.g,, clinic charts)
- To ensure withdrawal of informed consent to the use of the patient's biological samples is reported and biological samples are identified and disposed of/destroyed accordingly, and the action is documented, and reported to the patient.

## 11.3 Timelines

| | Expected timelines |
|---|---|
| Enrolment rate (patients per month) | 9 patients per month |
| Planned estimate of treatment(s) duration | 14 months |
| Submission date to health authority/ethics | Q3 2019 |
| Start of subject enrolment | Q4 2019 |
| End of Subject enrolment | Q2 2021 |
| All patients completed | Q4 2022 |
| End of Study | Q1 2024 |
| Report (as described in the contract) | Q2 2024 |
| Planned publication/presentation for primary endpoint | Q2 2023 |

# 12. ETHICAL AND REGULATORY REQUIREMENTS

## 12.1 Ethical conduct of the study

The study will be performed in accordance with ethical principles that have their origin in the Declaration of Helsinki and are consistent with ICH/Good Clinical Practice, and applicable regulatory requirements Patient data protection.

## 12.2 Informed consent

The investigator or qualified designee must obtain documented consent from each potential subject or each subject's legally acceptable representative prior to participating in a clinical trial or Future Biomedical Research. If there are changes to the subject's status during the trial (e.g., health or age of majority requirements), the investigator or qualified designee must ensure the appropriate consent is in place.

## 12.3 Changes to the protocol and informed consent form

The initial informed consent form, any subsequent revised written informed consent form and any written information provided to the subject must receive the IRB/ERC's approval/favorable opinion in advance of use. The subject or his/her legally acceptable representative should be informed in a timely manner if new information becomes available that may be relevant to the subject's willingness to continue participation in the trial. The communication of this information will be provided and documented via a revised consent form or addendum to the original consent form that captures the subject's dated signature or by the subject's legally acceptable representative's dated signature.

Specifics about a trial and the trial population will be added to the consent form template at the protocol level.

The informed consent will adhere to IRB/ERC requirements, applicable laws and regulations and Sponsor requirements.

## 12.4 Audits and inspections

The investigator also agrees to allow monitoring, audits, IRB review and regulatory authority inspection of trial-related documents and procedures and provide for direct access to all trial-related source data and documents.

Trial documentation will be promptly and fully disclosed to the Sponsor by the investigator upon request and also shall be made available at the trial site upon request for inspection, copying, review and audit at reasonable times by representatives of the Sponsor or any regulatory authorities. The investigator agrees to promptly take any reasonable steps that are requested by the Sponsor as a result of an audit to cure deficiencies in the trial documentation and worksheets/case report forms.

## 12.5 Confidentiality

### 12.5.1 Confidentiality of Data

By signing this protocol, the investigator affirms to the investigator that information furnished will be maintained in confidence, and such information will be divulged to the institutional review board, ethics review committee (IRB) or similar or expert committee; affiliated institution and employees, only under

an appropriate understanding of confidentiality with such board or committee, affiliated institution and employees. Data generated by this trial will be considered confidential by the investigator, except to the extent that it is included in a publication as provided in the Publications section of this protocol.

### 12.5.1 Confidentiality of Subject Records

By signing this protocol, the investigator agrees that IRB, or regulatory authority representatives may consult trial documents in order to verify worksheet/case report form data. By signing the consent form, the subject agrees to this process. If trial documents will be photocopied during the process of verifying worksheet/case report form information, the subject will be identified by unique code only

By signing this protocol, the investigator agrees to treat all subject data used and disclosed in connection with this trial in accordance with all applicable privacy laws, rules and regulations.

## 13.0 DATA MANAGEMENT

The investigator or qualified designee is responsible for recording and verifying the accuracy of subject data. By signing this protocol, the investigator acknowledges that his/her electronic signature is the legally binding equivalent of a written signature. By entering his/her electronic signature, the investigator confirms that all recorded data have been verified as accurate.

To enable evaluations and/or audits from regulatory authorities, the investigator agrees to keep records, including the identity of all participating subjects (sufficient information to link records, eg, CRFs and hospital records), all original signed informed consent forms, copies of all CRFs, source documents, and detailed records of treatment disposition. The records should be retained by the Investigator according to ICH, local regulations, or as specified in the Clinical Study Agreement, whichever is longer.

# APPENDIX

## Appendix 1. ECOG Performance Status Scores

| Description | Status |
|---|---|
| Fully active, able to carry on all pre-disease performance without restriction. | 0 |
| Restricted in physically strenuous activity but ambulatory and able to carry out work of a light or sedentary nature, e.g., light housework, office work. | 1 |
| Ambulatory and capable of all self-care but unable to carry out any work activities. | 2 |
| Capable of only limited self-care, confined to bed or chair more than 50% of waking hours. | 3 |
| Completely disabled. Cannot carry on any self-care. Totally confined to bed or chair. | 4 |
| Dead | 5 |

## Appendix 2. Common Terminology Criteria for Adverse Events V5.0 (CTCAE)

The description and grading scales found in the CTEP Version 5 of the NCI Common Terminology Criteria for Adverse Events (CTCAE) will be utilized for AE reporting. CTEP Version 5 of the CTCAE is identified and located at:

https://ctep.cancer.gov/protocolDevelopment/electronic_applications/ctc.htm

## Appendix 3. Response evaluation criteria in solid tumors (RECIST) version 1.1

https://ctep.cancer.gov/protocoldevelopment/docs/recist_guideline.pdf

## Appendix 4. Schedule of Study Procedures: Follow-Up for Patients Who Have Discontinued Study Treatment Due to Progression of Disease or unacceptable toxicities

| **Evaluation** | **Time Since Last Dose of IP** | | | | | | | |
|---|---|---|---|---|---|---|---|---|
| | **Day (±3)** | **Months (±1 week)** | | | | | | **12 Months and Every 3 Months (±2 weeks)** |
| | **30** | **2** | **3** | **4** | **6** | **8** | **10** | |
| Physical examination | X | | | | | | | |
| Vital signs (temperature, respiratory rate, blood pressure, pulse) | X | | | | | | | |
| Weight | X | | | | | | | |
| AE/SAE assessment | X | | | | | | | |
| Concomitant medications | X | | | | | | | |
| ECOG performance status | X | | | | | | | |
| Subsequent anticancer therapy | X | X | X | X | X | X | X | X |

| Evaluation | Time Since Last Dose of IP | | | | | | | |
|---|---|---|---|---|---|---|---|---|
| | Day (±3) | Months (±1 week) | | | | | | 12 Months and Every 3 Months (±2 weeks) |
| | 30 | 2 | 3 | 4 | 6 | 8 | 10 | |
| Survival status: phone contact with patients who refuse to return for evaluations and agree to be contacted | X | X (every 3 months) | | | | | | X (every 3 months) |
| Hematology | X | | | | | | | |
| Serum chemistry | X | | | | | | | |

# Statistical Analysis Plan

| | |
|---|---|
| **Sponsor** | Asan Medical Center |
| **Study Title** | Phase II study of regorafenib-nivolumab combination therapy for chemotherapy-naïve patients with unresectable or metastatic hepatocellular carcinoma |
| **Protocol No.** | RENOBATE |
| **Version** | v1.0 |

| | | | | | |
|---|---|---|---|---|---|
| **Prepared by:** | | | | | |
| **Name:** | Jiyoung Jung | **Signature:** | | **Date:** | |
| **Title:** | Sr. Biostatistician | | **Department:** | SS Dept, SA team | |
| **Confirmed by CMIC Korea:** | | | | | |
| **Name:** | Keumna Hwang | **Signature:** | | **Date:** | |
| **Title:** | Sr. SA Manager | | **Department:** | SS Dept, SA team | |
| **Approved by Sponsor:** | | | | | |
| **Name:** | Changhoon Yoo | **Signature:** | | **Date:** | |
| **Title:** | Assistant Professor | | **Department:** | Department of Oncology, Asan Medical Center | |

## Version Information

| Version/Notation | Prepared | Details |
|---|---|---|
| 1.0 | Jiyoung Jung | Newly created |

# Abbreviation

| Term | Definition |
|---|---|
| AASLD | American Association for the Study of Liver Diseases |
| AE | Adverse Event |
| aPTT | activated Partial Thromboplastin Time |
| BCLC | Barcelona Clinic Liver Cancer |
| CNS | Central nervous system |
| COPD | Chronic obstructive pulmonary disease |
| CR | Complete remission |
| CxDx | Cycle x Day x |
| ECOG | Eastern Cooperative Oncology Group |
| FACS | Fluorescence activated cell sorting |
| HBV | Hepatitis B virus |
| HCC | Hepatocellular carcinoma |
| HCV | Hepatitis C virus |
| HIV | Human immunodeficiency virus |
| INR | International normalized ratio |
| MedDRA | Medical Dictionary for Regulatory Activities |
| ORR | Objective response rate |
| OS | Overall survival |
| PD | Progressive disease |
| PD-1 | Programmed cell death 1 |
| PFS | Progressive free survival |
| PT | Preferred Term |
| PR | Partial remission |
| RECIST | Response evaluation criteria in solid tumors |
| SAE | Serious adverse event |
| SOC | System Organ Class |

# Table of Contents


1. **Background** .... 6
2. **Duties, Responsibilities and Roles** .... 6
   - 2.1 **Duties** .... 6
   - 2.2 **Responsibilities and Roles** .... 6
3. **Study Design** .... 6
   - 3.1 **Study Design** .... 6
   - 3.2 **Objectives** .... 6
     - 3.2.1 **Primary Objective** .... 6
     - 3.2.2 **Secondary Objectives** .... 6
     - 3.2.3 **Exploratory Objectives** .... 7
   - 3.3 **Target Disease** .... 7
   - 3.4 **Inclusion and Exclusion Criteria** .... 7
     - 3.4.1 **Inclusion Criteria** .... 7
     - 3.4.2 **Exclusion Criteria** .... 8
   - 3.5 **Investigational Products** .... 11
4. **Clinical Trial Procedure and Process** .... 12
   - 4.1 **Trial Procedure** .... 12
5. **Target Patient Number** .... 12
   - 5.1 **Determination of Sample Size** .... 12
   - 5.2 **Rationale for Sample Size Calculation** .... 13
6. **Analysis Set** .... 13
   - 6.1 **Efficacy Analysis Set** .... 13
   - 6.2 **Safety Analysis Set** .... 13
   - 6.3 **Exploratory Biomarker Analysis Set** .... 13
7. **Study Endpoints** .... 13
   - 7.1 **Primary Efficacy Endpoint** .... 13
   - 7.2 **Secondary Efficacy Endpoints** .... 13
   - 7.3 **Safety Profile Variables** .... 13
   - 7.4 **Exploratory Study Endpoints** .... 14
8. **Statistical Analysis Method** .... 14
   - 8.1 **General Principles of Statistical Analysis** .... 14
     - 8.1.1 General Considerations for Statistical Analysis .... 14
     - 8.1.2 Handling of Missing Data and Missing Values .... 14
     - 8.1.3 Adjustment for Covariates .... 14
     - 8.1.4 Multiple Comparisons or Multiplicity .... 14
     - 8.1.5 Format of Data Presentation .... 14
     - 8.1.6 Format for Evaluation Time Points .... 14

8.1.7 Software ... 15
8.1.8 Dictionaries ... 15
**8.2 Evaluation of Clinical Trial Participation** ... 15
8.2.1 Clinical Trial Participation Status ... 16
8.2.2 Clinical Trial Participation Status of Subjects by Participating Institution ... 16
8.2.3 Dropout ... 16
8.2.4 Detailed Information on Subjects Excluded from the Analysis Group ... 16
**8.3 Demographic Information and Baseline Characteristics** ... 16
8.3.1 Demographic Information and Baseline Characteristics ... 16
8.3.2 History of Liver Cancer ... 17
8.3.3 History of liver cancer treatment ... 17
8.3.4 Past Medical History & Current Medical History ... 18
8.3.5 Prior/Concomitant Medication ... 18
8.3.6 Administration Status of the Investigational Drug ... 18
**8.4 Efficacy Evaluation** ... 19
8.4.1 Evaluation of Primary Endpoint ... 19
8.4.1 Evaluation of Secondary Endpoints ... 20
**8.5 Safety Analysis** ... 20
8.5.1 Adverse Reaction ... 20
8.5.2 Vital sign ... 21
8.5.3 Physical Examination ... 21
8.5.4 Clinical Laboratory Tests ... 21
8.5.5 ECOG performance status analysis ... 22
**9. Appendix** ... 22
**10. Reference** ... 23
**11. List of Applicable SOPs** ... 23

# 1. Background

This SAP (Statistical Analysis Plan) details the statistical analysis plan for the clinical trial protocol RENOBATE, which is a 'Phase II study of regorafenib-nivolumab combination therapy for chemotherapy-naïve patients with unresectable or metastatic hepatocellular carcinoma'.

# 2. Duties, Responsibilities and Roles

## 2.1 Duties

| No | Scope | CMIC | Sponsor | Assigned person | Comments |
|---|---|---|---|---|---|
| 1 | Statistical Analysis Plan | ☒ | ☐ | Biostatistician | |
| 2 | Statistical Analysis | ☒ | ☐ | Biostatistician | |
| 3 | Statistical Analysis Result | ☒ | ☐ | Biostatistician | |

## 2.2 Responsibilities and Roles

| Role | Responsibility | Comments |
|---|---|---|
| Project Biostatistician | Statistical Analysis | |
| SA Team Leader | QC of Statistical Analysis | |
| Sponsor | Approval of Statistical Analysis Plan and Statistical Analysis Result | |

# 3. Study Design

## 3.1 Study Design

This study is a phase II, open-label, non-blinding, non-comparative, multicenter trial for patients with advanced HCC.

## 3.2 Objectives

### 3.2.1 Primary Objective

To evaluate the objective response rates (defined by RECIST v1.1) of regorafenib-nivolumab combination in unresectable HCC patients

### 3.2.2 Secondary Objectives

To evaluate PFS, OS, safety profile and modified RECIST with regorafenib-nivolumab combination in patients with unresectable HCC

### 3.2.3 Exploratory Objectives

To identify biomarkers of regorafenib-nivolumab combination for clinical outcomes in patients with HCC using Circulating tumor DNA, single cell RNA sequencing and Fluorescence activated cell sorting (FACS) analysis.

## 3.3 Target Disease

Unresectable or metastatic HCC

## 3.4 Inclusion and Exclusion Criteria

### 3.4.1 Inclusion Criteria

Having provided written consent before participation in the study, patients must fulfill all of the following criteria:

1. Age ≥ 19 years at time of signing Informed Consent Form
2. Ability to comply with the study protocol, in the investigator's judgment
3. HCC that was histologically/cytologically confirmed or clinically diagnosed by AASLD criteria in cirrhotic patients. Patients without liver cirrhosis require histological confirmation of HCC
4. Locally advanced unresectable or metastatic disease that is not amenable to curative surgical a nd/or locoregional therapies, or that progressed after surgical and/or locoregional therapies
5. No prior systemic therapy for HCC
6. At least one measurable (per RECIST 1.1) lesion as confirmed by imaging within 28 days prior to initiation of study treatment
7. Patients who received prior local therapy (e.g., radiofrequency ablation, percutaneous ethanol or acetic acid injection, cryoablation, high-intensity focused ultrasound, transarterial chemoemb olization, transarterial embolization, etc.) are eligible provided that other target lesion(s) have n ot been previously treated with local therapy or the target lesion(s) within the field of local thera py have subsequently progressed in accordance with RECIST 1.1.
8. Pre-treatment tumor tissue sample (if available)
   - If tumor tissue is available, approximately 10–30 slides containing unstained, freshly cut, serial sections will be required subsequently for translational research.
   - If tumor tissue is not available (e.g., depleted because of prior diagnostic testing), patients are still eligible.
9. ECOG Performance Status score 0 or 1
10. Child-Pugh class A
11. Adequate hematologic and end-organ function, defined by the following laboratory test results, obtained within 14 days prior to initiation of study treatment, unless otherwise specified:
    - ANC ≥ $1.0 \times 10^9$/L (1000/μL) without granulocyte colony-stimulating factor support
    - Platelet count ≥ $75 \times 10^9$/L (75,000/μL) without transfusion

- Hemoglobin ≥ 90 g/L (9 g/dL): Patients may be transfused to meet this criterion.
- AST, ALT, and alkaline phosphatase (ALP) ≤ 3 × upper limit of normal (ULN)
- Serum bilirubin ≤ 2× ULN
- Serum creatinine ≤ 1.5 × ULN or creatinine clearance ≥ 50 mL/min (calculated using the Cockcroft-Gault formula)
- Serum albumin ≥ 28 g/L (2.8 g/dL)
- For patients not receiving therapeutic anticoagulation: INR or aPTT ≤ 2× ULN
- Urine dipstick for proteinuria < 2+
- Patients discovered to have ≥ 2+ proteinuria on dipstick urinalysis at baseline should undergo a 24-hour urine collection and must demonstrate < 1 g of protein in 24 hours.

12. Resolution of any acute, clinically significant treatment-related toxicity from prior therapy to Gra de ≤ 1 prior to study entry, with the exception of alopecia
13. Negative HIV result at screening test or prior tested conducted within 3 years
14. Documented virology status of hepatitis, as confirmed by screening HBV and HCV serology tes t
    - Patients with active hepatitis B virus (HBV) must meet the followings: HBV DNA < 500 IU/mL obtained within 14 days prior to initiation of study treatment, anti-HBV treatment (per local standard of care; e.g., entecavir) for a minimum of 14 days prior to study entry and willingness to continue treatment for the length of the study
15. Women of childbearing potential (including women with chemical menopause or no menstruatio n for other medical reasons)#1 must agree to use contraception#2 from the time of informed con sent until 5 months or more after the last dose of the investigational product. Also, women must agree not to breastfeed from the time of informed consent until 5 months or more after the last dose of the investigational product.
16. Men must agree to use contraception#2 from the start of study treatment until 7 months or more after the last done of the investigational product.

#1 Women of childbearing potential are defined as all women after the onset of menstruation who are not postmenopausal and have not been surgically sterilized (e.g., hysterectomy, bilateral tubal ligation, bilateral oophorectomy). Postmenopause is defined as amenorrhea for ≥12 consecutive months without specific reasons. Women using oral contraceptives, intrauterine devices, or mechanical contraception such as contraceptive barriers are regarded as having childbearing potential.

#2 The subject must consent to use any two of the following methods of contraception: vasectomy or condom for patients who are male or female subject's partner and tubal ligation, contraceptive diaphragm, intrauterine device, spermicide, or oral contraceptive for patients who are female or male subject's partner.

### 3.4.2 Exclusion Criteria

Patients who meet any of the following criteria at the time of screening will be excluded. If a subject is found to meet any of the following criteria before the first dose of the investigational product, the subject will not be started on the study treatment and will be withdrawn from the study.

1. Patients who are diagnosed with fibrolamellar HCC, sarcomatoid HCC, or combined type of cholangiocarcinoma and HCC
2. Patients with a history of malignancy other than HCC within 3 years prior to screening, with the exception of malignancies with a negligible risk of metastasis or death (e.g., 5-year OS rate > 90%), such as adequately treated carcinoma in situ of the cervix, non-melanoma skin carcinoma, localized prostate cancer, ductal carcinoma in situ, and Stage I uterine cancer
3. Patients with a history of leptomeningeal seeding
4. Patients with symptomatic, untreated, or actively progressing central nervous system (CNS) metastases.
   - Asymptomatic patients with treated CNS lesions are eligible, provided that all of the following criteria are met:
     (1) The patients must have at least one measurable lesion, per RECIST 1.1, other than CNS metastases
     (2) The patient must not have a history of intracranial hemorrhage or spinal cord hemorrhage
     (3) The metastatic lesions have to be limited in cerebellum or supratentorial region (e.g., not to the midbrain, pons, medulla, or spinal cord)
     (4) There must be no evidence of interim progression between the completion of CNS-directed therapy and initiation of the study treatment
     (5) The patient must not undergo stereotactic radiotherapy within 7 days, whole-brain radiotherapy within 14 days, or neurosurgical resection within 28 days prior to initiation of the study treatment
     (6) The patient must not have ongoing requirement for corticosteroids for CNS disease
   - Anticonvulsant therapy at a stable dose is permitted.
   - Asymptomatic patients with CNS metastases newly detected at screening are eligible for the study after receiving radiotherapy or surgery, with no need to repeat the screening brain scan.
5. Patients with current of past history of autoimmune disease or immunodeficient disease (including, but not limited to, myasthenia gravis, myositis, autoimmune hepatitis, systemic lupus erythematosus, rheumatoid arthritis, inflammatory bowel disease, antiphospholipid antibody syndrome, Wegener granulomatosis, Sjögren syndrome, Guillain-Barré syndrome, or multiple sclerosis) with the following exceptions:
   - Patients with autoimmune-related hypothyroidism who are on thyroid-replacement hormone are eligible.
   - Patients with controlled Type 1 diabetes mellitus who are on an insulin regimen are eligible.
   - Patients with eczema, psoriasis, lichen simplex chronicus, or vitiligo with dermatologic manifestations only (e.g., patients with psoriatic arthritis are excluded) are eligible for the study provided all of following conditions are met:
     (1) Rash must cover < 10% of body surface area
     (2) Disease has to be well controlled at baseline and requires only low-potency topical corticosteroids
     (3) There must be no occurrence of acute exacerbations of the underlying condition requiring psoralen plus ultraviolet A radiation, methotrexate, retinoids, biologic agents,

oral calcineurin inhibitors, or high-potency or oral corticosteroids within the previous 12 months

6. Patients with current or past history of idiopathic pulmonary fibrosis, organizing pneumonia (e. g., bronchiolitis obliterans), drug-induced pneumonitis, or idiopathic pneumonitis, or evidence of active pneumonitis on screening chest computed tomography (CT) scan.
   - Patients with history of radiation pneumonitis in the radiation field (fibrosis) are eligible if the radiation pneumonitis has been confirmed as stable (beyond acute phase) without any concerns about recurrence.
7. Patients who have experienced a transient ischemic attack, cerebrovascular accident, thrombo sis, or thromboembolism (pulmonary arterial embolism or deep vein thrombosis) within 6 month s before initiation of study treatment
8. Patients with a history of uncontrollable or significant cardiovascular disease meeting any of the following criteria:
   - Myocardial infarction within 6 months before initiation of study treatment
   - Uncontrollable angina pectoris within 6 months before initiation of study treatment
   - New York Heart Association Class II or greater congestive heart failure within 6 months before initiation of study treatment
   - Uncontrollable hypertension despite appropriate treatment (e.g., systolic blood pressure ≥150 mmHg or diastolic blood pressure > 90 mmHg based on an average of ≥ 3 BP readings on ≥ 2 sessions)
   - Arrhythmia requiring treatment
9. Patients with congenital long QT syndrome or corrected QT interval > 450 ms (calculated with u se of the Fridericia method) at screening
10. Patients with systemic infections (including active tuberculosis) requiring treatment
11. Patients with history of hypertensive crisis or hypertensive encephalopathy
12. Patients with significant vascular disease (e.g., aortic aneurysm requiring surgical repair or rece nt peripheral arterial thrombosis) within 6 months prior to initiation of study treatment
13. Patients who underwent major surgical procedure, other than for diagnosis, within 4 weeks prio r to initiation of study treatment or who are expected to need a major surgical procedure during the study
14. Patients who have received radiotherapy within 28 days before initiation, or radiotherapy to bon e metastases within 14 days before initiation
15. Patients with prior history of allogeneic stem cell or solid organ transplantation
16. Patients with current or past history of severe allergic anaphylactic reactions to chimeric or hum anized antibodies or fusion proteins
17. Patients with untreated or incompletely treated varices with active bleeding or high risk for blee ding
18. Patients with moderate or severe ascites
19. Patients with history of hepatic encephalopathy
20. Patients with evidence of bleeding diathesis or significant coagulopathy (in the absence of ther apeutic anticoagulation)
21. Patients who had recent (within 10 days of first dose of study treatment) use of aspirin (> 300 m g/day) or treatment with dipyramidole, ticlopidine, clopidogrel, and cilostazol
22. Patients who had recent use of full-dose oral or parenteral anticoagulants or thrombolytic agent s for therapeutic (as opposed to prophylactic) purpose

- Prophylactic anticoagulation for the patency of venous access devices is allowed provided the activity of the agent results in an INR < 1.5 × ULN and aPTT within normal limits within 14 days prior to initiation of study treatment.
- Prophylactic use of low molecular-weight heparin (i.e., enoxaparin 40 mg/day) is allowed.

23. Patients who treated with strong CYP3A4 inducers within 14 days prior to initiation of study trea tment, including rifampin (and its analogues) or St. John's wort
24. Patients who have previously received CD137 agonists or immune checkpoint blockade therapi es, including anti–CTLA-4, anti–PD-1, and anti–PD-L1 therapeutic antibodies
25. Patients who were treated with systemic immunostimulatory agents (including, but not limited t o, interferon and interleukin 2 [IL-2]) within 4 weeks or 5 half-lives of the drug (whichever is long er) prior to initiation of study treatment
26. Patients who were treated with systemic immunosuppressive medication (including, but not limi ted to, corticosteroids, cyclophosphamide, azathioprine, methotrexate, thalidomide, and anti–T NF-α agents) within 2 weeks prior to initiation of study treatment, or anticipation of need for sys temic immunosuppressive medication during study treatment, with the following exceptions:
    - Patients who received temporary, low-dose systemic immunosuppressant medication or a one-time pulse dose of systemic immunosuppressant medication (e.g., 48 hours of corticosteroids for a contrast allergy) are eligible for the study.
    - Patients who received mineralocorticoids (e.g., fludrocortisone), or corticosteroids for chronic obstructive pulmonary disease (COPD) or asthma, or low-dose corticosteroids for orthostatic hypotension or adrenal insufficiency are eligible for the study.
27. Patients who had abdominal or tracheoesophageal fistula, gastrointestinal (GI) perforation, or i ntra-abdominal abscess within 6 months prior to initiation of study treatment
28. Patients who had intestinal obstruction and/or clinical signs or symptoms of GI obstruction inclu ding sub-occlusive disease related to the underlying disease or requirement for routine parente ral hydration, parenteral nutrition, or tube feeding within 6 months prior to initiation of study trea tment
    - Patients with signs/symptoms of sub-/occlusive syndrome/intestinal obstruction at time of initial diagnosis may be enrolled if they had received definitive (surgical) treatment for symptom resolution.
29. Women who are pregnant or breastfeeding, or possibly pregnant
30. Other patients judged by the investigator or sub-investigator to be inappropriate as subjects of t his study

### 3.5 Investigational Products

1) Nivolumab

| | |
|---|---|
| Product name/dose | Nivolumab (OPDIVO®), 100mg/10mL (10mg/mL)/vial |
| Formulation and packaging | Clear to opalescent, colorless to pale-yellow solution |
| Storage | A secure, limited-access location under the storage conditions specified on the label (2 ~ 8℃) |
| Administration | Intravenous |

2) Regorafenib

| | |
|---|---|
| Product name/dose | Regorafenib (Stivarga®), 40mg/tab |
| Formulation and packaging | light pink, oval shaped, film-coated tablet |
| Storage | Drug bottle between 15 ~ 30℃ |
| Administration | Oral |

## 4. Clinical Trial Procedure and Process

### 4.1 Trial Procedure

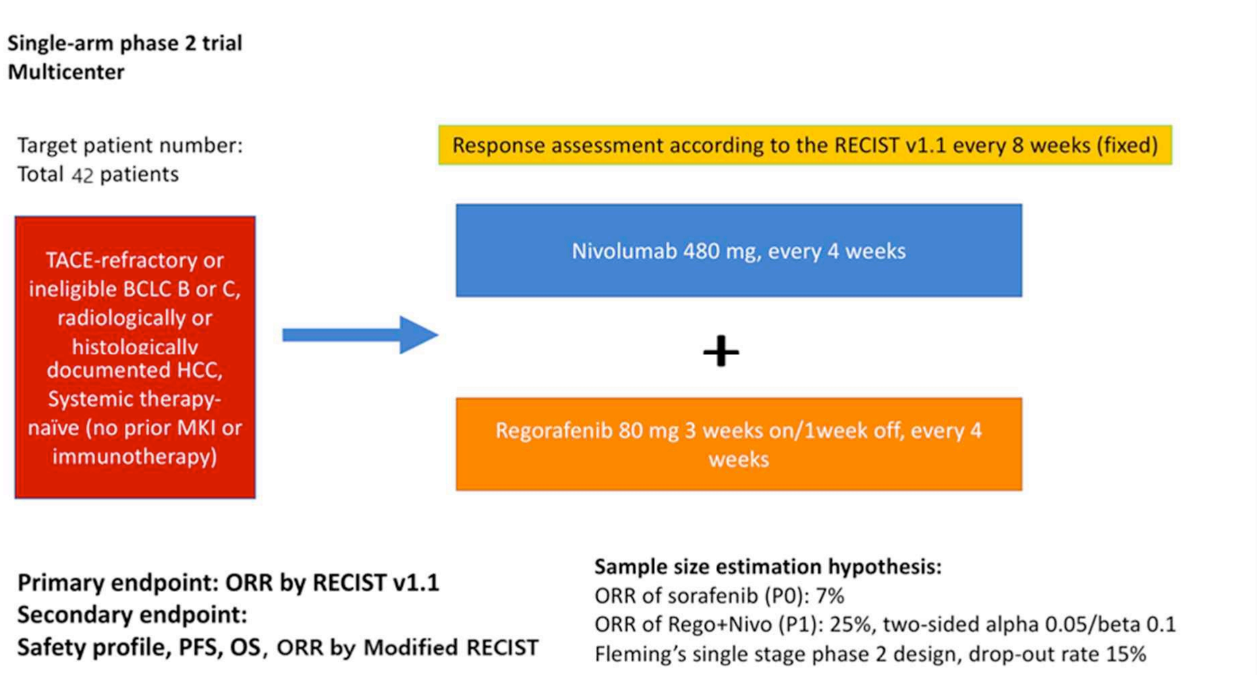


All eligible patients will be treated with nivolumab 480 mg intravenous at day 1 and regorafenib 80 mg at days 1-21, every 4 weeks.

Tumor response will be performed every 8 weeks and graded per RECIST 1.1

## 5. Target Patient Number

### 5.1 Determination of Sample Size

With alpha of two sided 0.05 and power of 90%, 35 patients are needed based on Fleming's single-stage Phase 2 design calculation. Considering 15% of follow-up loss rates, a total 42 patients are needed for this study.

### 5.2 Rationale for Sample Size Calculation

In the previous phase III trials, sorafenib, current standard 1st line therapy, showed the objective response rates (ORR) of 7% (P0) graded by RECIST v1.1. With the regorafenib-Nivolumab combination, we assumed that the partial response rates might be enhanced to 25% (P1).

## 6. Analysis Set

### 6.1 Efficacy Analysis Set

Efficacy analysis will be performed on the intent-to-treat (ITT) population. All participants will be candidates for efficacy analysis.

### 6.2 Safety Analysis Set

All participants, who were administered any dose of any study intervention will be candidates for safety analysis. Analyses will consider these participants as treated.

### 6.3 Exploratory Biomarker Analysis Set

Patient subgroup for the correlative biomarker analyses using circulating DNA, single cell RNA sequencing and FACS analysis will be defined after the primary analysis for the clinical outcomes considering the unexpected nature of clinical outcomes for novel therapeutic regimen.

## 7. Study Endpoints

### 7.1 Primary Efficacy Endpoint

- Objective response rate (ORR) per RECIST 1.1 (Proportion of complete response [CR] and partial response [PR])

### 7.2 Secondary Efficacy Endpoints

- Progressive free survival (PFS)
- Overall survival (OS)
- ORR per modified RECIST

### 7.3 Safety Profile Variables

- Adverse events
- Vital sign
- Physical examination
- Laboratory evaluation
- ECOG performance status

### 7.4 Exploratory Study Endpoints

- Circulating tumor DNA
- Single cell RNA sequencing
- Fluorescence activated cell sorting (FACS) analysis

## 8. Statistical Analysis Method

### 8.1 General Principles of Statistical Analysis

#### 8.1.1 General Considerations for Statistical Analysis

For continuous data in this clinical trial, descriptive statistics such as the number of test subjects, mean, standard deviation, median, minimum, and maximum values are provided. For categorical data, frequencies and percentages (%) are presented. All statistical analyses are performed with a two-sided test at a significance level of 5%.

#### 8.1.2 Handling of Missing Data and Missing Values

Missing values are not replaced with other values.

#### 8.1.3 Adjustment for Covariates

Adjustment for covariates is not considered in this clinical trial.

#### 8.1.4 Multiple Comparisons or Multiplicity

Multiple comparisons or multiplicity are not considered in this clinical trial

#### 8.1.5 Format of Data Presentation

Mean, standard deviation, median, minimum, maximum values, ratios, and confidence intervals are rounded to two decimal places.

#### 8.1.6 Format for Evaluation Time Points

Efficacy or safety evaluation variables are evaluated considering a 4-week treatment period as one cycle based on Screening and C1D1. The summary of each evaluation variable by time point will be presented according to regular visits as follows.

| Visit | Analysis Label | Actual Point | Visit window |
|---|---|---|---|
| Screening | Baseline | -Week 8 ~ Week 0 | - week 4 ~ - day 1 |
| C1D1 | C1D1 | Week 0 | |
| C1D15 | C1D15 | C1D1 + week 2 | ± day 3 |
| C2D1 | C2D1 | C1D1 + week 4 | ± day 3 |
| C2D15 | C2D15 | C1D1 + week 6 | ± day 3 |
| C3D1 | C3D1 | C1D1 + week 8 | ± day 3 |
| C3D15 | C3D15 | C1D1 + week 10 | ± day 3 |
| C4D1 | C4D1 | C1D1 + week 12 | ± day 3 |
| CkD1 | CkD1 | C1D1 + (k-1) X week 4 | ± day 3 |
| End of Treatment | EOT | Assessment taken at the end of treatment visit | |

The time point before drug administration (Baseline) in statistical analysis is defined as follows.

1) Safety Evaluation Items
    - Vital Signs: C1D1 (Before IP administration)
    - Physical Examination: C1D1 (Before IP administration)
    - Clinical Laboratory Test: C1D1 (Before IP administration)
    - ECOG Performance Status: C1D1 (Before IP administration)
    - Electrocardiogram Test: Screening

#### 8.1.7 Software

All statistical analysis will be conducted using SAS (Statistical Analysis System) over 9.4 version..

#### 8.1.8 Dictionaries

1) Past and current medical history will be standardized in English using MedDRA (the latest version) according to the System Organ Class (SOC) and Preferred Term (PT).
2) Adverse reactions will be standardized in English using MedDRA (the latest version) according to the System Organ Class (SOC) and Preferred Term (PT).
3) Concomitant and prior medications will be standardized in English using the ATC Index Code (the latest version) based on the anatomical category (level 1) and therapeutic category (level 2).
4) When preparing the Statistical Analysis Report (SAR), the dictionary version used must be clearly specified.

### 8.2 Evaluation of Clinical Trial Participation

#### 8.2.1 Clinical Trial Participation Status

The clinical trial participation status is summarized as follows and presented in tables and diagrams. In the case of diagrams, only the number of subjects is presented. The ratio is calculated based on the number of subjects registered for the clinical trial.

- Number of subjects for screening, screening dropout, and reasons for screening dropout
- Number of subjects registered for the clinical trial
- Number and ratio of subjects who completed the clinical trial, dropped out midway, and reasons for dropping out midway
- Number and ratio of subjects in the safety analysis group, excluded from the safety analysis group, and reasons for exclusion
- Number and ratio of subjects in the efficacy evaluation group, excluded from the efficacy evaluation group, and reasons for exclusion.

#### 8.2.2 Clinical Trial Participation Status of Subjects by Participating Institution

The number and ratio of subjects' clinical trial participation status are presented according to the test institution.

- Clinical trial participating institution, screening, clinical trial registration, clinical trial completion, safety analysis group, efficacy evaluation group

#### 8.2.3 Dropout

The detailed reasons for the dropout of the subjects who dropped out midway are presented.

- Screening number, safety analysis group, efficacy evaluation group, gender, age, reason for dropout

#### 8.2.4 Detailed Information on Subjects Excluded from the Analysis Group

The detailed reasons for the exclusion of subjects from each analysis group are presented by the administered group.

- Analysis group, screening number, gender, age, reason for exclusion

### 8.3 Demographic Information and Baseline Characteristics

Demographic information and characteristics prior to administration are presented for the efficacy evaluation group.

#### 8.3.1 Demographic Information and Baseline Characteristics

For continuous variables related to demographic information and baseline characteristics, descriptive statistics (number of subjects, mean, standard deviation, median, minimum, maximum) are presented. For categorical variables, frequency and ratio are presented.

• Continuous data: Age (years), height (cm), weight (kg), BMI (kg/m^2)

• Categorical data: Gender, age group (19-29 years, 30-39 years, 40-49 years, 50-59 years, 60-69 years, 70 years and above)

• Derived data:

BMI (kg/$m^2$) = weight (kg) / (height (cm)/100)$^2$

### 8.3.2 History of Liver Cancer

For continuous variables related to the history of liver cancer, descriptive statistics (number of subjects, mean, standard deviation, median, minimum, maximum) are presented. For categorical variables, frequency and ratio are presented.

- Continuous data: Duration of illness (days)
- Categorical data: Diagnostic method, disease status at the time of screening, presence of liver cirrhosis, BCLC stage, presence of hepatitis B, presence of hepatitis C, presence of fatty liver, presence and location of extrahepatic spread
- Derived data:
    - Duration of illness (days) = Date of consent signing - Date of diagnosis
    - Presence of Hepatitis B: If 'Hepatitis B' is collected as 'Y', then 'Yes', otherwise 'No'
    - Presence of Hepatitis C: If 'Hepatitis C' is collected as 'Y', then 'Yes', otherwise 'No'
    - Presence of Fatty liver: If 'Non-alcoholic (NASH)' is collected as 'Y' or 'Alcoholic' is collected as 'Y', then 'Yes', otherwise 'No'
    - Presence of extrahepatic spread - Others: If one or more of the 'Extrahepatic spread-Others' or 'Metastatic site_Others' items are 'Yes', then 'Yes', if all are 'No', then 'No'
    - Presence of extrahepatic spread: If one or more of the detailed extrahepatic spread items (central nervous system, lungs, bones, peritoneal seeding, lymph nodes, others) are 'Yes', then 'Yes', if all are 'No', then 'No'

### 8.3.3 History of liver cancer treatment

Regarding the history of liver cancer treatment, present the frequency and percentage (%) for whether liver cancer treatment was received, whether liver cancer surgery was performed, whether radiation therapy was received, and whether local treatment was received.

• Data derived:

Liver cancer treatment status: If there is a history of any of the treatments such as liver cancer surgery/radiation therapy/local treatment, the status of liver cancer treatment is 'Yes'. If none are present, it's 'No'.

### 8.3.4 Past Medical History & Current Medical History

Medical history is analyzed by dividing it into past medical history and current medical history. Present the frequency and percentage for the presence or absence of past and current medical history. All past and current medical histories are presented based on the System Organ Class (SOC) and Preferred Term (PT), showing the number of test subjects, percentage, and count.

The distinction between past medical history and current medical history is made based on the end date of the medical history and its ongoing status at the time of the visit.

- Past Medical History: If the end date is 'before the screening visit'.
- Current Medical History: If the end date is 'after the screening visit' or if the ongoing status of the medical history is marked as 'ongoing'.

### 8.3.5 Prior/Concomitant Medication

Medication administration history is analyzed by dividing it into prior medication and concomitant medication. Present the frequency and percentage for the administration history of prior and concomitant medications. Both prior and concomitant medications are presented based on the anatomical category (level 1) and therapeutic category (level 2), showing the number of test subjects, percentage, and count.

The distinction between prior and concomitant medications is made based on the end date of administration and its ongoing status. If it's difficult to distinguish between prior and concomitant medications, they are included as concomitant medications.

- Prior Medication: If the end date of administration is 'before the start date of the investigational drug administration'.
- Concomitant Medication: If the end date of administration is 'after the start date of the investigational drug administration' or if the ongoing status of administration is marked as 'ongoing'.

### 8.3.6 Administration Status of the Investigational Drug

Present descriptive statistics (number of test subjects, average, standard deviation, median, minimum, maximum) for the total cycles and the total dosage of both Regorafenib and Nivolumab. Also, provide the frequency and percentage for the status of cycle delays, as well as dose reduction and dose adjustment for both Regorafenib and Nivolumab.

• Total Cycles: The cycle at the last administration point.

• Total Dosage (mg) = Σ(dosage)

• Cycle Delayed: If the status of cycle delay is marked as 'Yes' at least once, it's considered 'Yes'. If all are marked 'No', it's considered 'No'.

• Dose Reduction:

- Regorafenib: If the dose status is marked as 'Reduced' or 'Discontinuation' at least once, it's considered 'Yes'. If all are marked 'No Adjustment', it's considered 'No'.

- Nivolumab: If the dose status is marked as 'Discontinuation' at least once, it's considered 'Yes'. If all are marked 'No Adjustment', it's considered 'No'.

• Dose Adjustment:

- Regorafenib: If the status of cycle delay is marked as 'Yes' at least once, or if the dose status is marked as 'Reduced' or 'Discontinuation' at least once, it's considered 'Yes'. Otherwise, it's considered 'No'.

- Nivolumab: If the status of cycle delay is marked as 'Yes' at least once, or if the dose status is marked as 'Discontinuation' at least once, it's considered 'Yes'. Otherwise, it's considered 'No'.

## 8.4 Efficacy Evaluation

The efficacy evaluation is presented for the ITT population.

### 8.4.1 Evaluation of Primary Endpoint

Provide the number and percentage of subjects who showed an objective response in the Best Overall response evaluated by RECIST 1.1, along with the 95% exact confidence interval.

• Objective Response Rates: Proportion of Complete Response (CR) or Partial Response (PR).

Additionally, the percentage change (% change) in the sum of the diameters of the target lesions was calculated and presented in the Waterfall plot and Spider plot for each subject.

- Percentage change rate (% change) =

$$\frac{(sum\ of\ target\ lesion\ diameters\ at\ tumor\ assessment\ after\ study\ treatment - sum\ of\ target\ lesion\ diameters\ at\ baseline)}{sum\ of\ target\ lesion\ diameters\ at\ baseline}$$

#### 8.4.1 Evaluation of Secondary Endpoints

1) Progressive-free survival (PFS)

PFS will be estimated using the Kaplan-Meier method, and the event occurrence rate, median PFS, and its 95% CI will be presented. The Kaplan-Meier survival plot will also be provided.

• PFS is defined as the period from the first administration of the investigational drug to the time of progression or death from any cause, with the earlier event being considered.

2) Overall survival (OS)

Overall Survival (OS) will be estimated using the Kaplan-Meier method, and the event occurrence rate, median OS, and its 95% CI will be presented. The Kaplan-Meier survival plot will also be provided.

• OS is defined as the period from the start of administration to the time of death from any cause or the cutoff point.

3) Objective Response Rate According to Modified RECIST

Present the number and percentage of subjects who showed an objective response in the Best overall response evaluated by Modified RECIST, along with the 95% exact confidence interval.

### 8.5 Safety Analysis

Safety analysis will be performed for safety analysis set.

#### 8.5.1 Adverse Reaction

The analysis of adverse reactions focuses on adverse reactions that newly occurred or worsened after the administration of the investigational medicinal product (Treatment-emergent adverse events, TEAEs).

The number of subjects who experienced adverse reactions (TEAE), drug adverse reactions (ADR), serious adverse reactions (SAE), adverse reactions leading to death, and adverse reactions causing dropout after the administration of the investigational medicinal product, the incidence rate, and the 95% confidence interval of the incidence rate are presented. Additionally, the number of subjects, incidence rate, and number of cases for adverse reactions, drug adverse reactions, serious adverse reactions, adverse reactions leading to death, and adverse reactions causing dropout after the administration of the investigational medicinal product are presented by SOP and PT. Detailed information (screening number, safety analysis group, gender, age, first administration date of the main product, adverse reaction name (SOC/PT), onset date, disappearance date, severity, SAE status, relationship with Regorafenib, relationship with Nivolumab, measures taken related to IP,

measures taken outside of IP, result) is provided for serious adverse reactions, adverse reactions leading to death, and adverse reactions causing dropout.

- Adverse reactions (TEAE) occurring after the administration of the investigational medicinal product are defined as those with an onset "after treatment start (after C1D1)". However, adverse reactions that are judged to be related after administration, regardless of their occurrence before administration, are also included.
- Drug adverse reactions (ADR) are defined as those with a relationship to Regorafenib that is "Definitely related", "Probably related", "Possibly related", "Unlikely related", "Unclassified", "Unassessable", or with a relationship to Nivolumab that is "Definitely related", "Probably related", "Possibly related", "Unlikely related", "Unclassified", "Unassessable".
- Serious adverse reactions (SAE) are defined as "Death", "Life-threatening", "Hospitalization", "Significant disability", "Congenital anomaly/birth defect", or "Important medical event".
- Adverse reactions leading to death are defined as those with a result of "Fatal".
- Adverse reactions causing early termination/dropout are defined as those with measures taken related to the investigational medicinal product recorded as "Treatment withdrawn Regorafenib", "Treatment withdrawn Nivolumab", or "Treatment withdrawn Regorafenib/Nivolumab".

#### 8.5.2 Vital sign

For vital signs, descriptive statistics (number of subjects, mean, standard deviation, median, minimum, maximum) for results and changes before and after the administration of the investigational medicinal product are presented. Depending on the satisfaction of the normality assumption, either the paired t-test or the Wilcoxon signed-rank test is conducted.

- Vital signs: Weight (kg), Systolic blood pressure (mmHg), Diastolic blood pressure (mmHg), Pulse (beats/min), Body temperature (℃), Respiratory rate (breaths/min).

#### 8.5.3 Physical Examination

For the physical examination, the frequency and ratio of normal/abnormal changes before and after the administration of the investigational medicinal product at each visit are presented. The McNemar’s test is conducted to assess the changes before and after administration.

#### 8.5.4 Clinical Laboratory Tests

For hematology, clinical chemistry tests, and coagulation tests, descriptive statistics (number of subjects, mean, standard deviation, median, minimum, maximum) for changes before and after the administration of the investigational medicinal product, as well as the amount of change, are presented. Depending on the

satisfaction of the normality assumption, either the paired t-test or the Wilcoxon signed-rank test is conducted for changes before and after administration.

For hematology tests, clinical chemistry tests, coagulation tests, and urine tests, the frequency and ratio of changes from normal/clinically non-significant abnormal (NCS) to clinically significant abnormal (CS) after administration compared to before administration are presented. If there are test results confirmed as clinically significant abnormal (CS), detailed information (screening number, age, visit, test item, test result, remarks) is presented, and the McNemar's test is conducted to assess the changes before and after administration.

For laboratory test items with different units by institution, the units are converted as follows for analysis.

| Hematology test | | | |
|---|---|---|---|
| No. | Item | Standard Unit | Comment |
| 1 | Red blood cell count | $10^6$/L | $10^6$/L=$100^3$/L |
| 2 | Mean corpuscular hemoglobin concentration | % | 1% = 1g/dL |
| Clinical chemistry test | | | |
| No. | Item | Standard Unit | Comment |
| 1 | Alkaline phosphatase | IU/L | 1IU/L = 1U/L |
| 2 | Alanine aminotransferase (ALT) | IU/L | 1IU/L = 1U/L |
| 3 | Aspartate aminotransferase (AST) | IU/L | 1IU/L = 1U/L |
| 4 | Bicarbonate | mmol/L | 1mEq/L = 1mmol/L |
| 5 | Chloride | mmol/L | 1mEq/L = 1mmol/L |

#### 8.5.5 ECOG performance status analysis

The frequency and ratio of the ECOG scores measured before the administration of the investigational medicinal product and at each visit are presented. The McNemar-Bowker test is conducted to assess the changes before and after administration.

## 9. Appendix

Dummy Table

Listing

Definition of Analysis Population

## 10. Reference

Not applicable

## 11. List of Applicable SOPs

| Document ID | Version | Document Title | Classification | Effective Date |
|---|---|---|---|---|
| GEN-GLSOP-SA04-00 | 1.0 | Statistical Analysis | Procedure | 20/May/2020 |
| GEN-GLSOP-SA04-WI01-00 | 2.0 | Statistical Analysis Plan | Procedure | 15/Dec/2020 |
| GEN-GLSOP-SA04-WI02-00 | 1.0 | Statistical Analysis Report | Procedure | 20/May/2020 |
| GEN-GLSOP-SA04-WI03-00 | 2.0 | SAS Programming | Procedure | 28/Feb/2020 |
| GEN-GLSOP-SA04-WI04-00 | 3.0 | Quality Control of Statistical Analysis | Procedure | 15/Dec/2020 |
| GEN-GLSOP-SA04-WI06-00 | 1.0 | Delivery of Deliverables | Procedure | 20/May/2020 |
| GEN-KRSOP-SA03-00 | 1.0 | Statistical Analysis process in Korea | Procedure | 16/May/2019 |
| GEN-KRSOP-SA03-01 | 1.0 | Statistical Analysis Plan Template in Korea | Attachment | 16/May/2019 |
| GEN-KRSOP-SA03-02 | 1.0 | Definition of Analysis Population | Attachment | 16/May/2019 |
| GEN-KRSOP-SA03-03 | 1.0 | Analysis Population Approval Form | Attachment | 16/May/2019 |
| GEN-KRSOP-SA03-04 | 1.0 | Analysis Dataset Specification | Attachment | 16/May/2019 |
| GEN-KRSOP-SA03-06 | 1.0 | SAS Program Completion Form | Attachment | 16/May/2019 |
| GEN-KRSOP-SA03-07 | 1.0 | SAS Program Change Log | Attachment | 16/May/2019 |
| GEN-KRSOP-SA03-08 | 1.0 | Statistical Analysis Report Template in Korea | Attachment | 16/May/2019 |
| GEN-KRSOP-SA03-09 | 1.0 | Statistical Analysis Report Check List | Attachment | 16/May/2019 |
| GEN-GLSOP-DA01-06 | 1.0 | Trial Master File Index and Checklist | Attachment | 26/Dec/2019 |
| GEN-GLSOP-DA01-07 | 1.0 | Note to File | Attachment | 26/Dec/2019 |
| GEN-GLSOP-GA10-01 | 1.0 | All Staff Transition Checklist | Attachment | 06/Sep/2019 |
| GEN-GLSOP-PM01-02 | 1.0 | Project Member Contact List | Attachment | 31/Mar/2020 |
| GCP-GLSOP-CO03-10 | 1.0 | Project Specific Training Record | Attachment | 31/Mar/2020 |

# Statistical Analysis Plan

| | |
|---|---|
| **Sponsor** | Asan Medical Center |
| **Study Title** | Phase II study of regorafenib-nivolumab combination therapy for chemotherapy-naïve patients with unresectable or metastatic hepatocellular carcinoma |
| **Protocol No.** | RENOBATE |
| **Version** | v1.0 |

| **Prepared by:** | | | | | |
|---|---|---|---|---|---|
| **Name:** | Jiyoung Jung | **Signature:** | | **Date:** | |
| **Title:** | Sr. Biostatistician | | **Department:** | SS Dept, SA team | |
| **Confirmed by CMIC Korea:** | | | | | |
| **Name:** | Keumna Hwang | **Signature:** | | **Date:** | |
| **Title:** | Sr. SA Manager | | **Department:** | SS Dept, SA team | |
| **Approved by Sponsor:** | | | | | |
| **Name:** | Changhoon Yoo | **Signature:** | | **Date:** | |
| **Title:** | Assistant Professor | | **Department:** | Department of Oncology, Asan Medical Center | |

# Version Information

| Version/Notation | Prepared | Details |
|---|---|---|
| 1.0 | Jiyoung Jung | Newly created |

# Abbreviation

| Term | Definition |
| --- | --- |
| AASLD | American Association for the Study of Liver Diseases |
| AE | Adverse Event |
| aPTT | activated Partial Thromboplastin Time |
| BCLC | Barcelona Clinic Liver Cancer |
| CNS | Central nervous system |
| COPD | Chronic obstructive pulmonary disease |
| CR | Complete remission |
| CxDx | Cycle x Day x |
| ECOG | Eastern Cooperative Oncology Group |
| FACS | Fluorescence activated cell sorting |
| HBV | Hepatitis B virus |
| HCC | Hepatocellular carcinoma |
| HCV | Hepatitis C virus |
| HIV | Human immunodeficiency virus |
| INR | International normalized ratio |
| MedDRA | Medical Dictionary for Regulatory Activities |
| ORR | Objective response rate |
| OS | Overall survival |
| PD | Progressive disease |
| PD-1 | Programmed cell death 1 |
| PFS | Progressive free survival |
| PT | Preferred Term |
| PR | Partial remission |
| RECIST | Response evaluation criteria in solid tumors |
| SAE | Serious adverse event |
| SOC | System Organ Class |

# Table of Contents


1. **Background** .......... 6
2. **Duties, Responsibilities and Roles** .......... 6
   - 2.1 **Duties** .......... 6
   - 2.2 **Responsibilities and Roles** .......... 6
3. **Study Design** .......... 6
   - 3.1 **Study Design** .......... 6
   - 3.2 **Objectives** .......... 6
     - 3.2.1 **Primary Objective** .......... 6
     - 3.2.2 **Secondary Objectives** .......... 6
     - 3.2.3 **Exploratory Objectives** .......... 7
   - 3.3 **Target Disease** .......... 7
   - 3.4 **Inclusion and Exclusion Criteria** .......... 7
     - 3.4.1 **Inclusion Criteria** .......... 7
     - 3.4.2 **Exclusion Criteria** .......... 8
   - 3.5 **Investigational Products** .......... 11
4. **Clinical Trial Procedure and Process** .......... 12
   - 4.1 **Trial Procedure** .......... 12
5. **Target Patient Number** .......... 12
   - 5.1 **Determination of Sample Size** .......... 12
   - 5.2 **Rationale for Sample Size Calculation** .......... 13
6. **Analysis Set** .......... 13
   - 6.1 **Efficacy Analysis Set** .......... 13
   - 6.2 **Safety Analysis Set** .......... 13
   - 6.3 **Exploratory Biomarker Analysis Set** .......... 13
7. **Study Endpoints** .......... 13
   - 7.1 **Primary Efficacy Endpoint** .......... 13
   - 7.2 **Secondary Efficacy Endpoints** .......... 13
   - 7.3 **Safety Profile Variables** .......... 13
   - 7.4 **Exploratory Study Endpoints** .......... 14
8. **Statistical Analysis Method** .......... 14
   - 8.1 **General Principles of Statistical Analysis** .......... 14
     - 8.1.1 General Considerations for Statistical Analysis .......... 14
     - 8.1.2 Handling of Missing Data and Missing Values .......... 14
     - 8.1.3 Adjustment for Covariates .......... 14
     - 8.1.4 Multiple Comparisons or Multiplicity .......... 14
     - 8.1.5 Format of Data Presentation .......... 14
     - 8.1.6 Format for Evaluation Time Points .......... 14

8.1.7 Software ... 15
8.1.8 Dictionaries ... 15
**8.2 Evaluation of Clinical Trial Participation ... 15**
8.2.1 Clinical Trial Participation Status ... 16
8.2.2 Clinical Trial Participation Status of Subjects by Participating Institution ... 16
8.2.3 Dropout ... 16
8.2.4 Detailed Information on Subjects Excluded from the Analysis Group ... 16
**8.3 Demographic Information and Baseline Characteristics ... 16**
8.3.1 Demographic Information and Baseline Characteristics ... 16
8.3.2 History of Liver Cancer ... 17
8.3.3 History of liver cancer treatment ... 17
8.3.4 Past Medical History & Current Medical History ... 18
8.3.5 Prior/Concomitant Medication ... 18
8.3.6 Administration Status of the Investigational Drug ... 18
**8.4 Efficacy Evaluation ... 19**
8.4.1 Evaluation of Primary Endpoint ... 19
8.4.1 Evaluation of Secondary Endpoints ... 20
**8.5 Safety Analysis ... 20**
8.5.1 Adverse Reaction ... 20
8.5.2 Vital sign ... 21
8.5.3 Physical Examination ... 21
8.5.4 Clinical Laboratory Tests ... 21
8.5.5 ECOG performance status analysis ... 22
**9. Appendix ... 22**
**10. Reference ... 23**
**11. List of Applicable SOPs ... 23**

# 1. Background

This SAP (Statistical Analysis Plan) details the statistical analysis plan for the clinical trial protocol RENOBATE, which is a 'Phase II study of regorafenib-nivolumab combination therapy for chemotherapy-naïve patients with unresectable or metastatic hepatocellular carcinoma'.

# 2. Duties, Responsibilities and Roles

## 2.1 Duties

| No | Scope | CMIC | Sponsor | Assigned person | Comments |
|---|---|---|---|---|---|
| 1 | Statistical Analysis Plan | ☒ | ☐ | Biostatistician | |
| 2 | Statistical Analysis | ☒ | ☐ | Biostatistician | |
| 3 | Statistical Analysis Result | ☒ | ☐ | Biostatistician | |

## 2.2 Responsibilities and Roles

| Role | Responsibility | Comments |
|---|---|---|
| Project Biostatistician | Statistical Analysis | |
| SA Team Leader | QC of Statistical Analysis | |
| Sponsor | Approval of Statistical Analysis Plan and Statistical Analysis Result | |

# 3. Study Design

## 3.1 Study Design

This study is a phase II, open-label, non-blinding, non-comparative, multicenter trial for patients with advanced HCC.

## 3.2 Objectives

### 3.2.1 Primary Objective

To evaluate the objective response rates (defined by RECIST v1.1) of regorafenib-nivolumab combination in unresectable HCC patients

### 3.2.2 Secondary Objectives

To evaluate PFS, OS, safety profile and modified RECIST with regorafenib-nivolumab combination in patients with unresectable HCC

### 3.2.3 Exploratory Objectives

To identify biomarkers of regorafenib-nivolumab combination for clinical outcomes in patients with HCC using Circulating tumor DNA, single cell RNA sequencing and Fluorescence activated cell sorting (FACS) analysis.

## 3.3 Target Disease

Unresectable or metastatic HCC

## 3.4 Inclusion and Exclusion Criteria

### 3.4.1 Inclusion Criteria

Having provided written consent before participation in the study, patients must fulfill all of the following criteria:

1. Age ≥ 19 years at time of signing Informed Consent Form
2. Ability to comply with the study protocol, in the investigator's judgment
3. HCC that was histologically/cytologically confirmed or clinically diagnosed by AASLD criteria in cirrhotic patients. Patients without liver cirrhosis require histological confirmation of HCC
4. Locally advanced unresectable or metastatic disease that is not amenable to curative surgical a nd/or locoregional therapies, or that progressed after surgical and/or locoregional therapies
5. No prior systemic therapy for HCC
6. At least one measurable (per RECIST 1.1) lesion as confirmed by imaging within 28 days prior to initiation of study treatment
7. Patients who received prior local therapy (e.g., radiofrequency ablation, percutaneous ethanol or acetic acid injection, cryoablation, high-intensity focused ultrasound, transarterial chemoemb olization, transarterial embolization, etc.) are eligible provided that other target lesion(s) have n ot been previously treated with local therapy or the target lesion(s) within the field of local thera py have subsequently progressed in accordance with RECIST 1.1.
8. Pre-treatment tumor tissue sample (if available)
   - If tumor tissue is available, approximately 10–30 slides containing unstained, freshly cut, serial sections will be required subsequently for translational research.
   - If tumor tissue is not available (e.g., depleted because of prior diagnostic testing), patients are still eligible.
9. ECOG Performance Status score 0 or 1
10. Child-Pugh class A
11. Adequate hematologic and end-organ function, defined by the following laboratory test results, obtained within 14 days prior to initiation of study treatment, unless otherwise specified:
    - ANC ≥ 1.0 × $10^9$/L (1000/μL) without granulocyte colony-stimulating factor support
    - Platelet count ≥ 75 × $10^9$/L (75,000/μL) without transfusion

- Hemoglobin ≥ 90 g/L (9 g/dL): Patients may be transfused to meet this criterion.
- AST, ALT, and alkaline phosphatase (ALP) ≤ 3 × upper limit of normal (ULN)
- Serum bilirubin ≤ 2× ULN
- Serum creatinine ≤ 1.5 × ULN or creatinine clearance ≥ 50 mL/min (calculated using the Cockcroft-Gault formula)
- Serum albumin ≥ 28 g/L (2.8 g/dL)
- For patients not receiving therapeutic anticoagulation: INR or aPTT ≤ 2× ULN
- Urine dipstick for proteinuria < 2+
- Patients discovered to have ≥ 2+ proteinuria on dipstick urinalysis at baseline should undergo a 24-hour urine collection and must demonstrate < 1 g of protein in 24 hours.

12. Resolution of any acute, clinically significant treatment-related toxicity from prior therapy to Gra de ≤ 1 prior to study entry, with the exception of alopecia
13. Negative HIV result at screening test or prior tested conducted within 3 years
14. Documented virology status of hepatitis, as confirmed by screening HBV and HCV serology tes t
    - Patients with active hepatitis B virus (HBV) must meet the followings: HBV DNA < 500 IU/mL obtained within 14 days prior to initiation of study treatment, anti-HBV treatment (per local standard of care; e.g., entecavir) for a minimum of 14 days prior to study entry and willingness to continue treatment for the length of the study
15. Women of childbearing potential (including women with chemical menopause or no menstruatio n for other medical reasons)#1 must agree to use contraception#2 from the time of informed con sent until 5 months or more after the last dose of the investigational product. Also, women must agree not to breastfeed from the time of informed consent until 5 months or more after the last dose of the investigational product.
16. Men must agree to use contraception#2 from the start of study treatment until 7 months or more after the last done of the investigational product.

#1 Women of childbearing potential are defined as all women after the onset of menstruation who are not postmenopausal and have not been surgically sterilized (e.g., hysterectomy, bilateral tubal ligation, bilateral oophorectomy). Postmenopause is defined as amenorrhea for ≥12 consecutive months without specific reasons. Women using oral contraceptives, intrauterine devices, or mechanical contraception such as contraceptive barriers are regarded as having childbearing potential.

#2 The subject must consent to use any two of the following methods of contraception: vasectomy or condom for patients who are male or female subject's partner and tubal ligation, contraceptive diaphragm, intrauterine device, spermicide, or oral contraceptive for patients who are female or male subject's partner.

### 3.4.2 Exclusion Criteria

Patients who meet any of the following criteria at the time of screening will be excluded. If a subject is found to meet any of the following criteria before the first dose of the investigational product, the subject will not be started on the study treatment and will be withdrawn from the study.

1. Patients who are diagnosed with fibrolamellar HCC, sarcomatoid HCC, or combined type of ch olangiocarcinoma and HCC
2. Patients with a history of malignancy other than HCC within 3 years prior to screening, with the exception of malignancies with a negligible risk of metastasis or death (e.g., 5-year OS rate > 9 0%), such as adequately treated carcinoma in situ of the cervix, non-melanoma skin carcinoma, localized prostate cancer, ductal carcinoma in situ, and Stage I uterine cancer
3. Patients with a history of leptomeningeal seeding
4. Patients with symptomatic, untreated, or actively progressing central nervous system (CNS) me tastases.
   - Asymptomatic patients with treated CNS lesions are eligible, provided that all of the following criteria are met:
     (1) The patients must have at least one measurable lesion, per RECIST 1.1, other than CNS metastases
     (2) The patient must not have a history of intracranial hemorrhage or spinal cord hemorrhage
     (3) The metastatic lesions have to be limited in cerebellum or supratentorial region (e.g., not to the midbrain, pons, medulla, or spinal cord)
     (4) There must be no evidence of interim progression between the completion of CNS-directed therapy and initiation of the study treatment
     (5) The patient must not undergo stereotactic radiotherapy within 7 days, whole-brain radiotherapy within 14 days, or neurosurgical resection within 28 days prior to initiation of the study treatment
     (6) The patient must not have ongoing requirement for corticosteroids for CNS disease
   - Anticonvulsant therapy at a stable dose is permitted.
   - Asymptomatic patients with CNS metastases newly detected at screening are eligible for the study after receiving radiotherapy or surgery, with no need to repeat the screening brain scan.
5. Patients with current of past history of autoimmune disease or immunodeficient disease (includi ng, but not limited to, myasthenia gravis, myositis, autoimmune hepatitis, systemic lupus erythe matosus, rheumatoid arthritis, inflammatory bowel disease, antiphospholipid antibody syndrom e, Wegener granulomatosis, Sjögren syndrome, Guillain-Barré syndrome, or multiple sclerosis) with the following exceptions:
   - Patients with autoimmune-related hypothyroidism who are on thyroid-replacement hormone are eligible.
   - Patients with controlled Type 1 diabetes mellitus who are on an insulin regimen are eligible.
   - Patients with eczema, psoriasis, lichen simplex chronicus, or vitiligo with dermatologic manifestations only (e.g., patients with psoriatic arthritis are excluded) are eligible for the study provided all of following conditions are met:
     (1) Rash must cover < 10% of body surface area
     (2) Disease has to be well controlled at baseline and requires only low-potency topical corticosteroids
     (3) There must be no occurrence of acute exacerbations of the underlying condition requiring psoralen plus ultraviolet A radiation, methotrexate, retinoids, biologic agents,

oral calcineurin inhibitors, or high-potency or oral corticosteroids within the previous 12 months

6. Patients with current or past history of idiopathic pulmonary fibrosis, organizing pneumonia (e. g., bronchiolitis obliterans), drug-induced pneumonitis, or idiopathic pneumonitis, or evidence of active pneumonitis on screening chest computed tomography (CT) scan.
   - Patients with history of radiation pneumonitis in the radiation field (fibrosis) are eligible if the radiation pneumonitis has been confirmed as stable (beyond acute phase) without any concerns about recurrence.
7. Patients who have experienced a transient ischemic attack, cerebrovascular accident, thrombo sis, or thromboembolism (pulmonary arterial embolism or deep vein thrombosis) within 6 month s before initiation of study treatment
8. Patients with a history of uncontrollable or significant cardiovascular disease meeting any of the following criteria:
   - Myocardial infarction within 6 months before initiation of study treatment
   - Uncontrollable angina pectoris within 6 months before initiation of study treatment
   - New York Heart Association Class II or greater congestive heart failure within 6 months before initiation of study treatment
   - Uncontrollable hypertension despite appropriate treatment (e.g., systolic blood pressure ≥150 mmHg or diastolic blood pressure > 90 mmHg based on an average of ≥ 3 BP readings on ≥ 2 sessions)
   - Arrhythmia requiring treatment
9. Patients with congenital long QT syndrome or corrected QT interval > 450 ms (calculated with u se of the Fridericia method) at screening
10. Patients with systemic infections (including active tuberculosis) requiring treatment
11. Patients with history of hypertensive crisis or hypertensive encephalopathy
12. Patients with significant vascular disease (e.g., aortic aneurysm requiring surgical repair or rece nt peripheral arterial thrombosis) within 6 months prior to initiation of study treatment
13. Patients who underwent major surgical procedure, other than for diagnosis, within 4 weeks prio r to initiation of study treatment or who are expected to need a major surgical procedure during the study
14. Patients who have received radiotherapy within 28 days before initiation, or radiotherapy to bon e metastases within 14 days before initiation
15. Patients with prior history of allogeneic stem cell or solid organ transplantation
16. Patients with current or past history of severe allergic anaphylactic reactions to chimeric or hum anized antibodies or fusion proteins
17. Patients with untreated or incompletely treated varices with active bleeding or high risk for blee ding
18. Patients with moderate or severe ascites
19. Patients with history of hepatic encephalopathy
20. Patients with evidence of bleeding diathesis or significant coagulopathy (in the absence of ther apeutic anticoagulation)
21. Patients who had recent (within 10 days of first dose of study treatment) use of aspirin (> 300 m g/day) or treatment with dipyramidole, ticlopidine, clopidogrel, and cilostazol
22. Patients who had recent use of full-dose oral or parenteral anticoagulants or thrombolytic agent s for therapeutic (as opposed to prophylactic) purpose

- Prophylactic anticoagulation for the patency of venous access devices is allowed provided the activity of the agent results in an INR < 1.5 × ULN and aPTT within normal limits within 14 days prior to initiation of study treatment.
- Prophylactic use of low molecular-weight heparin (i.e., enoxaparin 40 mg/day) is allowed.

23. Patients who treated with strong CYP3A4 inducers within 14 days prior to initiation of study trea tment, including rifampin (and its analogues) or St. John's wort
24. Patients who have previously received CD137 agonists or immune checkpoint blockade therapi es, including anti−CTLA-4, anti−PD-1, and anti−PD-L1 therapeutic antibodies
25. Patients who were treated with systemic immunostimulatory agents (including, but not limited t o, interferon and interleukin 2 [IL-2]) within 4 weeks or 5 half-lives of the drug (whichever is long er) prior to initiation of study treatment
26. Patients who were treated with systemic immunosuppressive medication (including, but not limi ted to, corticosteroids, cyclophosphamide, azathioprine, methotrexate, thalidomide, and anti−T NF-α agents) within 2 weeks prior to initiation of study treatment, or anticipation of need for sys temic immunosuppressive medication during study treatment, with the following exceptions:
    - Patients who received temporary, low-dose systemic immunosuppressant medication or a one-time pulse dose of systemic immunosuppressant medication (e.g., 48 hours of corticosteroids for a contrast allergy) are eligible for the study.
    - Patients who received mineralocorticoids (e.g., fludrocortisone), or corticosteroids for chronic obstructive pulmonary disease (COPD) or asthma, or low-dose corticosteroids for orthostatic hypotension or adrenal insufficiency are eligible for the study.
27. Patients who had abdominal or tracheoesophageal fistula, gastrointestinal (GI) perforation, or i ntra-abdominal abscess within 6 months prior to initiation of study treatment
28. Patients who had intestinal obstruction and/or clinical signs or symptoms of GI obstruction inclu ding sub-occlusive disease related to the underlying disease or requirement for routine parente ral hydration, parenteral nutrition, or tube feeding within 6 months prior to initiation of study trea tment
    - Patients with signs/symptoms of sub-/occlusive syndrome/intestinal obstruction at time of initial diagnosis may be enrolled if they had received definitive (surgical) treatment for symptom resolution.
29. Women who are pregnant or breastfeeding, or possibly pregnant
30. Other patients judged by the investigator or sub-investigator to be inappropriate as subjects of t his study

### 3.5 Investigational Products

1) Nivolumab

| | |
|---|---|
| Product name/dose | Nivolumab (OPDIVO®), 100mg/10mL (10mg/mL)/vial |
| Formulation and packaging | Clear to opalescent, colorless to pale-yellow solution |
| Storage | A secure, limited-access location under the storage conditions specified on the label (2 ~ 8℃) |
| Administration | Intravenous |

2) Regorafenib

| | |
|---|---|
| Product name/dose | Regorafenib (Stivarga®), 40mg/tab |
| Formulation and packaging | light pink, oval shaped, film-coated tablet |
| Storage | Drug bottle between 15 ~ 30℃ |
| Administration | Oral |

# 4. Clinical Trial Procedure and Process

## 4.1 Trial Procedure

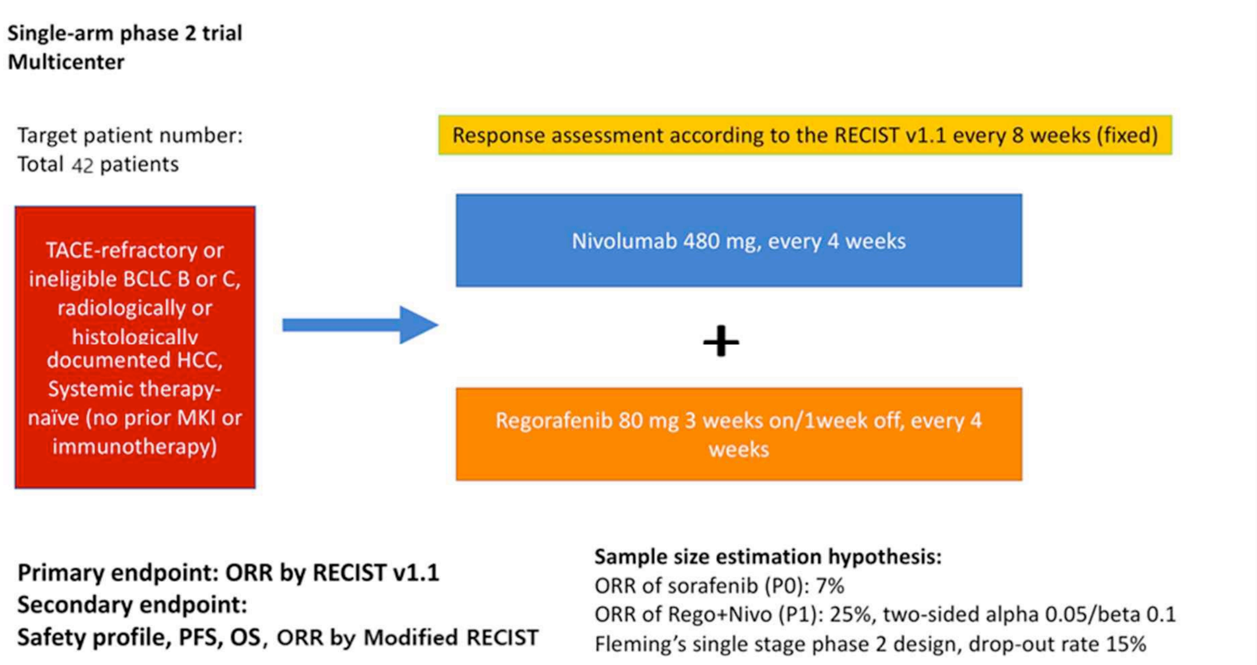


All eligible patients will be treated with nivolumab 480 mg intravenous at day 1 and regorafenib 80 mg at days 1-21, every 4 weeks.

Tumor response will be performed every 8 weeks and graded per RECIST 1.1

# 5. Target Patient Number

## 5.1 Determination of Sample Size

With alpha of two sided 0.05 and power of 90%, 35 patients are needed based on Fleming's single-stage Phase 2 design calculation. Considering 15% of follow-up loss rates, a total 42 patients are needed for this study.

### 5.2 Rationale for Sample Size Calculation

In the previous phase III trials, sorafenib, current standard 1st line therapy, showed the objective response rates (ORR) of 7% (P0) graded by RECIST v1.1. With the regorafenib-Nivolumab combination, we assumed that the partial response rates might be enhanced to 25% (P1).

## 6. Analysis Set

### 6.1 Efficacy Analysis Set

Efficacy analysis will be performed on the intent-to-treat (ITT) population. All participants will be candidates for efficacy analysis.

### 6.2 Safety Analysis Set

All participants, who were administered any dose of any study intervention will be candidates for safety analysis. Analyses will consider these participants as treated.

### 6.3 Exploratory Biomarker Analysis Set

Patient subgroup for the correlative biomarker analyses using circulating DNA, single cell RNA sequencing and FACS analysis will be defined after the primary analysis for the clinical outcomes considering the unexpected nature of clinical outcomes for novel therapeutic regimen.

## 7. Study Endpoints

### 7.1 Primary Efficacy Endpoint

- Objective response rate (ORR) per RECIST 1.1 (Proportion of complete response [CR] and partial response [PR])

### 7.2 Secondary Efficacy Endpoints

- Progressive free survival (PFS)
- Overall survival (OS)
- ORR per modified RECIST

### 7.3 Safety Profile Variables

- Adverse events
- Vital sign
- Physical examination
- Laboratory evaluation
- ECOG performance status

### 7.4 Exploratory Study Endpoints

- Circulating tumor DNA
- Single cell RNA sequencing
- Fluorescence activated cell sorting (FACS) analysis

## 8. Statistical Analysis Method

### 8.1 General Principles of Statistical Analysis

#### 8.1.1 General Considerations for Statistical Analysis

For continuous data in this clinical trial, descriptive statistics such as the number of test subjects, mean, standard deviation, median, minimum, and maximum values are provided. For categorical data, frequencies and percentages (%) are presented. All statistical analyses are performed with a two-sided test at a significance level of 5%.

#### 8.1.2 Handling of Missing Data and Missing Values

Missing values are not replaced with other values.

#### 8.1.3 Adjustment for Covariates

Adjustment for covariates is not considered in this clinical trial.

#### 8.1.4 Multiple Comparisons or Multiplicity

Multiple comparisons or multiplicity are not considered in this clinical trial

#### 8.1.5 Format of Data Presentation

Mean, standard deviation, median, minimum, maximum values, ratios, and confidence intervals are rounded to two decimal places.

#### 8.1.6 Format for Evaluation Time Points

Efficacy or safety evaluation variables are evaluated considering a 4-week treatment period as one cycle based on Screening and C1D1. The summary of each evaluation variable by time point will be presented according to regular visits as follows.

| Visit | Analysis Label | Actual Point | Visit window |
|---|---|---|---|
| Screening | Baseline | -Week 8 ~ Week 0 | - week 4 ~ - day 1 |
| C1D1 | C1D1 | Week 0 | |
| C1D15 | C1D15 | C1D1 + week 2 | ± day 3 |
| C2D1 | C2D1 | C1D1 + week 4 | ± day 3 |
| C2D15 | C2D15 | C1D1 + week 6 | ± day 3 |
| C3D1 | C3D1 | C1D1 + week 8 | ± day 3 |
| C3D15 | C3D15 | C1D1 + week 10 | ± day 3 |
| C4D1 | C4D1 | C1D1 + week 12 | ± day 3 |
| CkD1 | CkD1 | C1D1 + (k-1) X week 4 | ± day 3 |
| End of Treatment | EOT | Assessment taken at the end of treatment visit | |

The time point before drug administration (Baseline) in statistical analysis is defined as follows.

1) Safety Evaluation Items
    - Vital Signs: C1D1 (Before IP administration)
    - Physical Examination: C1D1 (Before IP administration)
    - Clinical Laboratory Test: C1D1 (Before IP administration)
    - ECOG Performance Status: C1D1 (Before IP administration)
    - Electrocardiogram Test: Screening

#### 8.1.7 Software

All statistical analysis will be conducted using SAS (Statistical Analysis System) over 9.4 version..

#### 8.1.8 Dictionaries

1) Past and current medical history will be standardized in English using MedDRA (the latest version) according to the System Organ Class (SOC) and Preferred Term (PT).
2) Adverse reactions will be standardized in English using MedDRA (the latest version) according to the System Organ Class (SOC) and Preferred Term (PT).
3) Concomitant and prior medications will be standardized in English using the ATC Index Code (the latest version) based on the anatomical category (level 1) and therapeutic category (level 2).
4) When preparing the Statistical Analysis Report (SAR), the dictionary version used must be clearly specified.

### 8.2 Evaluation of Clinical Trial Participation

### 8.2.1 Clinical Trial Participation Status

The clinical trial participation status is summarized as follows and presented in tables and diagrams. In the case of diagrams, only the number of subjects is presented. The ratio is calculated based on the number of subjects registered for the clinical trial.

- Number of subjects for screening, screening dropout, and reasons for screening dropout
- Number of subjects registered for the clinical trial
- Number and ratio of subjects who completed the clinical trial, dropped out midway, and reasons for dropping out midway
- Number and ratio of subjects in the safety analysis group, excluded from the safety analysis group, and reasons for exclusion
- Number and ratio of subjects in the efficacy evaluation group, excluded from the efficacy evaluation group, and reasons for exclusion.

### 8.2.2 Clinical Trial Participation Status of Subjects by Participating Institution

The number and ratio of subjects' clinical trial participation status are presented according to the test institution.

- Clinical trial participating institution, screening, clinical trial registration, clinical trial completion, safety analysis group, efficacy evaluation group

### 8.2.3 Dropout

The detailed reasons for the dropout of the subjects who dropped out midway are presented.

- Screening number, safety analysis group, efficacy evaluation group, gender, age, reason for dropout

### 8.2.4 Detailed Information on Subjects Excluded from the Analysis Group

The detailed reasons for the exclusion of subjects from each analysis group are presented by the administered group.

- Analysis group, screening number, gender, age, reason for exclusion

## 8.3 Demographic Information and Baseline Characteristics

Demographic information and characteristics prior to administration are presented for the efficacy evaluation group.

### 8.3.1 Demographic Information and Baseline Characteristics

For continuous variables related to demographic information and baseline characteristics, descriptive statistics (number of subjects, mean, standard deviation, median, minimum, maximum) are presented. For categorical variables, frequency and ratio are presented.

• Continuous data: Age (years), height (cm), weight (kg), BMI (kg/m^2)

• Categorical data: Gender, age group (19-29 years, 30-39 years, 40-49 years, 50-59 years, 60-69 years, 70 years and above)

• Derived data:

BMI ($kg/m^2$) = weight (kg) / (height (cm)/100)$^2$

### 8.3.2 History of Liver Cancer

For continuous variables related to the history of liver cancer, descriptive statistics (number of subjects, mean, standard deviation, median, minimum, maximum) are presented. For categorical variables, frequency and ratio are presented.

- Continuous data: Duration of illness (days)
- Categorical data: Diagnostic method, disease status at the time of screening, presence of liver cirrhosis, BCLC stage, presence of hepatitis B, presence of hepatitis C, presence of fatty liver, presence and location of extrahepatic spread
- Derived data:
  - Duration of illness (days) = Date of consent signing - Date of diagnosis
  - Presence of Hepatitis B: If 'Hepatitis B' is collected as 'Y', then 'Yes', otherwise 'No'
  - Presence of Hepatitis C: If 'Hepatitis C' is collected as 'Y', then 'Yes', otherwise 'No'
  - Presence of Fatty liver: If 'Non-alcoholic (NASH)' is collected as 'Y' or 'Alcoholic' is collected as 'Y', then 'Yes', otherwise 'No'
  - Presence of extrahepatic spread - Others: If one or more of the 'Extrahepatic spread-Others' or 'Metastatic site_Others' items are 'Yes', then 'Yes', if all are 'No', then 'No'
  - Presence of extrahepatic spread: If one or more of the detailed extrahepatic spread items (central nervous system, lungs, bones, peritoneal seeding, lymph nodes, others) are 'Yes', then 'Yes', if all are 'No', then 'No'

### 8.3.3 History of liver cancer treatment

Regarding the history of liver cancer treatment, present the frequency and percentage (%) for whether liver cancer treatment was received, whether liver cancer surgery was performed, whether radiation therapy was received, and whether local treatment was received.

• Data derived:

Liver cancer treatment status: If there is a history of any of the treatments such as liver cancer surgery/radiation therapy/local treatment, the status of liver cancer treatment is 'Yes'. If none are present, it's 'No'.

### 8.3.4 Past Medical History & Current Medical History

Medical history is analyzed by dividing it into past medical history and current medical history. Present the frequency and percentage for the presence or absence of past and current medical history. All past and current medical histories are presented based on the System Organ Class (SOC) and Preferred Term (PT), showing the number of test subjects, percentage, and count.

The distinction between past medical history and current medical history is made based on the end date of the medical history and its ongoing status at the time of the visit.

- Past Medical History: If the end date is 'before the screening visit'.
- Current Medical History: If the end date is 'after the screening visit' or if the ongoing status of the medical history is marked as 'ongoing'.

### 8.3.5 Prior/Concomitant Medication

Medication administration history is analyzed by dividing it into prior medication and concomitant medication. Present the frequency and percentage for the administration history of prior and concomitant medications. Both prior and concomitant medications are presented based on the anatomical category (level 1) and therapeutic category (level 2), showing the number of test subjects, percentage, and count.

The distinction between prior and concomitant medications is made based on the end date of administration and its ongoing status. If it's difficult to distinguish between prior and concomitant medications, they are included as concomitant medications.

- Prior Medication: If the end date of administration is 'before the start date of the investigational drug administration'.
- Concomitant Medication: If the end date of administration is 'after the start date of the investigational drug administration' or if the ongoing status of administration is marked as 'ongoing'.

### 8.3.6 Administration Status of the Investigational Drug

Present descriptive statistics (number of test subjects, average, standard deviation, median, minimum, maximum) for the total cycles and the total dosage of both Regorafenib and Nivolumab. Also, provide the frequency and percentage for the status of cycle delays, as well as dose reduction and dose adjustment for both Regorafenib and Nivolumab.

• Total Cycles: The cycle at the last administration point.

• Total Dosage (mg) = Σ(dosage)

• Cycle Delayed: If the status of cycle delay is marked as 'Yes' at least once, it's considered 'Yes'. If all are marked 'No', it's considered 'No'.

• Dose Reduction:

- Regorafenib: If the dose status is marked as 'Reduced' or 'Discontinuation' at least once, it's considered 'Yes'. If all are marked 'No Adjustment', it's considered 'No'.

- Nivolumab: If the dose status is marked as 'Discontinuation' at least once, it's considered 'Yes'. If all are marked 'No Adjustment', it's considered 'No'.

• Dose Adjustment:

- Regorafenib: If the status of cycle delay is marked as 'Yes' at least once, or if the dose status is marked as 'Reduced' or 'Discontinuation' at least once, it's considered 'Yes'. Otherwise, it's considered 'No'.

- Nivolumab: If the status of cycle delay is marked as 'Yes' at least once, or if the dose status is marked as 'Discontinuation' at least once, it's considered 'Yes'. Otherwise, it's considered 'No'.

## 8.4 Efficacy Evaluation

The efficacy evaluation is presented for the ITT population.

### 8.4.1 Evaluation of Primary Endpoint

Provide the number and percentage of subjects who showed an objective response in the Best Overall response evaluated by RECIST 1.1, along with the 95% exact confidence interval.

• Objective Response Rates: Proportion of Complete Response (CR) or Partial Response (PR).

Additionally, the percentage change (% change) in the sum of the diameters of the target lesions was calculated and presented in the Waterfall plot and Spider plot for each subject.

- Percentage change rate (% change) =

$$\frac{(sum\ of\ target\ lesion\ diameters\ at\ tumor\ assessment\ after\ study\ treatment - sum\ of\ target\ lesion\ diameters\ at\ baseline)}{sum\ of\ target\ lesion\ diameters\ at\ baseline}$$

#### 8.4.1 Evaluation of Secondary Endpoints

1) Progressive-free survival (PFS)

PFS will be estimated using the Kaplan-Meier method, and the event occurrence rate, median PFS, and its 95% CI will be presented. The Kaplan-Meier survival plot will also be provided.

• PFS is defined as the period from the first administration of the investigational drug to the time of progression or death from any cause, with the earlier event being considered.

2) Overall survival (OS)

Overall Survival (OS) will be estimated using the Kaplan-Meier method, and the event occurrence rate, median OS, and its 95% CI will be presented. The Kaplan-Meier survival plot will also be provided.

• OS is defined as the period from the start of administration to the time of death from any cause or the cutoff point.

3) Objective Response Rate According to Modified RECIST

Present the number and percentage of subjects who showed an objective response in the Best overall response evaluated by Modified RECIST, along with the 95% exact confidence interval.

### 8.5 Safety Analysis

Safety analysis will be performed for safety analysis set.

#### 8.5.1 Adverse Reaction

The analysis of adverse reactions focuses on adverse reactions that newly occurred or worsened after the administration of the investigational medicinal product (Treatment-emergent adverse events, TEAEs).

The number of subjects who experienced adverse reactions (TEAE), drug adverse reactions (ADR), serious adverse reactions (SAE), adverse reactions leading to death, and adverse reactions causing dropout after the administration of the investigational medicinal product, the incidence rate, and the 95% confidence interval of the incidence rate are presented. Additionally, the number of subjects, incidence rate, and number of cases for adverse reactions, drug adverse reactions, serious adverse reactions, adverse reactions leading to death, and adverse reactions causing dropout after the administration of the investigational medicinal product are presented by SOP and PT. Detailed information (screening number, safety analysis group, gender, age, first administration date of the main product, adverse reaction name (SOC/PT), onset date, disappearance date, severity, SAE status, relationship with Regorafenib, relationship with Nivolumab, measures taken related to IP,

measures taken outside of IP, result) is provided for serious adverse reactions, adverse reactions leading to death, and adverse reactions causing dropout.

- Adverse reactions (TEAE) occurring after the administration of the investigational medicinal product are defined as those with an onset "after treatment start (after C1D1)". However, adverse reactions that are judged to be related after administration, regardless of their occurrence before administration, are also included.
- Drug adverse reactions (ADR) are defined as those with a relationship to Regorafenib that is "Definitely related", "Probably related", "Possibly related", "Unlikely related", "Unclassified", "Unassessable", or with a relationship to Nivolumab that is "Definitely related", "Probably related", "Possibly related", "Unlikely related", "Unclassified", "Unassessable".
- Serious adverse reactions (SAE) are defined as "Death", "Life-threatening", "Hospitalization", "Significant disability", "Congenital anomaly/birth defect", or "Important medical event".
- Adverse reactions leading to death are defined as those with a result of "Fatal".
- Adverse reactions causing early termination/dropout are defined as those with measures taken related to the investigational medicinal product recorded as "Treatment withdrawn Regorafenib", "Treatment withdrawn Nivolumab", or "Treatment withdrawn Regorafenib/Nivolumab".

### 8.5.2 Vital sign

For vital signs, descriptive statistics (number of subjects, mean, standard deviation, median, minimum, maximum) for results and changes before and after the administration of the investigational medicinal product are presented. Depending on the satisfaction of the normality assumption, either the paired t-test or the Wilcoxon signed-rank test is conducted.

- Vital signs: Weight (kg), Systolic blood pressure (mmHg), Diastolic blood pressure (mmHg), Pulse (beats/min), Body temperature (℃), Respiratory rate (breaths/min).

### 8.5.3 Physical Examination

For the physical examination, the frequency and ratio of normal/abnormal changes before and after the administration of the investigational medicinal product at each visit are presented. The McNemar’s test is conducted to assess the changes before and after administration.

### 8.5.4 Clinical Laboratory Tests

For hematology, clinical chemistry tests, and coagulation tests, descriptive statistics (number of subjects, mean, standard deviation, median, minimum, maximum) for changes before and after the administration of the investigational medicinal product, as well as the amount of change, are presented. Depending on the

satisfaction of the normality assumption, either the paired t-test or the Wilcoxon signed-rank test is conducted for changes before and after administration.

For hematology tests, clinical chemistry tests, coagulation tests, and urine tests, the frequency and ratio of changes from normal/clinically non-significant abnormal (NCS) to clinically significant abnormal (CS) after administration compared to before administration are presented. If there are test results confirmed as clinically significant abnormal (CS), detailed information (screening number, age, visit, test item, test result, remarks) is presented, and the McNemar's test is conducted to assess the changes before and after administration.

For laboratory test items with different units by institution, the units are converted as follows for analysis.

| Hematology test | | | |
|---|---|---|---|
| No. | Item | Standard Unit | Comment |
| 1 | Red blood cell count | $10^6$/L | $10^6$/L=$100^3$/L |
| 2 | Mean corpuscular hemoglobin concentration | % | 1% = 1g/dL |
| Clinical chemistry test | | | |
| No. | Item | Standard Unit | Comment |
| 1 | Alkaline phosphatase | IU/L | 1IU/L = 1U/L |
| 2 | Alanine aminotransferase (ALT) | IU/L | 1IU/L = 1U/L |
| 3 | Aspartate aminotransferase (AST) | IU/L | 1IU/L = 1U/L |
| 4 | Bicarbonate | mmol/L | 1mEq/L = 1mmol/L |
| 5 | Chloride | mmol/L | 1mEq/L = 1mmol/L |

#### 8.5.5 ECOG performance status analysis

The frequency and ratio of the ECOG scores measured before the administration of the investigational medicinal product and at each visit are presented. The McNemar-Bowker test is conducted to assess the changes before and after administration.

## 9. Appendix

Dummy Table

Listing

Definition of Analysis Population

## 10. Reference

Not applicable

## 11. List of Applicable SOPs

| Document ID | Version | Document Title | Classification | Effective Date |
|---|---|---|---|---|
| GEN-GLSOP-SA04-00 | 1.0 | Statistical Analysis | Procedure | 20/May/2020 |
| GEN-GLSOP-SA04-WI01-00 | 2.0 | Statistical Analysis Plan | Procedure | 15/Dec/2020 |
| GEN-GLSOP-SA04-WI02-00 | 1.0 | Statistical Analysis Report | Procedure | 20/May/2020 |
| GEN-GLSOP-SA04-WI03-00 | 2.0 | SAS Programming | Procedure | 28/Feb/2020 |
| GEN-GLSOP-SA04-WI04-00 | 3.0 | Quality Control of Statistical Analysis | Procedure | 15/Dec/2020 |
| GEN-GLSOP-SA04-WI06-00 | 1.0 | Delivery of Deliverables | Procedure | 20/May/2020 |
| GEN-KRSOP-SA03-00 | 1.0 | Statistical Analysis process in Korea | Procedure | 16/May/2019 |
| GEN-KRSOP-SA03-01 | 1.0 | Statistical Analysis Plan Template in Korea | Attachment | 16/May/2019 |
| GEN-KRSOP-SA03-02 | 1.0 | Definition of Analysis Population | Attachment | 16/May/2019 |
| GEN-KRSOP-SA03-03 | 1.0 | Analysis Population Approval Form | Attachment | 16/May/2019 |
| GEN-KRSOP-SA03-04 | 1.0 | Analysis Dataset Specification | Attachment | 16/May/2019 |
| GEN-KRSOP-SA03-06 | 1.0 | SAS Program Completion Form | Attachment | 16/May/2019 |
| GEN-KRSOP-SA03-07 | 1.0 | SAS Program Change Log | Attachment | 16/May/2019 |
| GEN-KRSOP-SA03-08 | 1.0 | Statistical Analysis Report Template in Korea | Attachment | 16/May/2019 |
| GEN-KRSOP-SA03-09 | 1.0 | Statistical Analysis Report Check List | Attachment | 16/May/2019 |
| GEN-GLSOP-DA01-06 | 1.0 | Trial Master File Index and Checklist | Attachment | 26/Dec/2019 |
| GEN-GLSOP-DA01-07 | 1.0 | Note to File | Attachment | 26/Dec/2019 |
| GEN-GLSOP-GA10-01 | 1.0 | All Staff Transition Checklist | Attachment | 06/Sep/2019 |
| GEN-GLSOP-PM01-02 | 1.0 | Project Member Contact List | Attachment | 31/Mar/2020 |
| GCP-GLSOP-CO03-10 | 1.0 | Project Specific Training Record | Attachment | 31/Mar/2020 |
