# Supplementary material for: Regorafenib plus nivolumab in unresectable hepatocellular carcinoma: the phase 2 RENOBATE trial
Source: Nat Med. 2024 Feb 19;30(3):699–707. doi: 10.1038/s41591-024-02824-y (PMC10957471; doi:10.1038/s41591-024-02824-y)
Supplement: Supplementary file 2 — Reporting Summary [file 41591_2024_2824_MOESM2_ESM.pdf]

nature portfolio

Corresponding author(s): Changhoon Yoo and June-Young Koh

Last updated by author(s): Jan 9, 2024

Reporting Summary

Nature Portfolio wishes to improve the reproducibility of the work that we publish. This form provides structure for consistency and transparency in reporting. For further information on Nature Portfolio policies, see our Editorial Policies and the Editorial Policy Checklist.

Statistics

For all statistical analyses, confirm that the following items are present in the figure legend, table legend, main text, or Methods section.

n/a	Confirmed	
☐	☒	The exact sample size (*n*) for each experimental group/condition, given as a discrete number and unit of measurement
☐	☒	A statement on whether measurements were taken from distinct samples or whether the same sample was measured repeatedly
☐	☒	The statistical test(s) used AND whether they are one- or two-sided<br>*Only common tests should be described solely by name; describe more complex techniques in the Methods section.*
☐	☒	A description of all covariates tested
☐	☒	A description of any assumptions or corrections, such as tests of normality and adjustment for multiple comparisons
☐	☒	A full description of the statistical parameters including central tendency (e.g. means) or other basic estimates (e.g. regression coefficient) AND variation (e.g. standard deviation) or associated estimates of uncertainty (e.g. confidence intervals)
☐	☒	For null hypothesis testing, the test statistic (e.g. *F*, *t*, *r*) with confidence intervals, effect sizes, degrees of freedom and *P* value noted<br>*Give P values as exact values whenever suitable.*
☒	☐	For Bayesian analysis, information on the choice of priors and Markov chain Monte Carlo settings
☒	☐	For hierarchical and complex designs, identification of the appropriate level for tests and full reporting of outcomes
☐	☒	Estimates of effect sizes (e.g. Cohen's *d*, Pearson's *r*), indicating how they were calculated

Our web collection on statistics for biologists contains articles on many of the points above.

Software and code

Policy information about availability of computer code

Data collection	All single cell capture was performed on a 10x Genomics Chromium Controller. All sequencing was performed on Illumina NovaSeq 6000. All flow cytometry data was collected using BD LSR II. All Multiplex ELISA was performed Luminex MAGPIX.
Data analysis	Software used to analyze the data include: Cell Ranger (v 3.0.1) ,R (v 3.4.1 or v 4.1.3), Seurat (v 4.1.1), ComplexHeatmap(v 3.17), monocle3 (v 1.3.1). CellChat (v 1.5.0), immunarch (v 0.8.0), clusterprofiler (v 4.2.2), enrichplot (v 1.14.2), enrichR, GSEA (v 4.2.3), Prism (v 9.4.1), FlowJo (v 10.9.0).

For manuscripts utilizing custom algorithms or software that are central to the research but not yet described in published literature, software must be made available to editors and reviewers. We strongly encourage code deposition in a community repository (e.g. GitHub). See the Nature Portfolio guidelines for submitting code & software for further information.

Data

Policy information about availability of data

All manuscripts must include a data availability statement. This statement should provide the following information, where applicable:

- Accession codes, unique identifiers, or web links for publicly available datasets
- A description of any restrictions on data availability
- For clinical datasets or third party data, please ensure that the statement adheres to our policy

Patient-related data not included in this paper were generated as part of the clinical trial, and may be subject to patient confidentiality. All requests for raw and

analyzed data and materials should be directed to C.Y. (yooc@amc.seoul.kr) and will be responded in four weeks. The requests will be promptly reviewed by the Asan Medical Center to determine whether the request is subject to any intellectual property or confidentiality obligations. Any data and materials that can be shared will be released via a material transfer agreement. All raw data for single-cell sequencing are deposited in the Gene Expression Omnibus under the following accession no: GSE243572. All analyses were conducted using publicly available software, as detailed in the Methods section. The raw scripts used to generate the scCNV-seq analysis figures presented in this paper are accessible via the Zenodo repository (https://doi.org/10.5281/zenodo.8131764).

Human research participants

Policy information about studies involving human research participants and Sex and Gender in Research.

Reporting on sex and gender	Gender information was not requested on the study consent form. Biological attribute and patient self-reported sex were used to determine the participant sex. Sex was not considered in the study design of the clinical trials the patients were enrolled to, nor in the biological studies conducted on patients progressing on the therapies they received. Sex on the patients included in this analysis are listed in Extended Table 1. In accordance with Nature policy, we did not conduct post hoc-sex and gender-based analysis. Furthermore, we did not have the patient consent to perform such analysis.
Population characteristics	Patients with unresectable hepatocellular carcinoma who did not receive prior systemic chemotherapy. The median age was 61 years (range, 40–79 years), and 31 patients (73.8%) were male (Table 1). Most patients had Barcelona Clinic Liver Cancer stage C (n = 37, 88.1%), hepatitis B virus infection as an etiology of HCC (n = 30, 71.4%), and prior transarterial chemoembolization (n = 35, 83.3%).
Recruitment	Patient recruitment was done at each participating institution based on the inclusion/exclusion criteria in accordance with the study protocol. Recruitment was based on the site database or patient referral. No potential bias regarding recruitment.
Ethics oversight	The protocol was approved by the institutional review board of each participating center: Asan Medical Center, Samsung Medical Center and CHA Bundang Medical Center

Note that full information on the approval of the study protocol must also be provided in the manuscript.

Field-specific reporting

Please select the one below that is the best fit for your research. If you are not sure, read the appropriate sections before making your selection.

☒ Life sciences ☐ Behavioural & social sciences ☐ Ecological, evolutionary & environmental sciences

For a reference copy of the document with all sections, see nature.com/documents/nr-reporting-summary-flat.pdf

Life sciences study design

All studies must disclose on these points even when the disclosure is negative.

Sample size	In the previous phase 3 trials, sorafenib, current standard 1st line therapy, showed the objective response rates (ORR) of 7% (P0) graded by RECIST v1.1. Regorafenib-nivolumab combination regimen might enhance the ORR to 25% (P1). With alpha of two sided 0.05 and power of 90%, 35 patients are needed based on Fleming's single-stage Phase 2 design calculation. Considering 15% of follow-up loss rates, a total 42 patients are needed for this study.
Data exclusions	For the analyses of the clinical trial, there was no patient excluded. For the exploratory analysis, 13 of 42 patients were excluded for single cell RNA sequencing because of the concept of comparing long-term responders and early progressors.
Replication	Replication was not done because of the limited availability of clinical samples.
Randomization	N/A: single arm study
Blinding	N/A: single arm study

Behavioural & social sciences study design

All studies must disclose on these points even when the disclosure is negative.

Study description	

Research sample

Sampling strategy

Data collection

Timing

Data exclusions

Non-participation

Randomization

Ecological, evolutionary & environmental sciences study design

All studies must disclose on these points even when the disclosure is negative.

Study description

Research sample

Sampling strategy

Data collection

Timing and spatial scale

Data exclusions

Reproducibility

Randomization

Blinding

Did the study involve field work? ☐ Yes ☐ No

Field work, collection and transport

Field conditions

Location

Access & import/export

Disturbance

Reporting for specific materials, systems and methods

We require information from authors about some types of materials, experimental systems and methods used in many studies. Here, indicate whether each material, system or method listed is relevant to your study. If you are not sure if a list item applies to your research, read the appropriate section before selecting a response.

Materials & experimental systems

n/a	Involved in the study
☐	☒ Antibodies
☒	☐ Eukaryotic cell lines
☒	☐ Palaeontology and archaeology
☒	☐ Animals and other organisms
☐	☒ Clinical data
☒	☐ Dual use research of concern

Methods

n/a	Involved in the study
☒	☐ ChIP-seq
☐	☒ Flow cytometry
☒	☐ MRI-based neuroimaging

Antibodies

Antibodies used

The following directly conjugated, unconjugated, or secondary antibodies were used to identify cell markers of CD8+ T cells in human PBMCs (clone, manufacturer, and catalog number): rabbit anti-human TCF1/TCF7 at 1:100 dilution (C63D9 Cell Signaling Technology 2203S), donkey anti-rabbit IgG at 1:100 dilution (Poly4064 Biolegend 406410), mouse anti-human CD3-V500 at 1:100 dilution (UCHT1 BD Biosciences 561416), mouse anti-human Ki-67-BV605 at 1:100 dilution (Ki-67 Biolegend 350522), mouse anti-human Perforin-BV711 at 1:100 dilution (dG9 Biolegend 308130), Pembrolizumab (PD-1, Selleckchem A2005), mouse anti-human IgG4 Fc-FITC at 1:100 dilution (HP6025 Southern Biotech 9200-02), mouse anti-human CD45-PerCP-Cy5.5 at 1:100 dilution (HI30 BD Biosciences 564105), mouse anti-human CD14-PE-TR at 1:200 dilution (61D3 eBioscience 61-0149-42), mouse anti-human CD19-PE-TR at 1:200 dilution (HIB19 eBioscience 61-0199-42), mouse anti-human Granzyme B-Alexa Flour 700 at 1:100 dilution (GB11 BD Biosciences 560213), mouse anti-human CD8-APC-H7 at 1:100 dilution (SK1 BD Biosciences 560179).
The following directly conjugated antibodies were used to identify cell markers of monocytes in human PBMCs (clone, manufacturer, and catalog number): mouse anti-human CD45-V450 at 1:100 dilution (HI30 BD Biosciences 560367), mouse anti-human CD11B-BV510 at 1:100 dilution (ICRF44 BD Biosciences 563088), mouse anti-human CD16-BV650 at 1:100 dilution (BD Biosciences 563692), mouse anti-human CD56-BV786 at 1:100 dilution (BD Biosciences 564058), mouse anti-human HLA-DR-PerCP-Cy5.5 at 1:100 dilution (G46-6 BD Biosciences 560652), mouse anti-human CD19-PE-TR at 1:200 dilution (HIB19 eBioscience 61-0199-42), mouse anti-human CD3-APC at 1:100 dilution (UCHT1 BD Biosciences 555335), mouse anti-human CD14-APC-H7 at 1:100 dilution (MϕP9 BD Biosciences 560180).
The following directly conjugated antibodies were used to identify cell markers of in vitro cultured human CD14+ monocytes (clone, manufacturer, and catalog number) : mouse anti-human CD16-BV510 at 1:100 dilution (3G8 BD Biosciences 563829), mouse anti-human CD86-BV711 (2331 BD Biosciences 563158), mouse anti-human HLA-DR-PerCP-Cy5.5 at 1:100 dilution (G46-6 BD Biosciences 552764), mouse anti-human CD19-PE-TR at 1:200 dilution (HIB19 eBioscience 61-0199-42), mouse anti-human TNF-Alexa Flour 700 at 1:100 dilution (MAb11 BD Biosciences 557996), mouse anti-human CD14-APC-H7 at 1:100 dilution (MϕP9 BD Biosciences 560180).

Validation

The available flow cytometry plots and paper citations using the antibody clone were reviewed prior to purchase to determine suitability for experiment. Only antibodies demonstrating clear separation between positive and negative populations were used and validations were performed according to manufacturers' instruction. Prior to experiment, each antibody was serially diluted and used to stain PBMCs from whole blood to determine the optimal dilution and confirm that the proportion of stained cells was consistent with what was anticipated based on published data.

Eukaryotic cell lines

Policy information about cell lines and Sex and Gender in Research

Cell line source(s)

Authentication

Mycoplasma contamination

Commonly misidentified lines
(See ICLAC register)

Palaeontology and Archaeology

Specimen provenance

Specimen deposition

Dating methods

☐ Tick this box to confirm that the raw and calibrated dates are available in the paper or in Supplementary Information.

Ethics oversight

Note that full information on the approval of the study protocol must also be provided in the manuscript.

Animals and other research organisms

Policy information about studies involving animals; ARRIVE guidelines recommended for reporting animal research, and Sex and Gender in Research

Laboratory animals	
Wild animals	
Reporting on sex	
Field-collected samples	
Ethics oversight	

Note that full information on the approval of the study protocol must also be provided in the manuscript.

Clinical data

Policy information about clinical studies
All manuscripts should comply with the ICMJE guidelines for publication of clinical research and a completed CONSORT checklist must be included with all submissions.

Clinical trial registration	NCT04310709
Study protocol	Full trial protocol is provided.
Data collection	Patients were enrolled between JUL-2020 and JAN-2021. (Data cut-off: 18-APR-2022). Data were collected by investigators and research coordinators in the eligible health care facilities.
Outcomes	The primary endpoint was the objective response rate, according to RECIST v1.1. The secondary endpoints included safety profiles according to the National Cancer Institute Common Terminology Criteria for Adverse Events (NCI-CTCAE) v5.0., modified RECIST, progression-free survival (PFS), and overall survival (OS).<br><br>PFS was defined as the period from the start of study treatment to progression according to RECIST 1.1 or death from any cause, whichever occurred first. OS was defined as the period from the initiation of treatment to death from any cause. The Kaplan-Meier method and the log-rank test were used to estimate and compare the survival distribution, respectively. For patients who did not experience PD or death, the date of censoring for PFS was the earliest of the following: 1) patients who did not experienced an event (and were not otherwise censored) at the time of data cut-off or loss to follow-up were censored on the date of their last follow-up; 2) if there was no tumor assessment after starting study treatment, the patients were censored on the date of last clinical assessment; and 3) patients who received subsequent anti-cancer therapy before experiencing an event were censored at the date of their last clinical assessment before initiating subsequent therapy. For OS, if death was not confirmed at the time of data cut-off, patients were censored on the last date of confirmed survival.

Dual use research of concern

Policy information about dual use research of concern

Hazards

Could the accidental, deliberate or reckless misuse of agents or technologies generated in the work, or the application of information presented in the manuscript, pose a threat to:

No	Yes	
☐	☐	Public health
☐	☐	National security
☐	☐	Crops and/or livestock
☐	☐	Ecosystems
☐	☐	Any other significant area

Experiments of concern

Does the work involve any of these experiments of concern:

No	Yes	
☐	☐	Demonstrate how to render a vaccine ineffective
☐	☐	Confer resistance to therapeutically useful antibiotics or antiviral agents
☐	☐	Enhance the virulence of a pathogen or render a nonpathogen virulent
☐	☐	Increase transmissibility of a pathogen
☐	☐	Alter the host range of a pathogen
☐	☐	Enable evasion of diagnostic/detection modalities
☐	☐	Enable the weaponization of a biological agent or toxin
☐	☐	Any other potentially harmful combination of experiments and agents

ChIP-seq

Data deposition

☐ Confirm that both raw and final processed data have been deposited in a public database such as GEO.

☐ Confirm that you have deposited or provided access to graph files (e.g. BED files) for the called peaks.

Data access links<br>*May remain private before publication.*	
Files in database submission	
Genome browser session<br>(e.g. UCSC)	

Methodology

Replicates	
Sequencing depth	
Antibodies	
Peak calling parameters	
Data quality	
Software	

Flow Cytometry

Plots

Confirm that:

☒ The axis labels state the marker and fluorochrome used (e.g. CD4-FITC).

☒ The axis scales are clearly visible. Include numbers along axes only for bottom left plot of group (a 'group' is an analysis of identical markers).

☒ All plots are contour plots with outliers or pseudocolor plots.

☒ A numerical value for number of cells or percentage (with statistics) is provided.

Methodology

Sample preparation	Peripheral blood mononuclear cells (PBMCs) were isolated from whole blood using standard Histopaque (GE Healthcare, Uppsala) density gradient centrifugation. After cryopreservation, PBMCs were thawed and stained.
Instrument	Data collected using BD FACS LSR II.
Software	Data from flow cytometry were analyzed using FlowJo™ software v.10.9.0 (Tree Star)
Cell population abundance	Cell population abundance was reported as a proportion of a specific population (% of CD8+ T, % of PD-1+CD8+ T,% of Total

Cell population abundance | monocyte)

Gating strategy | For all the experiments single cells were gated on a forward scatter height vs forward scatter area plot. Lymphocytes and monocytes were gated on forward vs side scatter plot based on their size and granularity. To exclude a dead cell, LIVE/DEAD Fixable Dead Cell reactive dye+ cells were gated out. CD8+ T cells from PBMCs were gated with CD45+CD14-CD19-CD3+CD8+ cells. Monocytes from PBMCs were gated with CD45+CD19-CD3-CD56-HLA-DR+CD16+orCD14+ cells. In vitro cultured CD14+ monocytes were gated with CD19-HLA-DR+CD14+ cells.

☒ Tick this box to confirm that a figure exemplifying the gating strategy is provided in the Supplementary Information.

Magnetic resonance imaging

Experimental design

Design type

Design specifications

Behavioral performance measures

Acquisition

Imaging type(s)

Field strength

Sequence & imaging parameters

Area of acquisition

Diffusion MRI ☐ Used ☐ Not used

Preprocessing

Preprocessing software

Normalization

Normalization template

Noise and artifact removal

Volume censoring

Statistical modeling & inference

Model type and settings

Effect(s) tested

Specify type of analysis: ☐ Whole brain ☐ ROI-based ☐ Both

Statistic type for inference
(See Eklund et al. 2016)

Correction

Models & analysis

n/a	Involved in the study
☐	☐ Functional and/or effective connectivity
☐	☐ Graph analysis
☐	☐ Multivariate modeling or predictive analysis

Functional and/or effective connectivity

Graph analysis

Multivariate modeling and predictive analysis
